# Synthesis of Tetracyclic Spirooxindolepyrrolidine-Engrafted Hydantoin Scaffolds: Crystallographic Analysis, Molecular Docking Studies and Evaluation of Their Antimicrobial, Anti-Inflammatory and Analgesic Activities

**DOI:** 10.3390/molecules28217443

**Published:** 2023-11-06

**Authors:** Amani Toumi, Faiza I.A. Abdella, Sarra Boudriga, Tahani Y. A. Alanazi, Asma K. Alshamari, Ahlam Abdulrahman Alrashdi, Amal Dbeibia, Khaled Hamden, Ismail Daoud, Michael Knorr, Jan-Lukas Kirchhoff, Carsten Strohmann

**Affiliations:** 1 Laboratory of Heterocyclic Chemistry Natural Product and Reactivity (LR11ES39), Department of Chemistry, Faculty of Science of Monastir, University of Monastir, Monastir 5019, Tunisia; amani.toumi@univ-lorraine.fr; 2 Department of Chemistry, College of Science, Ha’il University, Ha’il 81451, Saudi Arabiaty.alanazi@uoh.edu.sa (T.Y.A.A.);; 3 Laboratory of Analysis, Treatment and Valorization of Environmental Pollutants and Products, Faculty of Pharmacy, University of Monastir, Monastir 5019, Tunisia; amaldbeibia@gmail.com; 4 Laboratory of Bioresources: Integrative Biology and Valorization, Higher Institute of Biotechnology of Monastir, University of Monastir, Monastir 5000, Tunisia; khaled.hamden@issatmh.rnu.tn; 5 Department of Matter Sciences, University of Mohamed Khider, BP 145 RP, Biskra 07000, Algeria; i.daoud@univ-biskra.dz; 6 Laboratory of Natural and Bio-Actives Substances, Faculty of Science, Tlemcen University, P.O. Box 119, Tlemcen 13000, Algeria; 7 Institut UTINAM-UMR CNRS 6213, Université de Franche-Comté, 16 Route de Gray, 25030 Besançon, France; 8 Faculty of Chemistry, Inorganic Chemistry, Technical University Dortmund, Otto-Hahn-Strasse 6, 44227 Dortmund, Germany; jan-lukas.kirchhoff@tu-dortmund.de (J.-L.K.); carsten.strohmann@tu-dortmund.de (C.S.)

**Keywords:** spirooxindoles, hydantoin, anti-inflammatory, analgesic, antimicrobial, crystal structure, molecular docking

## Abstract

In a sustained search for novel potential drug candidates with multispectrum therapeutic application, a series of novel spirooxindoles was designed and synthesized via regioselective three-component reaction between isatin derivatives, 2-phenylglycine and diverse arylidene-imidazolidine-2,4-diones (Hydantoins). The suggested stereochemistry was ascertained by an X-ray diffraction study and NMR spectroscopy. The resulting tetracyclic heterocycles were screened for their in vitro *and* in vivo anti-inflammatory and analgesic activity and for their in vitro antimicrobial potency. In vitro antibacterial screening revealed that several derivatives exhibited remarkable growth inhibition against different targeted microorganisms. All tested compounds showed excellent activity against the *Micrococccus luteus* strain (93.75 µg/mL ≤ MIC ≤ 375 µg/mL) as compared to the reference drug tetracycline (MIC = 500 µg/mL). Compound **4e** bearing a *p*-chlorophenyl group on the pyrrolidine ring exhibited the greatest antifungal potential toward *Candida albicans* and *Candida krusei* (MIC values of 23.43 µg/mL and 46.87 µg/mL, respectively) as compared to Amphotericin B (MIC = 31.25 and 62.50 µg/mL, respectively). The target compounds were also tested in vitro against the lipoxygenase-5 (LOX-5) enzyme. Compounds **4i** and **4l** showed significant inhibitory activity with IC_50_ = 1.09 mg/mL and IC_50_ = 1.01 mg/mL, respectively, more potent than the parent drug, diclofenac sodium (IC_50_ = 1.19 mg/mL). In addition, in vivo evaluation of anti-inflammatory and analgesic activity of these spirooxindoles were assessed through carrageenan-induced paw edema and acetic acid-induced writhing assays, respectively, revealing promising results. In silico molecular docking and predictive ADMET studies for the more active spirocompounds were also carried out.

## 1. Introduction

Non-steroidal anti-inflammatory drugs (NSAIDs) are the most commonly used drugs in clinical practice to relieve arthritis pain and inflammation [1,2]. They generally work by inhibiting cyclooxygenase (COX) and lipoxygenase (LOX) enzymes, thereby preventing the biosynthesis of prostaglandin that protects the gastric mucosa. However, long-term use of these drugs is usually associated with adverse side effects, particularly ulcers and gastrointestinal irritation, but also renal and cardiovascular damage [3,4,5]. Over recent decades, an increasing number of new anti-inflammatory agents have been developed to alleviate the acuteness of inflammatory complications. Nevertheless, most of the currently approved NSAIDs exhibit either undesirable side reactions or drug tolerance [6]. 

On the other side, multidrug-resistant (MDR) pathogens have dramatically expanded all over the globe in recent years and have become a serious public health emergency [7,8,9]. Without effective antimicrobials, infectious diseases caused by microorganisms become increasingly difficult, or even impossible, to treat, increasing the risk of severe illness, disability and death. For instance, resistant *Pseudomonas aeroginosa* and *Staphylococcus* aureus are among the leading factor of infections and increased healthcare costs [10]. The World Health Organization (WHO) estimates that approximately 64% of patients infected with methicillin-resistant *Staphylococcus aureus* (MRSA) are more likely to die than other patients with drug-sensitive infections [11,12]. Therefore, the development of new therapeutic options with a safer profile, greater efficacy and lower side effects remains a challenge for the medicinal chemist. 

**Figure 1:**
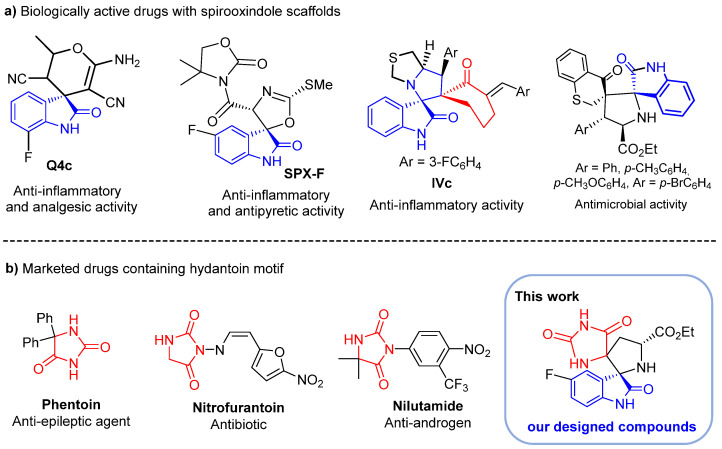
(**a**) Biologically active spirooxindole scaffolds; (**b**) marketed drugs containing the hydantoin motif and our designed compounds.

Spirooxindole derivatives are attention-grabbing compounds due to their miscellaneous use in the medicinal field [13,14,15]. Due to their three-dimensional and flexible scaffolds and, at the same time, highly rigid conformation, these spiroheterocycles are able to potentially bind to various biological targets with high affinity, hence their wide range of actions in many therapeutic areas. These include anesthetics [16], anti-amyloidogenics [17], antidiabetic [18,19], potent mineralocorticoid receptor antagonists [20], dual MDM2 and CDK4 inhibitors targeting glioblastoma [21], anti-cancer agents towards HCT-116 [22] and MCF-7 cancer cells [23]. Multifunctional oxindole-based spiroheterocycles are also promising scaffolds for the discovery of other new therapeutic agents including anti-inflammatory, analgesic and antibiotics. For example, spirocyclic oxindole **Q4c** (Figure 1) exhibits potent in vivo analgesic and anti-inflammatory activity through nitric oxide synthase (NOS) signaling pathway [24]. Spiro[oxazoline-3,3′-oxindole] derivative **SPX-F** (Figure 1) has demonstrated an anti-inflammatory potency and promising antipyretic activity [25]. Spirooxindole-pyrrolothiazole **IVc** (Figure 1) has shown potent anti-inflammatory efficacy with an IC_50_ = 2.4 ± 1.3 μM and was found to be several times more potent than the standard drug, ibuprofen (IC_50_ = 11.2 ± 1.9 μM) [26]. Recently, we reported the synthesis of a new set of spirooxindolopyrrolidine-thiochroman-4-one hybrids **4** (Figure 1) where some derivatives displayed superior antibacterial activity than amoxicillin against *Escherichia coli* and *Bacillus subtilis* and higher antifungal activity than Amphotericin B toward *Candida krusei*, *Candida glabrata* and *Candida albicans* [27]. 

Similarly, hydantoin derivatives (imidazolidine-2,4-diones) [28,29] emerged as privileged scaffolds in medicinal chemistry due to their various biological and therapeutic applications such as anticancer [30,31,32], anti-inflammatory [33,34,35], antidiabetic [36], anticonvulsant [37,38] and antimicrobial agents [39]. With such broad bioactivities, many hydantoin derivatives featured in several clinically used drugs such as phenytoin (anti-epileptic agent) [40], nilutamide (nonsteroidal androgen antagonists) [41] and nitrofurantoin (antibiotic for the treatment of infections caused by multi-drug resistant pathogens) (Figure 1) [42]. They are also frequently encountered in natural molecules and have found a diversity of applications in organic synthesis. 

However, despite the demonstrated therapeutic efficacy inherent in these two specific heterocycles, a limited number of synthetic methods have been reported over the years to access *N*-spiroheterocycles that merge hydantoin and spiropyrrolidine derivatives [43]. Interestingly, the conception of spiropyrrolidines fused to hydantoin and the subsequent exploration of their biological activities as a means to develop novel drug candidates has largely remained untapped. We envisioned that the combination of these distinct heterocycles holds promising potential in the creation of innovative and biologically relevant molecules. The discovery of biologically active compounds embedded in spirooxinole and hydantoin scaffolds prompted us to investigate further modifications within this class of compounds.

**Scheme 1:**
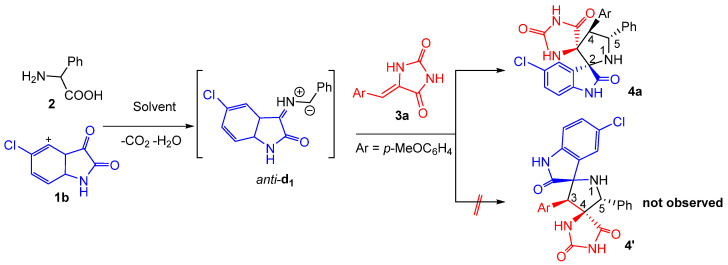
Three component 1,3-dipolar cycloaddition leading to spiropyrrolidine-fused hydantoin hybrid **4a**.

Here, we report the design and synthesis of novel hydantoin-grafted spiroxindoles. The current approach involves one-pot multicomponent reactions (MCRs) of isatin **1** 2-phenylglycine **2** and (*Z*)-5-arylidene 2,4-imidazolidinediones **3** (Scheme 1). The newly synthesized compounds were evaluated against four pathogenic Gram-positive and Gram-negative bacteria and two fungal strains using the in vitro micro diffusion technique. The in vitro and in vivo anti-inflammatory and analgesic activity of the new spirocompounds were also evaluated and the results were further supported by molecular modeling studies to provide a deeper understanding of the plausible binding interactions of the most active compounds. Structure–activity relationship (SAR) trends were also explored to better understand the structural modification substrate scope and extension of the new hybrids. 

## 2. Results and Discussion

### 2.1. Synthetic Chemistry

At the outset of our studies, we performed the three-component reaction between an equivalent amount of chloro-isatin **1b**, 2-phenylglycine **2** with (*Z*)-5-(*p*-methoxybenzylidene) imidazolidine-2,4-dione **3a** as model substrates to optimize the reaction parameters (Scheme 1). The reaction was explored in different solvents and the results were summarized in Table 1. The spirooxindolepyrrolidine **4a** was formed in 70 and 60% yields by performing the reaction in EtOH and CH_3_CN, respectively (Table 1, entries 1 and 5). The yields of the isolated spiroadduct were inferior in 1,4-dioxane, THF, EtOH/H_2_O and toluene (Table 1, entries 2–4 and 6). Gratifyingly, the reaction proceeded smoothly in refluxing the MeOH to furnish the desired *N*-spiroheterocycle **4a** in 85% yield as a single regioisomer (Table 1, entry 5). 

**Scheme 2:**
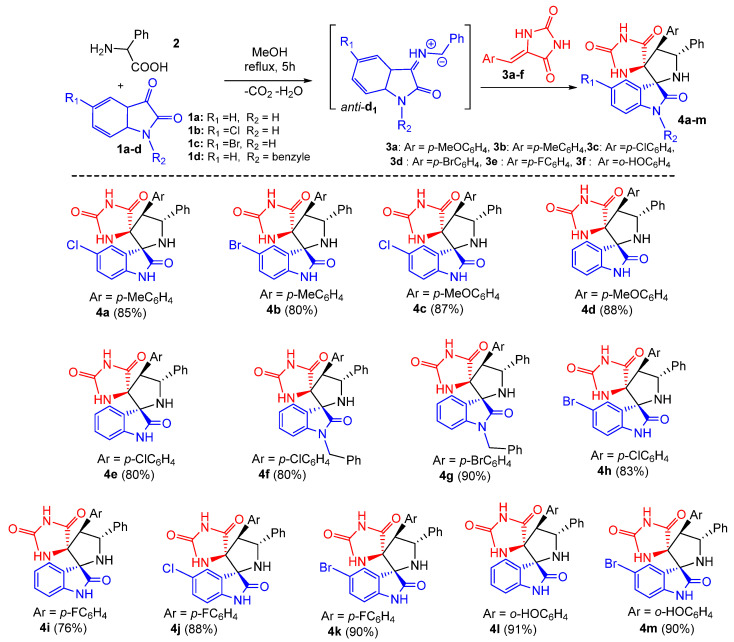
Synthesis of spirooxindolopyrrolidine-hydantoins **4**. Yields of the isolated cycloadducts are given.

Subsequently, the generality of the one pot reaction was examined using several substituted dipolarophiles **3a**–**f** and isatins **1a**–**d**, resulting in the synthesis of diversely substituted products **4a**–**m** in good yields and with high regio- and stereoselectivity (Scheme 2). As shown in Scheme 2, the multicomponent reaction appears to be tolerant towards both electron-donating and electron-withdrawing groups at the *para* position of the aromatic ring of arylidene hydantoins **3**, thereby affording the corresponding products **4a**–**m** in 80–91% yield range (Scheme 2). The system also proceeded well with halogen substituents at the aromatic ring of isatin such as chloro (**1b**) and bromo (**1c**), providing the desired dispiro[imidazolidine-4,3′-pyrrolidine-2′,3″-indoline]-2,2″,5-triones **4** in 76−91% isolated yields (Scheme 2). Furthermore, *N*-protected isatin **1d** was compatible with the reaction.

### 2.2. Spectroscopic and Crystallographic Characterization of the Cycloadducts ***4***

**Figure 2:**
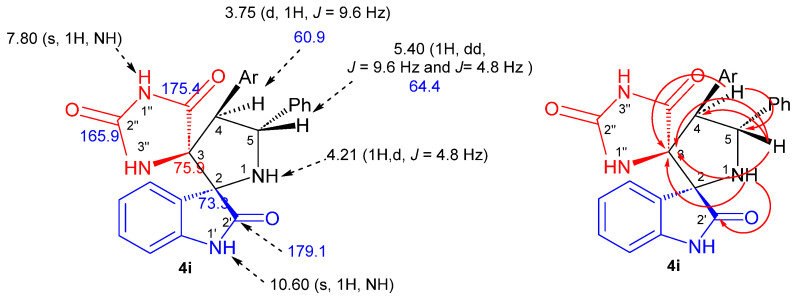
^1^H (black) and ^13^C NMR (blue) chemical shifts along with selected HMBCs (in red) of **4i**.

The structure, the purity and the relative configuration of all products were elucidated from their 1D and 2D NMR spectroscopic data and were unequivocally confirmed by an X-ray data analysis performed on cycloadduct **4i**. Relevant ^1^H and ^13^C chemical shifts of dispiropyrrolidine **4i** are shown in Figure 2.

The ^1^H NMR spectrum of the cycloadduct **4i** shows a doublet at δ H 3.75 (*J* = 9.6 Hz) corresponding to H-4 proton of the pyrrolidine ring which showed ^1^H-^13^C HSQC correlation (Figure S20) with the doublet of a doublets at δ 5.40 ppm (*J* = 9.6 Hz and *J* = 4.8 Hz), attributed to the pyrrolidine proton H-5. The multiplicity and the coupling constant values of these protons confirm the formation of the regioisomer **4i** as in the case of hypothetical regioisomer **4i**′ (Scheme 1), pyrrolidinyl protons should exhibit a singlet instead of a doublet in the ^1^H NMR spectrum. Furthermore, the carbonyl group at 179.1 ppm is attributed to the isatin carbonyl C-2′ based on its heteronuclear multiple-bond correlation (HMBC) (Figure S21) with the H-1 hydrogen of pyrrolidine ring. The two downfield singlets resonating at 10.60 and 7.80 ppm can be attributed to the NH-1′ and NH-1″ hydrogens of the oxindole and hydantoin moieties on the basis of their HMBCs with C-2 and C-3 spirocarbons at 73.3 and 75.9 ppm, respectively, providing further evidence for the proposed structure.

**Figure 3:**
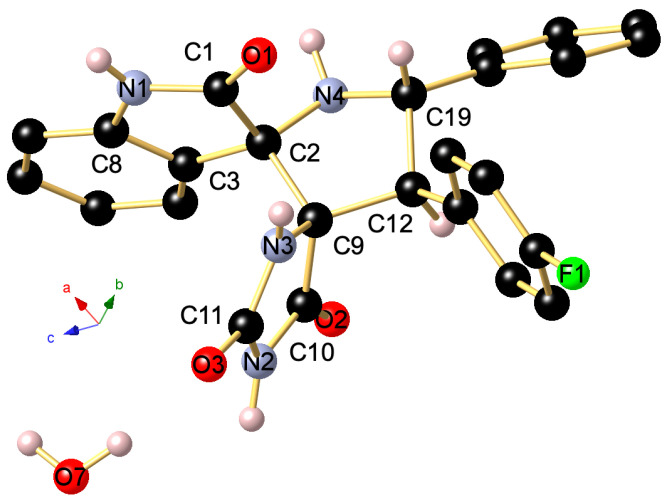
Ball and stick presentation of the molecular structure of **4i** in the crystal. For clarity, only stereochemically significant hydrogen atoms are shown. Only one of the two independent molecules in the unit cell is presented. Selected bond lengths (Å) and angles (°): C1–N1 1.3513(17), C1–O1 1.2278(16), N1–C8 1.4076(17), C1–C2 1.5621(17), C2–C3 1.5066(18), C2–C9 1.5775(17), C2–N4 1.4512(16), C19–N4 1.4604(18), N3–C9 1.4564(16), N3–C11 1.3443(16), C11–N2 1.3892(16), N2–C10 1.3679(17), C11–O3 1.2351(16), C10–O2 1.2136(15), C10–C9 1.5373(17), C9–C12 1.5720(18); C8–N1–C1 111.49(10), N1–C1–C2 108.25(10), C1–C2–N4 114.01(10), N4–C19–C12 101.47(10), C12–C9–N3 117.98(10), N3–C11–N2 108.13(11), C11–N2–C10 111.15(11), N2–C10–C9 107.18(10), C10–C9–N3 100.47(9), C10–C9–C12 110.17(10).

The regio- and stereochemistry of the 1,3-dipolar cycloaddition was further corroborated by a single-crystal X-ray analysis of spiropyrrolidine **4i**, whose molecular structure is depicted in Figure 3. It reveals that the carbonyl carbon of the oxindole component and *(i)* carbonyl group of hydantoin part (C-10 in the crystal structure) are in *trans*-relationship, and *(ii)* a *cis* -relationship with H-5 pyrrolidinyl proton bonded to C-5 (C-19 in the crystal structure). 

Accordingly, we propose that spirooxindole-hydantoins **4** are formed through an *endo*-approach between the *(Z)*-5-arylideneimidazolidine-2,4-diones **3** and the in situ generated (*Z*,*E*)-dipole **d_1_**, as outlined above in Scheme 1 and Scheme 2. 

**Figure 4:**
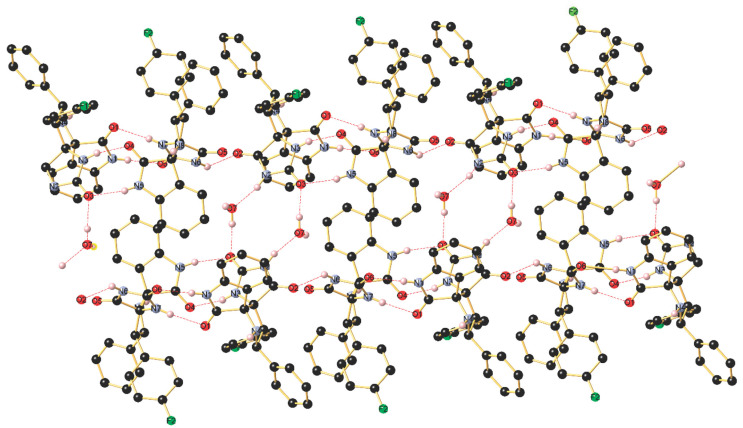
Association of the two independent molecules in the packing through intermolecular N–H···O hydrogen bonding forming a supramolecular 1D array.

The asymmetric unit of **4i** shown in Figure S30 contains two independent molecules (with slightly different bond lengths and angles), crystallizing in the triclinic space group P-1, along with H_2_O molecules. The stereochemistry of the tetracyclic core bearing two additional aryl groups at C19 and C12 is in the solid state in line with the NMR data in solution and establishes the *transoid* arrangement of two the hydrogen atoms of the pyrrolidine-type five-membered cycle. As visualized in Figure S30, the two independent molecules are mutually associated through a strong intermolecular N–H···O=C hydrogen bonding occurring between N1–H1 and O6 with (*d*(N1-H1···O6) 1.96(2) Å), the N1–H1···O6 angle being 173(2)°. Furthermore, a second intermolecular hydrogen bond is formed between N3–H3 and O4 (*d*(N5–H5···O5) 2.05(2) Å), The thus generated supramolecular 1D ribbon displays a third somewhat weaker N–H···O=C bonding between N7–H7 and O1 (*d*(N7–H7···O1) 2.13(2) Å) with a N7–H7···O1 angle being 169.4(19)° as well as a forth interaction between N6–H6 and O23 (*d*(N6–H6···O2) 2.11(2) Å). In addition, the co-crystalized H_2_O molecule interconnects two ribbons through O–H···O bonding with O3 (*d*(O7–H7B···O3) 1.87(3) Å) and N–H···O3 bonding (*d*(N2–H2···O7) 1.88(2) Å). Finally, a weak intermolecular C–H···F1 contact (*d*(C2–H15···F2) 2.49 Å) completes the supramolecular interactions occurring in this highly organized network (Figure 4). 

### 2.3. Biological Activity

#### 2.3.1. In Vitro Lipoxygenase Inhibitory Assay and Structure–Activity Relationship (SAR) Study

Lipoxygenases are a family of monomeric proteins that catalyze oxidation of polyunsaturated fatty acids (PUFA) (linoleic, linolenic and arachidonic acid) to produce hydroperoxides [44,45]. Fractions’ anti-LOX activity was also measured as inhibition of linoleic acid’s peroxidation to hydroperoxylinoleic acid [46].

To evaluate the prospects of the newly synthesized spirooxindoles as promising drug candidates, we first studied their inhibitory potency against the Lipoxygenase-5 (LOX-5) enzyme. The IC_50_ values (concentration causing 50% enzyme inhibition) of the tested compounds and the reference drug diclofenac sodium are recorded in Table 2.

As shown in Table 2, all tested spirooxindoles exhibited modest to potent inhibitory activity (IC_50_ = 1.01–4.96 mg/mL) against LOX-5 enzyme. Interestingly, spirooxindoles **4i** and **4l** (IC_50_ = 1.09 mg/mL and IC_50_ = 1.01 mg/mL, respectively) showed improved LOX-5 inhibition profile better that of diclofenac sodium (IC_50_ = 1.19 mg/mL) (Table 2).

A brief analysis of the SAR of the newly spirooxindole-hydantoin derivatives revealed the following: compounds **4f** (IC_50_ = 4.92 mg/mL) and **4g** (IC_50_ = 4.96 mg/mL) bearing the *N*-benzyl substituent, were comparably less active than the other tested heterocycles. This result could be explained by means of the steric effect of the benzyl group resisted the binding ability with the LOX-5 enzyme. The introduction of halogen substituents (**4e**, **4h**–**k**) or hydroxyl group (**4l**–**m**) at the para position of the aromatic ring of the hydantoin, caused a remarkably increase of the in vitro LOX-5 inhibitory activity. This could be due to the lipophilicity of these groups improving the bioavailability. In addition, compound **4i** and **4l** featuring an unsubstituted isatin ring expressed the highest inhibitory potency. 

#### 2.3.2. Carrageenan-Induced Paw Edema Test

Compounds **4i** and **4l,** which showed the more potent in vitro lipoxygenase inhibitory, were accessed for their in vivo anti-inflammatory activity using carrageenan-induced rat paw edema method as reported by Lopes et al. [47]. Diclofenac sodium was used as a reference drug. This method is used to assess the ability of the tested compounds to reduce the edema produced in the rat paw after being injected with carrageenan (https://en.wikipedia.org/wiki/Carrageenan (accessed on 16 October 2023). Results recorded in Table 3 shows that the injection of carrageenan produced a local edema that increased progressively to reach its maximum after 4h. However, the ingestion of **4i** and **4l** reduced the edema volume by 33% and 32%, respectively, 4 h after the administration of these compounds.

#### 2.3.3. In Vivo Analgesic Assay

The in vivo analgesic activity of the candidate drugs **4i** and **4l** was assessed by the acetic acid-induced writhing test using diclofenac sodium as positive controls as reported previously [49,50,51,52].

Acetic acid injection induces peritoneal inflammation in mice which reacts with a characteristic stretching behavior, called writhing. Any writhing was considered a positive response. The efficacy and potency of analgesic activity of the test compounds is inferred from the reduction in the frequency of writhings [53].

As can be seen in Table 4, the administration of the candidate drugs **4i** and **4l** to acetic acid-induced pain in rats exert good analgesic activity. In fact, the administration by injection of acetic acid to control rats provoked 63 instances of writhing (Table 4). Pre-treatment of rats with **4l, 4i**, and diclofenac sodium at 100 mg/kg reduced the instances of writhing to 36, 29 and 23, respectively (42, 53, and 63% inhibition, respectively).

#### 2.3.4. Acute Toxicity

The acute toxicity test (Table 5) showed that administration of compounds **4i** and **4l** up to a dose of 16.25 mg/kg to rats did not cause any toxicity or physiological or biochemicals changes in the liver (AST and ALT activities) and –kidneys (Albulin, urea and ceat rates) functions. 

#### 2.3.5. Screening the Spirocompounds for Antibacterial and Antifungal Activity 

In this part, the spiropyrrolidines **4a**–**m** were selected to be screening for their antimicrobial activities, using in vitro micro diffusion method, against multiple bacterial strains (*Staphylocoocus aureus* ATCC 25923, *Escherichia coli* ATCC 25922, *Pseudomonas aeruginosa* ATCC 27950, *Micrococcus luteus* NCIMB 8166), and two pathogenic reference yeasts namely *Candida albicans* ATCC 90028 and *Candida kreusei* ATCC 6258. 

**Figure 5:**
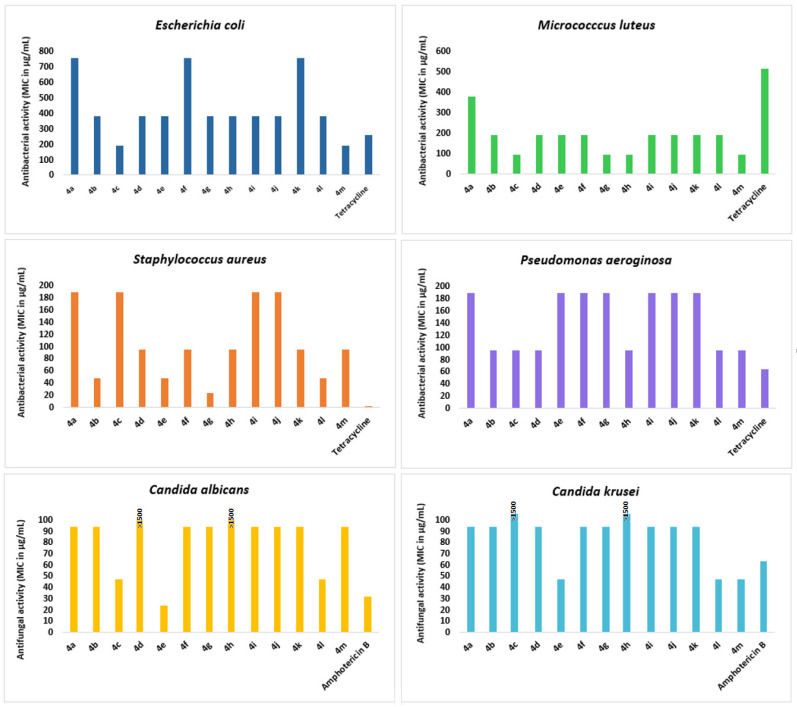
Results of the antibacterial and antifungal activities of the tested spiropyrrolidines against the different tested microorganisms (*Staphylocoocus aureus* ATCC 25923, *Escherichia coli* ATCC 25922, *Pseudomonas aeruginosa* ATCC 27950, *Micrococcus luteus* NCIMB 8166, *Candida albicans* ATCC 90028, and *Candida kreusei* ATCC 6258).

The results showed that all synthesized compounds **4a**–**m** exhibited excellent antibacterial activity against *M. luteus* strain with MIC values ranging from 93.75 to 375 µg/mL. The spiropyrrolidines **4c**, **4h**, **4g**, and **4m** were the most potent derivatives with MIC values of 93.75 µg/mL, as compared to the reference antibiotic Tetracycline (MIC value of 500 µg/mL) (Figure 5). The best activity against *S. aureus* pathogen was obtained for compound **4g** (MIC = 23.34 μg/mL) followed by **4b**, **4e**, and **4l** with MIC value of 46.87 µg/mL. We also noticed a moderate growth inhibition of the tested compounds towards *E. coli* (187.50 μg/mL ≤ MIC ≤ 750 μg/mL) and *P. Aeroginosa* (93.75 μg/mL ≤ MIC ≤ 187.50 μg/mL). Notably, compounds **4c** and **4m** exhibited significant antibacterial activity towards *E. coli* (187.50 µg/mL in MIC), higher than that of Tetracycline (MIC = 256 μg/mL). It is worth noting that compound **4e** demonstrated strong antifungal activity against both the antifungal strains *C. albicans* and *C. krusei* with MIC values of 23.43 and 46.87 µg/mL, in comparison with Amphotericin B (MIC = 31.25 and 62.50 µg/mL, respectively).

To further determine the killing efficiency of the synthesized spiropyrrolidines against selected strains, the minimum bactericidal concentration (MBC) and the minimum fungicidal concentration (MFC) were assessed by the agar method [54]. The results of MBC and MFC assays are summarized in Table 6. 

Interestingly, most of the synthesized molecules exhibited good MBCs results versus Gram-negative pathogen *E. coli*, and compounds **4b, 4d, 4f, 4g, 4h,** and **4m** revealed better efficacy (MBC values in the range of 187.50–374 µg/mL) than the standard tetracycline reference drug (MBC of 512 µg/mL). 

**Figure 6:**
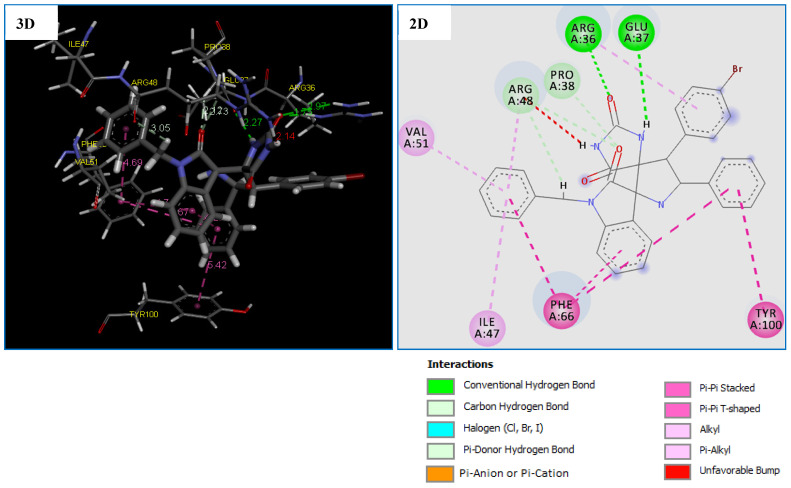
2D and 3D representation of interactions of the compound **4g** occurring within the active site cavity of Thymidylate Kinase (PDB: 4QGG).

Tetracycline was used as reference drug for the antibacterial assay while Amphotericin B was used as a standard antifungal drug. The results are summarized in the histograms presented in Figure 6.

Notably, compounds **4b** and **4c** were effective against the bacterial strain *P. aeruginosa,* while **4h** and **4m** were active against *M. luteus* (MBC = 187.50 µg/mL). However, no significant antibacterial activity was observed towards the Gram-positive bacteria *S. aureus*. The MFC results of the tested fungal strains showed that *C. albicans* is more sensitive to all spiropyrrolidines (except for **4d** and **4h**). The MFC values ranged from 23.43 to 187.50 μg/mL, while for Amphotericin B, it was recorded as 500 μg/mL. Compound 4e also showed a good killing efficacy against the *C. krusei* strain, with an MBC value of 46.87 μg/mL. 

#### 2.3.6. Evaluating the Bactericidal and Bacteriostatic Effects: MBC/MIC Ratio of the Spiropyrrolidines against Microbial Strains

The bactericidal and bacteriostatic action of the spiropyrrolidines **4a**–**m** on selected organisms was determined using their MBC/MIC ratio. The compounds are considered as bactericidal or fungicidal agents when the MBC/MIC or MFC/MIC ratios ≤ 4 and as bacteriostatic or fungistatic if these ratios are >4. All the tested spiropyrrolidines exhibited a bactericidal effect (MBC/MIC ≤ 4) against gram positive and Gram-negative bacteria, except derivatives **4c**, **4g**, and **4h** that demonstrated a bacteriostatic effect (MBC/MIC = 8 > 4) against *M. luteus* pathogen and **4b** showing a bacteriostatic action towards *S. aureus* strain (MBC/MIC = 16). For the fungal strains, the majority of the synthesized heterocycles can be classified as fungicidal agents, especially compounds **4a**, **4b**, **4e**, **4g, 4i, 4k, 4l**, and **4m** which showed MFC/MIC ratios ≤ 4 on both strains *C. Albicans* and *C. Krusei*.

#### 2.3.7. SAR Study

The SAR was established to unravel the important features responsible for the antimicrobial activity of the tested compounds. The influence of different substituents on the isatin nucleus as well as on the aromatic cycle of the dipolarophile was therefore explored. Biological assays results indicated that compounds **4b** and **4d** with electron-donating groups (CH_3_ and OCH_3_) at the *para*-position of phenyl ring exhibit better inhibition against *S. aureus* than derivatives **4e** and **4h** containing chloro substituent attached to the same phenyl ring. Moreover, compounds with electron-donor substituents attached to the phenyl ring of the dipolarophile part generally displayed a higher inhibitory potency than derivatives incorporating electron-withdrawing groups bonded to the identical phenyl ring.

Derivatives that had an electron-donating groups (CH_3_, OCH_3_ and OH) at the *para* and *ortho* positions of the phenyl ring are more effective against Gram-negative bacteria *P. aeroginosa*. For instance, spiropyrrolidines **4b**, **4c**, and **4d** feature electron-donating substituents (CH_3_ and OCH_3_) at the *para*-position of the phenyl ring exhibited better inhibition compared to compounds containing halogen groups (Cl, Br, and F) attached to the same phenyl ring.

For the Gram-negative strain *E. coli*, compounds **4c** and **4m** displayed significant inhibition potency owing to their chloro-substituted isatin nucleus and electron-donating groups (OCH_3_ and OH). Notably, the inhibitory activity is linked to both the substitutions on the dipolarophile and isatin core.

All compounds are more active against *M. luteus* compared to tetracycline, regardless of the nature of the substituents present on both the isatin and the dipolarophiles unit. However, a noteworthy observation is that the activity generally increases by an electron-withdrawing group attached to the isatin core and decreases in the case of unsubstituted isatin- derivatives (**4e**, **4i,** and **4l**). 

### 2.4. Computational Approach

In an attempt to rationalize the observed bioactivity for the newly synthesized derivatives, molecular docking studies were performed. The most antibacterial spirooxindole-hydantoins, i.e., **4c**, **4g**, and **4h** were docked into the active site of three bacterial targets: Thymidylate Kinase (TMK) (PDB ID 4QGG) from *S. aureus*, (3-oxoacyl-[acyl-carrier-protein) synthase 3 (PDB ID 4EWP) from *M. luteus*, DNA gyrase (PDB ID 4DUH) from *E. coli*. The ligand **4e** was docked to the target secreted aspartic protease (PDB ID 3Q70) from *C. albicans* and *C. krusei.* The Ligands **4i** and **4l** with the most promising ant-inflammatory potential were docked into the active sites of LOX-5 (PDB ID: 3V99). The PDB binding structures of different target enzymes are presented in Figures S31–S42 of the Supplementary Material.

#### 2.4.1. MolDock Score and Poses Analysis

The potencies of the most bioactive compounds: **4c**, **4g**, **4h, 4e**, **4i** and **4l** were measured computationally in terms of their MolDock Score. MolDock Score is actually the strength of the intra-molecular interactions among multiple molecules within the binding pockets of selected targets. After having the molecular docking calculation for all **4c**, **4g**, **4h, 4e**, **4i** and **4l** compounds with their selected enzymes, the results obtained were analyzed based on the different parameters such as: Affinity (S-score), Interactions (types and distances). First, if an E–L formed complex gives a low energy score it means that this complex is the most stable also we have high affinity between the compound and target. Second, hydrogen bonds are the most important in the formation of E–L complexes and they can be classified according to the following intervals: If the distance belongs to the interval between 2.5 Å and 3.1 Å, we are considered to be strong interactions. If the distance belongs to the interval between 3.1 Å and 3.55 Å, we are assumed to be weak [55,56,57]. Third, According to C. Janiak et al. [58] who suggested that the optimal range of hydrophobic interactions is between: 3.3–3.8 Å. While other researchers have suggested a relatively higher range [59,60]**.**

#### 2.4.2. Molecular Docking Study of the Antibacterial Targets 

The results obtained after the docking calculations and the best pose received for the compounds **4c**, **4g**, and **4h** with the bacterial targets have been grouped in Table 7.

##### Orientation and Bonding Interaction Compound **4g** in the Active Site Cavity of Thymidylate Kinase (PDB: 4QGG)

The molecular docking results shows that the compound **4g** is the most potent antibacterial agent against TMK (ID: 4QGG) from *S. aurues*, revealing the highest negative score of −153.700 kcal/mol, comparable to the standard drug Tetracycline with the score of −112.465 kcal/mol (Table 7). Comparatively, compound **4g** being the most active against the antibacterial agent against TMK (ID: 4QGG) from *S. aurues* which showed that was formed almost the same number of interactions with the TMK protein of *S. aureus* compared to the Tetracycline with a small difference in the type of residue (Table 7).

Compound **4g** displayed five strong hydrogen bonds (two Conventional H-Bond types and three Carbon H-Bond types) with crucial residues: ARG36 (1.74 Å), GLU37 (2.26 Å), PRO38 (2.12 Å) and ARG48 (2.73 and 3.05 Å) of TMK protein from *S. aureus* (Table 7 and Figure 6). As illustrated in Figure 6, two 6-rings of the compound **4g** displayed two hydrophobic interactions (Pi–Pi T-shaped) with the residues PHE66 and TYR100. In addition, two other hydrophobic interactions (Pi-Pi Stacked) were formed between compound **4g** and residue PHE66. While four hydrophobic interactions (Pi-Alkyl) with active site residues: ARG36, ILE47, ARG48 and VAL51 (Table 7 and Figure 6). We note that these results confirmed by previous studies [61,62,63] which concluded that inhibition of the both amino groups: ARG48, PHE66 and GLU37 of TMK protein might be responsible for their potent antibacterial activity.

##### Orientation and Bonding Interaction Compound **4h** in the Active Site Cavity of (3-Oxoacyl-[acyl-carrier-protein) Synthase (PDB: 4EWP)

**Figure 7:**
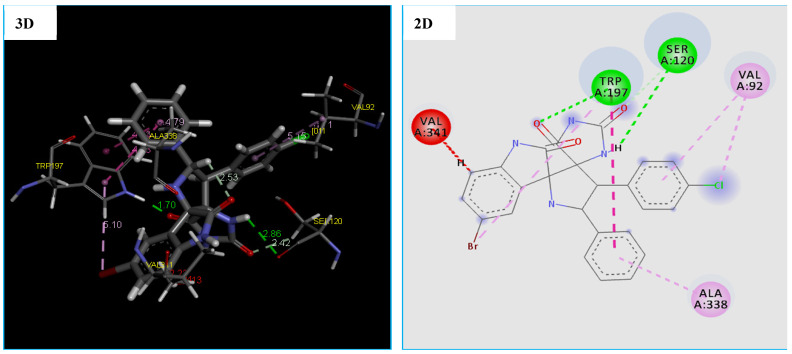
2D and 3D representation of interactions of the compound **4h** occurring within the active site cavity of (3-oxoacyl-[acyl-carrier-protein) synthase (PDB: 4EWP).

Due to the existence of only one crystal structure of the *M. luteus* receptor in the RCSB Protein Data Bank. Initially, we faced a great challenge to identify the active site of the receptor (PDB ID 4EWP) for molecular docking analysis. After solving that problem, we launched molecular docking simulation and as presented in Table 7, the compound **4h** fits well in the binding pocket of *M. luteus* receptor (PDB ID 4EWP) with highest negative score of −147.875 kcal/mol, which is comparable of Tetracycline with a score of −96.245 kcal/mol. Moreover, the docking conformation of compound **4h** showed that this compound established three hydrogen bonds and five hydrophobic interactions with active site residues of the target protein (Figure 7). The hydrogen bonds were observed between the compound and active site residues SER120(2.58 Å), TRP197(1.70 Å) and SER120(2.41 Å) of the target protein, (3-oxoacyl-[acyl-carrier-protein) synthase (Figure 7). While five hydrophobic interactions with active site residues: two with TRP197, two with VAL92 and one with ALA338 were observed (Table 7 and Figure 7). On the other hand, the results of the docking thus indicated a slight difference in the binding of the compound **4h** to the (3-oxoacyl-[acyl-carrier-protein) synthase enzyme vs. the Tetracycline. Furthermore, previous papers [64,65] confirmed that the residues Val92 and ALA338 are responsible for the formation of complex: 4EWP-compounds.

##### Orientation and Bonding Interaction Compound **4c** in the Active Site Cavity of DNA Gyrase (PDB: 4DUH)

**Figure 8:**
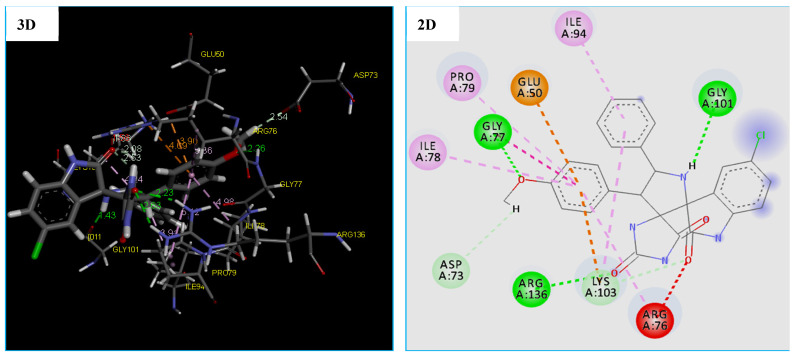
2D and 3D representation of interactions of the compound **4c** occurring within the active site cavity of DNA gyrase (PDB: 4DUH).

The obtained results revealed that the compound **4c** binds into the DNA Gyrease enzyme. The MolDock Score energy and types of possible interactions in addition to, the interacting moiety of our docked compound is represented in Table 7 and Figure 8.

**Figure 9:**
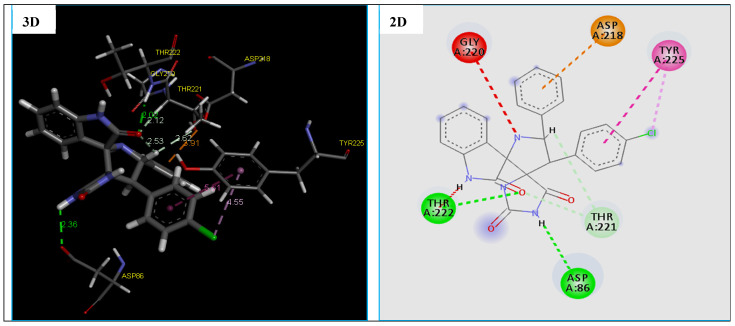
2D and 3D representation of interactions of the compound **4e** occurring within the active site cavity of SAPs (PDB: 3Q70).

Furthermore, the compound **4c** proved a good ability to the binding into DNA Gyrase binding site and this is confirmed by forming 6 strong hydrogen bonding interactions with Gly 101(1.43 Å), Arg 136(2.63 Å and 2.23 Å), Gly 77(2.26 Å), and Lys 103(1.86 Å). As shown from the obtained data compound, **4c** was able to form 6 hydrophobic interactions with different amino acids in the DNA Gyrase binding pocket which are ARG76, LYS103, ILE94, PRO79, ILE78, and GLY77. In addition, the compound **4c** showed two electrostatic interactions (Pi-Anion and Pi-Cation) with GLU50 and LYS103, as displayed by orange dotted lines in Figure 9 for compound **4c** (Table 7 and Figure 8).

Asp 73(2.54 Å) (Table 2 and Figure 8). Also, molecular docking studies revealed that the compound **4c** has many binding modes with a high affinity to DNA gyrase binding site, showing a lowest MolDock Score −139.692 kcal/mol compared to Tetracycline with the MolDock Score of −108.370 kcal/mol (Table 7). However, the results of the docking thus indicated a slight difference in the binding of the compound **4c** to the DNA gyrease enzyme vs. the tetracycline. There are four important amino acids that are common between them, which are mentioned in recent studies [66,67,68].

#### 2.4.3. Molecular Docking Study of the Antifungal Target

The results obtained after the docking simulations and the best pose received for the compound **4e** with the fungal target are given in Table 8.

The docking results reveal that compound **4e** being the most potent antifungal agent displaying a good docking MolDock Score of −129.886 kcal/mol and many molecular interactions with secreted aspartic protease (SAPs) enzyme from *C. Albicans* vs. of the standard drug Amphotericin B with the score of −112.504 kcal/mol. Also, we note that this compound fits well in the binding pocket of the secreted aspartic protease (SAPs) (fungal), which showed good interactions. In fact, compound **4e** established four strong hydrogen bonds, two hydrophobic interactions and one electrostatic interaction with active site residues of the target protein (Table 8 and Figure 9).

The hydrogen bonds were observed between the compound **4e** and active site residues ASP86(2.36 Å), THR222(2.02 Å) and THR221(2.11 Å, 2.61 Å) of the target protein, the secreted aspartic protease (SAPs) (Figure 9). While two hydrophobic interactions with active site residues TYR225, and one electrostatic interaction (Pi-Anion) with ASP218 was observed (Figure 9). Nevertheless, the results of the docking thus indicate a slight difference in the type of residues involve the formation of the binding of the compound **4e** with the secreted aspartic protease (SAPs) vs. the Amphotericin B, which are confirmed in previous research [69,70].

#### 2.4.4. Molecular Docking Study of the Anti-Inflammatory Targets 

**Figure 10:**
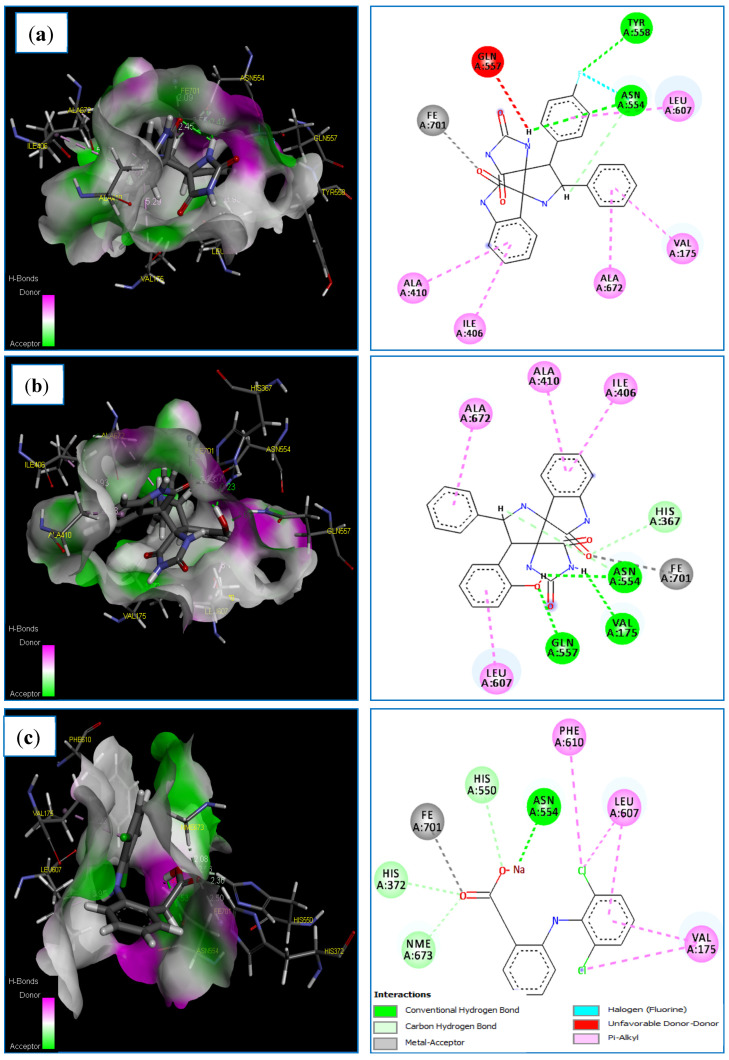
The main interactions of **4i**, **4l** and diclofenac sodium with residues in the binding site of human lipoxygenase 5-LOX: (**a**) 3D and 2D representations of the 3V99-**4i** complex; (**b**) 3D and 2D representations of the 3V99-**4l** complex; (**c**) 3D and 2D representations of the 3V99- Diclofinac sodium complex.

Molecular docking of the compounds **4i**, **4l** and Diclofinac sodium was carried out (Table 9). Regarding the docking score values, we found that the compounds: **4i** and **4l** fit well in the binding active site of the LOX-5 target. As can be observed, the complexes formed by these compounds have a low score energy values of −60.838 and −116.487 kcal/mol, respectively (Table 9). Additionally, docking results revealed that the 3V99-A12 complex shows a good docking MolDock score of −116.487 kcal/mol compared to the standard drug Diclofinac sodium with a score of −102.441 kcal/mol. On the other hand, the two compounds **4i** and **4l** establish interactions of almost the same type as that formed by the standard drug Diclofinac sodium (Table 9). The docked conformation of the compounds **4i** and **4l** are illustrated in Figure 10. Regarding the obtained results, it is apparent that compound **4i** formed three strong hydrogen bonds with active site residue of 5-LOX target, two conventional H-bonds types, F/TYR558(A)-NH/bond distance = 2.79 Å and H/ASN554(A)-OD1/bond distance = 2.47 Å, another one carbon H-bonds H/ASN554(A)-OD1/bond distance = 2.54 Å (Table 9 and Figure 10). A halogen bond occurs between the fluorine atom of compound *A7* and ASN554(A). During which, this compound formed binding with the Fe^2+^ ion of the enzyme’s active site.

In addition, the same compound established five hydrophobic interactions (*π*-alkyl interactions) with the following residues: ILE406 (A), ALA410 (A), LEU607 (A), VAL175 (A) and ALA672(A) (Table 9 and Figure 10a). In this regards, several studies [71,72,73] have revealed that that ASN554(A) and TYR558(A) play a central role in the inhibition of LOX-5 target. Moreover, we note clearly that the compound **4l** establishes more binding interactions with active site residues of 5-LOX target compared to compound **4i**. Five strong hydrogen bonds were formed between compound **4l** and the pocket site of 5-LOX target, three conventional H-bonds, O/GLN557(A)-HE22/bond length = 1.67 Å, H/VAL175(A)-O/bond length = 1.91 Å and H/ASN554(A)-OD1/bond length = 2.23 Å. Additionally, another two carbon H-bonds, O/HIS367(A)-HE1/bond distance = 2.33 Å, and H/ASN554(A)-OD1/bond length = 2.57 Å. This compound indicates direct binding with the Fe^2+^ ion of the enzyme’s active site, and a metal–ligand interaction was observed in this case. Finally, compound **4l** exhibits four hydrophobic interactions (*π*-alkyl interactions) with the following residues: ILE406(A), ALA410(A), LEU607(A), LEU607(A) and ALA672(A) (Table 9 and Figure 10b). These results are supported by a number of studies [74,75], which have indicated that the residues: ALA410(A), ILE406(A) and LEU607(A) represent the major key for the formation of the hydrophobic interactions with the candidates.

#### 2.4.5. *ADME-T* and Drug-Likeness Prediction

ADME-T and drug-likeness parameters were calculated using both online servers SwissADME server (http://www.swissadme.ch/, accessed on 20 May 2023) and the pkCSM server (http://biosig.unimelb.edu.au/pkcsm/prediction, accessed on 20 May 2023), and all results are given in Table 10.

It can be seen from Table 10 that the number of hydrogen bond donors of the all compounds (**4c**, **4g**, **4h, 4e**, **4i** and **4l**) is: <5 (n-HD: (0~7)) and the number of hydrogen bond acceptors is <10 (n-HA: (0~10). Furthermore, all these compounds have molecular weights ranging from 100 to 500 g/mol except compounds **4g** and **4h**, and MLogP and WLogP values are <5. In addition, we found nROTB values <11, indicating that these compounds are flexible. Also, all TPSA values obtained were below 140 Å. Finally, these results show that all compounds satisfy all criteria of drug-likeness without presenting any violation of the Lipinski, Veber and Egan rules. Evidently, no problems are caused in oral bioavailability and pharmacokinetic parameters of either compound.

In addition, the analysis in Table 10 shows that all compounds (**4c**, **4g**, **4h, 4e**, **4i** and **4l**) have good Caco-2 permeability (Caco-2 values greater than −5.15 cm/s). Furthermore, it was evidenced that all compounds had HIA values greater than 30%, indicating that these compounds were administered orally, and that they are strongly absorbed by the gastrointestinal system into the bloodstream of the human body.

According to data from this table, we can see that logPS values for both compounds **4g** and **4h** are >−2 which means that these compounds are able to penetrate the CNS. On the other hand, we note that the other compounds (**4c**, **4e**, **4i** and **4l**) are not able because of their weak logPS values (Table 10). Additionally, the logBB values of the compounds **4h** and **4c** are −1.09 and −1.08, respectively, which are less than −1 (<−1), this indicates that both compounds are poorly distributed to the brain, comparable to other compounds (**4g** and **4e**, **4i** and **4l***)* which are poorly distributed.

Data from this table indicate that all compounds are inhibitors of CYP2C19 isoform and CYP3A4 substrate. Furthermore, neither compound is a CYP1A2 inhibitor nor is it a CYP2D6 substrate (Table 10). Further analysis of this Table showed that all compounds were unlikely OCT2 substrates, and they have a low excretion clearance (<5 mL/min/kg). Finally, we found that all compounds are not likely to be hERG (I and II) Inhibitors and they show no AMES toxicity (Table 10).

## 3. Materials and Methods

### 3.1. Apparatus and General Information

The NMR (400 MHz for ^1^H NMR, 101 MHz for ^13^C {^1^H} NMR) spectra were recorded on Bruker Avance 400 machine (Rheinstetten, Germany). The chemical shifts δ were reported in ppm relative to tetramethylsilane (TMS). Data were described as follows: chemical shift (δ in ppm), multiplicity (s = singlet, d = doublet, m = multiplet, dd = doublet of doublet), coupling constants (Hz), integration. Optical rotations were determined by a Perkin Elmer polarimeter. Elemental analyses were performed on a Perkin Elmer 2400 Series II Elemental CHNS analyzer (Waltham, MA, USA). Thin-layer chromatography (TLC) was performed on silica gel plates (Merck, silica gel 60 F_254_ 0.2 mm, 200 × 200 nm) (Darmstadt, Germany) using UV light at 254 nm.

### 3.2. General Procedure for Preparation of Cycloadducts ***4***

A mixture of 3-arylideneimidazolidine-2,4-dione **3** (1 mmol), 2-phenylglycine **2** (1 mmol) 2, and isatin **1** (1 mmol), in methanol (5 mL) was heated under reflux for 5 h. The solvent was subsequently removed under vacuum. The residue was chromatographed on silica gel employing ethylacetate-cyclohexane (4:6 *v*/*v*) as eluent, with the addition of few drops of methanol to obtain the pure products **4**.

### 3.3. Crystal Structure Determination

Data collection was performed on a Bruker D8 Venture four-circle diffractometer from Bruker AXS GmbH (Karlsruhe, Germany). CPAD detectors used were Photon II from Bruker AXS GmbH; X-ray sources: Microfocus source IμS; and microfocus source IμS Mo and Cu, respectively, from Incoatec GmbH with mirror opticsHELIOS and a single-hole collimator from Bruker AXS GmbH. Programs used for data collection were APEX4 Suite [76] (v2021.10-0) and integrated programs SAINT (V8.40A; integration) and SADABS (2018/7; absorption correction) from Bruker AXS GmbH [76]. The SHELX programs were used for further processing [77]. The solution of the crystal structures was done with the help of the program SHELXT [78], the structure refinement with SHELXL [79]. The processing and finalization of the crystal structure data was done with program OLEX2 v1.5 [80]. All non-hydrogen atoms were refined anisotropically. For the hydrogen atoms, the standard values of the SHELXL program were used with U_iso_ (H) = −1.2 U_eq_(C) for CH_2_ and CH and with U_iso_(H) = −1.5 U_eq_(C) for CH_3_. All H atoms were refined freely using independent values for each U_iso_(H).

Crystal data for C_25_H_20_FN_4_O_3.5_, M = 451.45 g∙mol^−1^, red-orange plates, crystal size 0.942 × 0.789 × 0.281 mm^3^, triclinic, space group *P-1*, a = 12.1123(9) Å, b = 12.5291(10) Å, c = 15.0421(11) Å, α = 95.793(3)°, *β* = 91.329(3)°, *γ* = 99.674(3); V = 2236.9(3) Å^3^, *Z* = 4, D_calc_ = 1.341 g/cm^3^, T = 100 K, *R*_1_ = 0.0509, *wR*_2_ = 0.1291 for 59257 reflections with I ≥ 2σ (I) and 14895 independent reflections. GOF = 1.052. Largest diff. peak/hole/e Å^−3^ 0.61/−0.33. Data were collected using graphite monochromated MoK_α_ radiation λ = 0.71073 Å and have been deposited at the Cambridge Crystallographic Data Centre as CCDC 2248684. (Supplementary Materials). The data can be obtained free of charge from the Cambridge Crystallographic Data Centre via http://www.ccdc.cam.ac.uk/getstructures, accessed on 10 May 2023. 

### 3.4. Anti-Inflammatory and Analgesic Bioassays

Lipoxygenase (sigma-437996-500U) activity was determined by the protocol of by Choudhary et al. [81]. Analgesic activity in rats was determined by the protocol described by Tiss et al. [82]. In vivo anti-inflammatory activity was determined by the carrageenan-induced paw edema method as described by Cordaro et al. [83]. The indices of hepatic toxicity (the activities of AST and ALT) and the level of albumin, urea and creatinine were calculated according to the protocol described by the supplier (Kits Biolabo, Maizy, France).

### 3.5. Computational Approach

#### 3.5.1. Ligands and Targets Preparations

The 3D structures of four most active compounds: **4c**, **4g**, **4h, 4e**, **4i** and **4l** were pre-optimized by means of the Molecular Mechanics using Force Field MM+. After that, all compounds optimized using the semi-empirical method AM1 [84] which implemented in the Hyperchem 8.0.8 software [85].

The X-ray crystallography structures of four different drug target proteins were downloaded from the RCSB Protein Databank (https://www.rcsb.org/, accessed on 20 May 2023), accessed on 10 May 2023 that is, Thymidylate Kinase (TMK) (PDB ID 4QGG) from *S. aureus* [86], (3-oxoacyl-[acyl-carrier-protein) synthase 3 (PDB ID 4EWP) from *M. luteus* [65], DNA gyrase (PDB ID 4DUH) from *E. coli* [87] were selected as antibacterial targets, and secreted aspartic protease (PDB ID 3Q70) from *C. albicans* and *C. krusei* [88] was selected as the antifungal targets. The crystal structure of the human 5-LOX enzyme in complex with its substrate, Arachidonic acid PDB (PDB ID: 3V99) [89], was also downloaded from the same Protein Data Bank. Some information related to the selected targets was reported in Table S1 in the Supplementary Materials). According to Table S1, we note X-ray crystals of the targets selected have resolution between 1.5 and 2.5 Å [90], confirming that these structures can be considered of good quality. 

#### 3.5.2. Molecular Docking Simulations

Docking simulations were carried out on the five targets selected by using Molegro Virtual Docker v. 6.0.1(MVD) [91] software package knowing that the algorithm keeping the macromolecule rigid while allowing ligand flexibility. All X-ray crystals of the targets selected were simplified by removing water molecules, ions, cofactors and co-crystal ligands from their PDB structure and molecular docking protocol were followed and used in our previous studies [92,93]. In the first, The MolDock score [GRID] algorithm was used as the score function, and the MolDock was used as search algorithm. In the second, default parameter settings in the same software package (Score function: MolDock Score; Ligand evaluation: Internal ES, Internal HBond, Sp2–Sp2 Torsions, all checked; Number of runs: 10 runs; Algorithm: MolDock SE; Maximum Interactions: 1500; Max. population size: 50; Max. steps: 300; Neighbor distance factor: 1.00; Max. number of poses returned: 5). The docking procedure was performed using a GRID of 15_A in radius and 0.30 in resolution to cover the ligand-biding site of all targets selected [94]. 

#### 3.5.3. Method Validation

Re-docking of the co-crystal ligands was performed to validate the applied molecular docking approach. We found that the root mean square deviation (RMSD) values variation are between 1 and 2 Å [95] for all complexes Enzyme-co-crystal which means that the docking method is accuracy and successful.

#### 3.5.4. ADME-Tox Evaluation 

Validation of drug-likeness rules, namely Lipinski, Veber and Egan, were conducted by calculating different parameters of physicochemical properties (TPSA, nROT, MW, LogP, number of hydrogen bond acceptors (nHA), and number of hydrogen bond donors (nHD)), by using the SwissADME server (http://www.swissadme.ch/ accessed on 20 May 2023) [96]. On the other hand, pkCSM server (http://biosig.unimelb.edu.au/pkcsm/prediction, accessed on 20 May 2023) [97] is used for ADMET profiles analysis by calculating the following parameters: absorption (Caco-2: colon adenocarcinoma, HIA: Human intestinal absorption), distribution (CNS: central nervous system permeability, BBB: blood–brain barrier permeability), metabolism (CYP1A2 inhibitor, CYP2C19 inhibitor, CYP2D6 inhibitor, etc.), excretion (renal OCT2 substrate: organic cation transporter 2, total clearance), and toxicity (hERG: human ether-à-go-go-related gene, and AMES toxicity).

## 4. Conclusions

In summary, a novel series of spirooxindole-hydantoins was successfully synthesized as potent anti-inflammatory, analgesic, and antimicrobial agents. Antimicrobial screening revealed that *(i)* compounds **4c**, **4h**, **4g,** and **4m** (MIC value of 93.75 µg/mL) were more potent than the reference antibiotic tetracycline (MIC value of 500 µg/mL), against *M. luteus (ii)* compounds **4c** and **4m** showed highest antibacterial activity with a MIC value of 187.50 µg/mL against *E. coli,* and *(iii)* heterocycle **4e** showed the greatest antifungal potential toward *C. albicans* and *C. krusei* (MIC values of 23.43 µg/mL and 46.87 µg/mL, respectively) as compared to amphotericin B (MIC= 31.25 and 62.50 µg/mL, respectively). On the other hand, all tested spirooxindoles exhibited modest to potent inhibitory activity (IC_50_ = 1.01–4.96 mg/mL) against LOX-5 enzyme. Among them, spirooxindoles **4i** and **4l** (IC_50_ = 1.09 mg/mL and IC_50_ = 1.01 mg/mL, respectively) showed improved LOX-5 inhibition profile better that of diclofenac sodium (IC_50_ = 1.19 mg/mL). Moreover, **4i** and **4i** showed a good anti-inflammatory activity (inhibition: 33 and 32%; respectively) which was comparable to the reference standard diclofenac sodium (inhibition: 46%) in carrageenan-induced rat paw edema assay. In addition, they displayed acceptable in vivo analgesic activity in acetic acid-induced writhing response (inhibition: 42 and 53%; respectively) compared to reference drug (63%). Finally, molecular docking simulation and ADME-T prediction revealed that compounds **4g**, **4h**, **4c,** and **4e** have high affinities with the pocket of bacterial and fungal targets, also compounds **4i** and **4l** have high affinities with the binding site of LOX-5 target, which was confirmed by a lowvalue MolDock score and the appearance of several types of interactions which formed between these compounds and residues of the active site of the target selected. Moreover, ADMET predictions and physicochemical properties proved that all rules: Lipinski, Veber and Egan rules were compiled by all compounds tested. Thus, some of newly synthetized spirooxindoles could act as a platform for further evaluation.

## Figures and Tables

**Table 1:** Optimization of the three component 1,3-dipolar cycloaddition reaction ^a^.

Entry	Solvent	t(h)	T(°C)	Yield(%) ^b^
1	EtOH	8	reflux	70
2	EtOH/H_2_O	10	100	36
3	1,4-dioxane	8	80	52
4	THF	8	80	44
5	MeOH	5	reflux	85
6	Toluene	8	80	14
7	CH_3_CN	8	65	60

^a^ All reactions were carried out with **1b** (0.1 mmol), **2** (0.1 mmol), **3a** (0.1 mmol) in solvent (1.0 mL) at specified temperature. ^b^ Isolated yield.

**Table 2:** LOX-5 inhibition results of the synthesized compounds and theirs LogP values.

Comp.	IC_50_ (mg/mL)	LogP *	Comp.	IC_50_ (mg/mL)	LogP	Comp.	IC_50_ (mg/mL)	LogP
**4a**	2.72 ± 0.23	1.44	**4f**	4.92 ± 0.37	0.95	**4k**	1.55 ± 0.11	1.55
**4b**	2.93 ± 0.11	1.55	**4g**	4.96 ± 0.31	3.09	**4l**	1.09 ± 0.13	1.70
**4c**	2.16 ± 0.17	2.37	**4h**	1.66 ± 0.15	3.26	**4m**	1.42 ± 0.15	1.55
**4d**	2.34 ± 0.12	0.95	**4i**	1.01 ± 0.09	2.18	**Diclofenac sodium**	1.19 ± 0.11	3.84
**4e**	1.77 ± 0.11	2.69	**4j**	1.92 ± 0.13	1.44			

* LogP values calculated by SwissADME server (http://www.swissadme.ch/ (accessed on 16 October 2023). Data are mean ± SEM (*n* = 5 each).

**Table 3:** Effect of the compounds **4i** and **4l** on *λ*-carrageenan-induced paw edema in rats.

	Increase in Paw Volume (Time in Hour)				% Inhibition of Edema		
	0 h	1 h	2 h	4 h	1 h	2 h	4 h
**Control**	0.59 ± 0.11	1.31 ± 0.36	1.54 ± 0.18	2.27 ± 0.10	-	-	-
**4i**	0.43 ± 0.14	0.98 ± 0.11	1.13 ± 0.21	1.51 ± 0.13	27	25	33
**4l**	0.57 ± 0.19	0.93 ± 0.13	1.26 ± 0.31	1.73 ± 0.33	29	18	32
**Diclofenac sodium**	0.41 ± 0.11	0.59 ± 0.13	0.83 ± 0.19	1.21 ± 0.39	30	46	46

Compounds **4i** and **4l** were tested in comparison of reference drug diclofenac sodium in dose of 10 mg/kg [48]. Drugs were administrated orally 1 h before carrageenan injection. Data are mean ± SEM (*n* = 5 each).

**Table 4:** Analgesic activity of **4l** and **4i** on acetic acid-induced writhing test.

	Number of Writhing ^a^	% Inhibition
**Control**	63 ± 7	0
**4i**	36 ± 5	42
**4l**	29 ± 6	53
**Diclofenac sodium**	23 ± 4	63

^a^ Values are given as mean ± SE.

**Table 5:** Evaluation of the toxicity of compounds **4i** and **4l** to a dose of 16.25 mg/kg in rats.

	Control	4i	4l
**AST (U/L)**	95 ± 10	98.3 ± 13	101 ± 11
**ALT (U/L)**	18.5 ± 1.2	21.1 ± 1.5	22 ± 1.7
**Albumin (g/L)**	7.71 ± 1.3	8.7 ± 1.66	8.5 ± 1.1
**Urea (mmol/L)**	2.52 ± 0.5	2.83 ± 0.7	2.97 ± 0.5
**Creat (µmol/L)**	3.06 ± 0.2	3.31 ± 0.9	3.41 ± 0.8

**Table 6:** Antibacterial and antifungal activity (MBC and MFC in µg/mL).

Comp.	Bacterial Strains				Yeast Strains	
	Gram-Positive Bacteria		Gram-Negative Bacteria			
	*S. aureus* ATCC 25923	*M. luteus* NCIMB 8166	*E. coli* ATCC 25922	*P. aeruginosa* ATCC 27950	*C. albicans* ATCC 90028	*C. kreusei* ATCC 6258
**4a**	187.50	>1500	>1500	375	93.75	93.75
**4b**	750	750	375	93.75	187.50	93.75
**4c**	187.50	750	187.50	93.75	46.87	>1500
**4d**	93.75	750	375	187.50	>1500	93.75
**4e**	93.75	>1500	750	187.50	23.43	46.87
**4f**	187.50	750	375	375	93.75	93.75
**4g**	46.87	>1500	375	187.50	93.75	93.75
**4h**	187.50	187.50	375	187.50	>1500	>1500
**4i**	750	>1500	750	187.50	93.75	187.50
**4j**	93.75	750	187.50	93.75	93.75	187.50
**4k**	187.50	>1500	187.50	187.50	187.50	187.50
**4l**	93.75	750	187.50	93.75	187.50	93.75
**4m**	93.75	187.50	375	187.50	93.75	93.75
**Tetracycline**	8	512	512	128	-	-
**Amphotericin B**	-	-	-	-	500	62.50

**Table 7:** MolDock Score and interactions between **4c**, **4g,** and **4h** with antibacterial targets.

Antibacterial Targets								
*S. aureus* (PDB ID: 4QGG)								
Comp.	MolDock Score (kcal/mol)		Bonds between Atoms of Compounds and Active Site Residues					
			Atom of Compound	Involved Receptor Atoms	Involved Receptor Residues	Category	Type	Distance (Å)
**4g**	−153.700		O3	HE	ARG36	H-Bond	Conventional H-Bond	1.74
			H16	O	GLU37	H-Bond	Conventional H-Bond	2.26
			O1	HD1	PRO38	H-Bond	Carbon H-Bond	2.12
			O1	HD2	ARG48	H-Bond	Carbon H-Bond	2.73
			H25	O	ARG48	H-Bond	Carbon H-Bond	3.05
			6-ring	6-ring	PHE66	Hydrophobic	Pi-Pi Stacked	5.66
			6-ring	6-ring	PHE66	Hydrophobic	Pi-Pi Stacked	4.68
			6-ring	6-ring	PHE66	Hydrophobic	Pi-Pi T-shaped	4.73
			6-ring	6-ring	TYR100	Hydrophobic	Pi-Pi T-shaped	5.42
			6-ring	/	ARG36	Hydrophobic	Pi-Alkyl	4.99
			6-ring	/	ILE47	Hydrophobic	Pi-Alkyl	5.35
			6-ring	/	ARG48	Hydrophobic	Pi-Alkyl	4.46
			6-ring	/	VAL51	Hydrophobic	Pi-Alkyl	3.87
**Tetracycline**	−112.465		H24	O	PHE66	H-Bond	Conventional H-Bond	2.28
			H18	OE2	GLU37	H-Bond	Conventional H-Bond	2.44
			O8	HH12	ARG70	H-Bond	Conventional H-Bond	3.09
			O4	HH21	ARG70	H-Bond	Conventional H-Bond	2.71
			O8	HG	SER97	H-Bond	Conventional H-Bond	2.02
			H23	OE1	GLN101	H-Bond	Conventional H-Bond	2.05
			H22	O	SER96	H-Bond	Conventional H-Bond	3.05
			N1	HD1	ARG92	H-Bond	Carbon H-Bond	2.97
			6-ring	/	VAL51	Hydrophobic	Pi-Alkyl	4.23
			6-ring	/	ARG48	Hydrophobic	Pi-Alkyl	4.51
			C3	/	PHE66	Hydrophobic	Pi-Alkyl	3.95
			6-ring	/	PHE66	Hydrophobic	Pi-Pi T-shaped	4.29
			/	/	SER69	H-Bond	Unfavorable Bump	/
***M. luteus* (PDB ID:4EWP)**								
**4h**		−147.875	H16	O	SER120	H-Bond	Conventional H-Bond	2.58
			O2	HE1	TRP197	H-Bond	Conventional H-Bond	1.70
			O3	HB2	SER120	H-Bond	Carbon H-Bond	2.41
			6-ring	5-ring	TRP197	Hydrophobic	Pi-Pi stacked	4.43
			6-ring	6-ring	TRP197	Hydrophobic	Pi-Pi stacked	4.02
			6-ring	C	VAL92	Hydrophobic	Pi-Alkyl	5.15
			CL	/	VAL92	Hydrophobic	Pi-Alkyl	4.79
			6-ring		ALA338	Hydrophobic	Alkyl	4.70
			/	/	VAL341	H-Bond	Unfavorable Bump	/
**Tetracycline**		−96.245	H9	O	HIS96	H-Bond	Conventional H-Bond	2.40
			O4	HE22	GLN206	H-Bond	Conventional H-Bond	1.77
			H9	O	PHE94	H-Bond	Conventional H-Bond	2.25
			H10	O	VAL92	H-Bond	Conventional H-Bond	2.18
			H12	O	PHE94	H-Bond	Carbon H-Bond	2.53
			O1	HA	ALA97	H-Bond	Carbon H-Bond	2.91
			6-ring	6-ring	TRP233	Hydrophobic	Pi-Pi stacked	4.66
			C	5-ring	PRO234	Hydrophobic	Pi-Alkyl	4.79
			/	/	VAL156	H-Bond	Unfavorable Bump	/
			/	/	VAL156	H-Bond	Unfavorable Bump	/
***E. coli* (PDB ID:** **4DUH)**								
**4c**		−139.692	H1	O	GLY101	H-Bond	Conventional H-Bond	1.43
			O3	H11	ARG136	H-Bond	Conventional H-Bond	2.63
			O3	H21	ARG136	H-Bond	Conventional H-Bond	2.23
			O4	HN	GLY77	H-Bond	Conventional H-Bond	2.26
			O1	HE1	LYS103	H-Bond	Carbon H-Bond	1.86
			H19	OD1	ASP73	H-Bond	Carbon H-Bond	2.54
			/	OE1	GLU50	Electrostatic	Pi-Anion	3.90
			C	C	ARG76	Hydrophobic	Pi-Alkyl	5.36
			/	NZ	LYS103	Electrostatic	Pi-Cation	4.69
			/	/	LYS103	Hydrophobic	Pi-Alkyl	4.24
			/	/	ILE94	Hydrophobic	Pi-Alkyl	3.91
			/	/	PRO79	Hydrophobic	Pi-Alkyl	5.11
			/	/	ILE78	Hydrophobic	Pi-Alkyl	4.97
			/	C	GLY77	Hydrophobic	Amide-Pi stacked	3.59
			/	/	ARG76	H-Bond	Unfavorable Bump	/
**Tetracycline**		−108.37	H18	OD1	ASN46	H-Bond	Conventional H-Bond	2.40
			O3	HN	VAL120	H-Bond	Conventional H-Bond	2.01
			O4	HN	VAL120	H-Bond	Conventional H-Bond	2.39
			H22	OE1	GLU42	H-Bond	Conventional H-Bond	2.62
			H18	OE1	GLU42	H-Bond	Conventional H-Bond	2.03
			H10	O	GLY101	H-Bond	Conventional H-Bond	1.50
			H16	O	GLY117	H-Bond	Carbon H-Bond	2.70
			H17	O	GLY117	H-Bond	Carbon H-Bond	2.96
			H15	O	LYS103	H-Bond	Carbon H-Bond	2.66
			H13	O	HIS99	H-Bond	Carbon H-Bond	2.89
			O8	HA	ASN46	H-Bond	Carbon H-Bond	2.79
			/	NZ	LYS103	Electrostatic	Pi-Cation	3.91
			6-ring	C	LYS103	Hydrophobic	Pi-Alkyl	3.22
			6-ring	/	ILE78	Hydrophobic	Pi-Alkyl	4.99
			/	/	ILE94	H-Bond	Unfavorable Bump	/

**Table 8:** MolDock Score and interactions between the compounds **4e** with fungal target.

Antifungal Targets							
*C. albicans and C. krusei* (PDB ID: 3Q70)							
**4e**	**−129.886**	H12	OD2	ASP86	H-Bond	Conventional H-Bond	2.36
		O1	HN	THR222	H-Bond	Conventional H-Bond	2.02
		O1	HA	THR221	H-Bond	Carbon H-Bond	2.11
		H18	OG1	THR221	H-Bond	Carbon H-Bond	2.61
		CL1	6-ring	TYR225	Hydrophobic	Pi-Alkyl	4.55
		6-ring	6-ring	TYR225	Hydrophobic	Pi-Pi stacked	5.60
		/	OD2	ASP218	Electrostatic	Pi-Anion	3.91
		/	/	GLY220	H-Bond	Unfavorable Bump	/
		/	/	THR222	H-Bond	Unfavorable Bump	/
**Amphotericin B**	−112.504	H27	O	GLY220	H-Bond	Conventional H-Bond	2.49
		O8	HN	GLY85	H-Bond	Conventional H-Bond	2.17
		O3	HG	SER88	H-Bond	Conventional H-Bond	3.05
		H16	OD1	ASP86	H-Bond	Conventional H-Bond	1.94
		H40	OE1	GLN121	H-Bond	Conventional H-Bond	1.93
		H48	OD2	ASP120	H-Bond	Conventional H-Bond	1.63
		C21	6-ring	TYR225	Hydrophobic	Pi-Alkyl	5.12
		C35	C	VAL12	Hydrophobic	Alkyl	4.42
		/	/	GLN11	H-Bond	Unfavorable Bump	/
		/	/	GLN11	H-Bond	Unfavorable Bump	/

**Table 9:** Molecular docking results for the compounds **4i**, **4l** and diclofenac sodium involved in 5-LOX target.

5-LOX (PDB ID: 3V99)							
Compounds	MolDock Score (kcal/mol)	Bonds between Atoms of Compounds and Active Site Residues					
		Atom of Compound	Involved Receptor Atoms	Involved Receptor Residues	Category	Type	Distance (Å)
**4i**	−60.838	F	HN	TYR558(A)	Hydrogen Bond	Conventional H-Bond	2.79
		H	OD1	ASN554(A)	Hydrogen Bond	Conventional H-Bond	2.47
		H	OD1	ASN554(A)	Hydrogen Bond	Carbon H-Bond	2.54
		O	FE	FE701	Other	Metal-Acceptor	2.09
		F	O	ASN554(A)	Halogen	Halogen (Fluorine)	3.66
		6-ring	/	ILE406(A)	Hydrophobic	Pi-Alkyl	5.16
		6-ring	/	ALA410(A)	Hydrophobic	Pi-Alkyl	5.11
		6-ring	/	LEU607(A)	Hydrophobic	Pi-Alkyl	3.97
		6-ring	/	VAL175(A)	Hydrophobic	Pi-Alkyl	5.28
		6-ring	/	ALA672(A)	Hydrophobic	Pi-Alkyl	5.06
**4l**	−116.487	O	HE22	GLN557(A)	Hydrogen Bond	Conventional H-Bond	1.67
		H	O	VAL175(A)	Hydrogen Bond	Conventional H-Bond	1.91
		H	OD1	ASN554(A)	Hydrogen Bond	Conventional H-Bond	2.23
		O	HE1	HIS367(A)	Hydrogen Bond	Carbon H-Bond	2.33
		H	OD1	ASN554(A)	Hydrogen Bond	Carbon H-Bond	2.57
		O	FE	FE701	Other	Metal-Acceptor	2.40
		6-ring	/	ILE406(A)	Hydrophobic	Pi-Alkyl	4.92
		6-ring	/	ALA410(A)	Hydrophobic	Pi-Alkyl	4.98
		6-ring	/	LEU607(A)	Hydrophobic	Pi-Alkyl	3.78
		6-ring	/	ALA672(A)	Hydrophobic	Pi-Alkyl	4.74
**Diclofinac sodium**	−102.441	Na	OD1	ASN554(A)	Hydrogen Bond	Conventional H-Bond	2.53
		O	HE1	HIS272(A)	Hydrogen Bond	Carbon H-Bond	2.36
		O	HE1	HIS550(A)	Hydrogen Bond	Carbon H-Bond	2.56
		O	HH32	NME673(A)	Hydrogen Bond	Carbon H-Bond	2.08
		O	FE	FE701	Other	Metal-Acceptor	2.49
		Cl	/	VAL175(A)	Hydrophobic	Alkyl	4.23
		Cl	/	LEU607(A)	Hydrophobic	Alkyl	3.95
		Cl	/	PHE610(A)	Hydrophobic	Pi-Alkyl	5.49
		/	/	VAL175(A)	Hydrophobic	Pi-Alkyl	3.93
		/	/	LEU607(A)	Hydrophobic	Pi-Alkyl	5.43

**Table 10:** ADME and drug-likeness properties of the compounds **4c**, **4g**, **4h, 4e**, **4i** and **4l**.

**Entry**	**TPSA Å^2^**		**n-ROTB**		**MW**	**MLog P**	**n-ON** **Acceptors**	**n-OHNH** **Donors**	**Lipinski’s** **Violations**	**Veber** **Violations**	**Egan** **Violations**
						**WLog P**					
	**<140**		**<11**		**<500**	**≤5**	**<10**	**<5**	**≤1**	**≤1**	**≤1**
**4g**	90.54		2		582.24	3.09	4	3	Accepted	Accepted	Accepted
						2.05					
**4h**	99.33		2		537.79	3.26	4	4	Accepted	Accepted	Accepted
						1.81					
**4c**	108.56		3		488.92	2.37	5	4	Accepted	Accepted	Accepted
						1.06					
**4e**	99.33		2		458.90	2.69	4	4	Accepted	Accepted	Accepted
						1.05					
**4i**	99.33		2		442.44	2.18	5	2	Accepted	Accepted	Accepted
						0.95					
**4l**	119.56		2		440.45	1.70	5	2	Accepted	Accepted	Accepted
						0.10					
**ADME-T**	**Absorption**		**Distribution**		**Metabolism**			**Excretion**		**Toxicity**	
	**Caco2** **(10^−6^ cm/s)**	**HIA** **%**	**CNS** **(Log PS)**	**BBB** **(Log BB)**	**CYP1A2 Inhibitor**	**CYP2C19 Inhibitor**	**CYP2D6 Substrate**	**Renal OCT2** **Substrate**	**Total** **Clearance** **(mL/min/kg)**	**AMES Toxicity**	**hERG I and II** **Toxicity**
**4g**	0.757	91.63	−1.93	−0.92	NO	YES	NO	NO	0.59	NO	NO
**4h**	−0.074	85.30	−1.99	−1.09	NO	YES	NO	NO	0.55	NO	NO
**4c**	0.007	85.88	−2.32	−1.08	NO	YES	NO	NO	0.62	NO	NO
**4e**	−0.033	84.45	−2.13	−0.08	NO	YES	NO	NO	0.64	NO	NO
**4i**	0.607	88.0	−2.31	−0.36	NO	YES	NO	NO	0.70	NO	NO
**4l**	0.729	77.7	−2.40	−0.81	NO	YES	NO	NO	0.72	NO	NO

**ABS:** Absorption, **TPSA:** Topological Polar Surface Area**, n-ROTB:** Number of Rotatable Bonds**, MW:** Molecular Weight**, MLog *P*:** logarithm of partition coefficient of compound between n-octanol and water**, n-ON acceptors:** Number of hydrogen bond acceptors**, n-OHNH donors:** Number of hydrogen bonds donors. **Caco-2:** Colon adenocarcinoma**, HIA:** Human intestinal absorption**, CNS:** *CENTRAL NERVOUS SYSTEM* permeability**, BBB:** Blood–Brain Barrier permeability.

## Data Availability

Not applicable.

## Supplementary Materials

The following supporting information can be downloaded at: https://www.mdpi.com/article/10.3390/molecules28217443/s1, compound characterization data of spirooxindoles-hydantoins **4a**–**m**. Copies of NMR spectra of compounds **4a**–**m** (Figures S1–S29). Association of two independent molecules of **4i** (Figure S30). Information related to the studied enzyme in the docking studies (Table S1). Molecular docking study of the antibacterial and antifungal **4c**. PDB binding structures of target enzymes (Table S2 and Figures S31–S42). Crystallographic CIF file and Check-CIF. Table S3. MolDock Score and interactions between the **4e** with antifungal target.
