# Supplementary material for: Synthesis of Tetracyclic Spirooxindolepyrrolidine-Engrafted Hydantoin Scaffolds: Crystallographic Analysis, Molecular Docking Studies and Evaluation of Their Antimicrobial, Anti-Inflammatory and Analgesic Activities
Source: Molecules. 2023 Nov 6;28(21):7443. doi: 10.3390/molecules28217443 (PMC10650415; doi:10.3390/molecules28217443)

checkCIF/PLATON report

Structure factors have been supplied for datablock(s) mo_b1515_0m

THIS REPORT IS FOR GUIDANCE ONLY. IF USED AS PART OF A REVIEW PROCEDURE FOR PUBLICATION, IT SHOULD NOT REPLACE THE EXPERTISE OF AN EXPERIENCED CRYSTALLOGRAPHIC REFEREE.

No syntax errors found. CIF dictionary Interpreting this report

Datablock: mo_b1515_0m

```
Bond precision:   C-C = 0.0021 A           Wavelength=0.71073

Cell:           a=12.1123(9)       b=12.5291(10)     c=15.0421(11)
                alpha=95.793(3)    beta=91.329(3)    gamma=99.674(3)
Temperature:    100 K

                 Calculated                    Reported
Volume           2236.9(3)                     2236.9(3)
Space group      P -1                          P -1
Hall group       -P 1                          -P 1
Moiety formula   2(C25 H19 F N4 O3), H2 O      C25 H19 F N4 O3, 0.5(H2 O)
Sum formula      C50 H40 F2 N8 O7              C25 H20 F N4 O3.50
Mr               902.90                        451.45
Dx,g cm-3        1.340                         1.341
Z                2                             4
Mu (mm-1)        0.097                         0.097
F000             940.0                         940.0
F000'            940.46
h,k,lmax         17,18,22                      17,18,22
Nref             15062                         14895
Tmin,Tmax        0.913,0.973                   0.510,0.565
Tmin'            0.913

Correction method= # Reported T Limits: Tmin=0.510 Tmax=0.565
AbsCorr = MULTI-SCAN

Data completeness= 0.989            Theta(max)= 31.616

                                                  wR2(reflections)=
R(reflections)= 0.0509( 12486)                    0.1365( 14895)

S = 1.052                  Npar= 644
```

The following ALERTS were generated. Each ALERT has the format
test-name_ALERT_alert-type_alert-level.
Click on the hyperlinks for more details of the test.

Alert level B

```
PLAT420_ALERT_2_B D-H Bond Without Acceptor  O7       --H7C      .     Please Check
```

Alert level C

```
PLAT042_ALERT_1_C Calc. and Reported MoietyFormula Strings  Differ     Please Check
PLAT420_ALERT_2_C D-H Bond Without Acceptor  N8       --H8       .     Please Check
PLAT906_ALERT_3_C Large K Value in the Analysis of Variance ......      2.535 Check
PLAT911_ALERT_3_C Missing FCF Refl Between Thmin & STh/L=    0.600         52 Report
```

Alert level G

```
PLAT045_ALERT_1_G Calculated and Reported Z Differ by a Factor ...      0.500 Check
PLAT063_ALERT_4_G Crystal Size Possibly too Large for Beam Size ..       0.94 mm
PLAT154_ALERT_1_G The s.u.'s on the Cell Angles are Equal ..(Note)      0.003 Degree
PLAT793_ALERT_4_G Model has Chirality at C2          (Centro SPGR)          S Verify
PLAT793_ALERT_4_G Model has Chirality at C9          (Centro SPGR)          R Verify
PLAT793_ALERT_4_G Model has Chirality at C12         (Centro SPGR)          R Verify
PLAT793_ALERT_4_G Model has Chirality at C19         (Centro SPGR)          S Verify
PLAT793_ALERT_4_G Model has Chirality at C27         (Centro SPGR)          S Verify
PLAT793_ALERT_4_G Model has Chirality at C34         (Centro SPGR)          R Verify
PLAT793_ALERT_4_G Model has Chirality at C37         (Centro SPGR)          R Verify
PLAT793_ALERT_4_G Model has Chirality at C44         (Centro SPGR)          S Verify
PLAT910_ALERT_3_G Missing # of FCF Reflection(s) Below Theta(Min).          4 Note
PLAT912_ALERT_4_G Missing # of FCF Reflections Above STh/L=  0.600        110 Note
PLAT933_ALERT_2_G Number of HKL-OMIT Records in Embedded .res File          7 Note
PLAT941_ALERT_3_G Average HKL Measurement Multiplicity ..........         4.0 Low
PLAT978_ALERT_2_G Number C-C Bonds with Positive Residual Density.         21 Info
```

0 **ALERT level A** = Most likely a serious problem - resolve or explain
1 **ALERT level B** = A potentially serious problem, consider carefully
4 **ALERT level C** = Check. Ensure it is not caused by an omission or oversight
16 **ALERT level G** = General information/check it is not something unexpected

3 ALERT type 1 CIF construction/syntax error, inconsistent or missing data
4 ALERT type 2 Indicator that the structure model may be wrong or deficient
4 ALERT type 3 Indicator that the structure quality may be low
10 ALERT type 4 Improvement, methodology, query or suggestion
0 ALERT type 5 Informative message, check

It is advisable to attempt to resolve as many as possible of the alerts in all categories. Often the minor alerts point to easily fixed oversights, errors and omissions in your CIF or refinement strategy, so attention to these fine details can be worthwhile. In order to resolve some of the more serious problems it may be necessary to carry out additional measurements or structure refinements. However, the purpose of your study may justify the reported deviations and the more serious of these should normally be commented upon in the discussion or experimental section of a paper or in the "special_details" fields of the CIF. checkCIF was carefully designed to identify outliers and unusual parameters, but every test has its limitations and alerts that are not important in a particular case may appear. Conversely, the absence of alerts does not guarantee there are no aspects of the results needing attention. It is up to the individual to critically assess their own results and, if necessary, seek expert advice.

Publication of your CIF in IUCr journals

A basic structural check has been run on your CIF. These basic checks will be run on all CIFs submitted for publication in IUCr journals (*Acta Crystallographica*, *Journal of Applied Crystallography*, *Journal of Synchrotron Radiation*); however, if you intend to submit to *Acta Crystallographica Section C* or *E* or *IUCrData*, you should make sure that full publication checks are run on the final version of your CIF prior to submission.

Publication of your CIF in other journals

Please refer to the *Notes for Authors* of the relevant journal for any special instructions relating to CIF submission.

PLATON version of 18/05/2022; check.def file version of 17/05/2022

Datablock mo_b1515_0m - ellipsoid plot

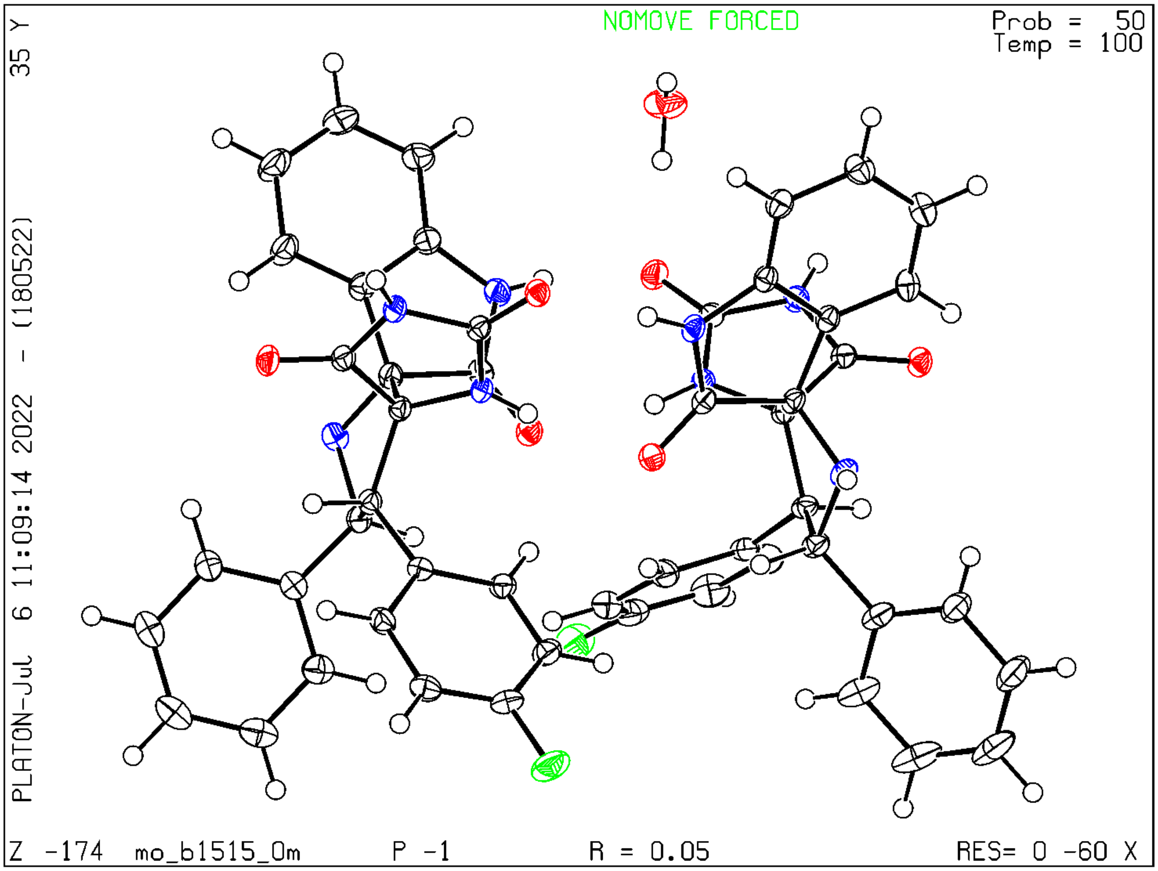

Supplement: Supplementary file 1 [file molecules-28-07443-s001.zip › checkcif B1515.pdf]
