# Supplementary material for: Synthesis of Tetracyclic Spirooxindolepyrrolidine-Engrafted Hydantoin Scaffolds: Crystallographic Analysis, Molecular Docking Studies and Evaluation of Their Antimicrobial, Anti-Inflammatory and Analgesic Activities
Source: Molecules. 2023 Nov 6;28(21):7443. doi: 10.3390/molecules28217443 (PMC10650415; doi:10.3390/molecules28217443)
Supplement: Supplementary file 1 [file molecules-28-07443-s001.zip › Supporting Material molecules-2659847.pdf]

Supplementary Material

Table of contents

	Page
1. Characterization data of compounds **4a-m**	2-4
2. Copies of NMR spectra of compounds **4a-m** (S1-S29)	5-19
3. View of the asymmetric unit of **4i** (S30)	19
4. Molecular docking study	
4.1. Table S1. Information related to the studied enzyme in the docking studies	20
4.2. Molecular docking study of the antibacterial and antifungal targets	20-23
4.3. PDB binding structures of target enzymes (S31-S42)……………………………………..	24-26

1. Characterization data of compounds 4a-m

(2′R*,4S*,4′S*,5′R*)-5''-chloro-5'-phenyl-4'-(p-tolyl)dispiro[imidazolidine-4,3'-pyrrolidine -2',3''-indoline]-2,2'',5-trione (4a)

White solid; Yield: (400 mg, 85%); mp (°C ± 2) = 240 °C; ^{1}H NMR (400 MHz, DMSO-d6) δ 10.60 (s, 1H, $NH_{3''}$), 10.57 (s, 1H, $NH_{1'}$), 7.57 (s, 1H, $NH_{1''}$), 7.53 (d, J = 8.1 Hz, 1H, Ar), 7.44 – 7.40 (m, 2H, Ar), 7.37 (d, J = 7.2 Hz, 2H, Ar), 7.26 – 7.20 (m, 4H, Ar), 7.10 (d, J = 7.9 Hz, 2H, Ar), 6.76 (d, J = 7.9 Hz, 1H, Ar), 5.39 (dd, J = 10.0, 4.8 Hz, 1H, H_5), 4.14 (d, J = 4.8 Hz, 1H, H_1), 3.72 (d, *J* = 10.0 Hz, 1H, H_4), 2.33 (s, 3H, CH_3); ^{13}C NMR (101 MHz, DMSO) δ 179.2 ($C_{2'}$), 175.4 ($C_{5''}$), 155.7 ($C_{2''}$), 142.4, 142.3, 138.7, 136.9, 132.5, 132.1, 131.0, 130.5, 130.4, 129.9, 129.8, 129.6, 128.7, 127.6, 113.6, 109.1, 75.7 (C_3), 73.3 (C_2), 64.1 (C_5), 61.2 (C_4), 21.5 (CH_3); Anal. Calcd for $C_{26}H_{21}ClN_4O_3$: C, 66.03; H, 4.48; N, 11.85%. Found: C, 66.05; H, 4.49; N, 11.84%.

(2′R*,4S*,4′S*,5′R*)-5''-bromo-5'-phenyl-4'-(p-tolyl)dispiro[imidazolidine-4,3'-pyrrolidine -2',3''-indoline]-2,2'',5-trione (4b)

White solid; Yield: (412 mg, 80%); mp (°C ± 2) = 236 °C; ^{1}H NMR (400 MHz, DMSO) δ 10.49 (s, 1H, $NH_{3''}$), 10.48 (s, 1H, $NH_{1'}$), 7.67 (s, 1H, $NH_{1''}$), 7.51 (dd, J = 9.8, 5.2 Hz, 2H, Ar), 7.40 (d, J = 7.2 Hz, 2H, Ar), 7.30 (dd, J = 8.1, 5.9 Hz, 2H, Ar), 7.23 – 7.19 (m, 3H, Ar), 7.10 (d, J = 8.0 Hz, 2H, Ar), 6.83 (d, J = 8.3 Hz, 1H, Ar), 5.43 (dd, J = 10.4, 4,8 Hz, 1H, H_5), 4.13 (d, *J* = 4.8 Hz, 1H, H_1), 3.80 (d, *J* = 10.0 Hz, 1H, H_4), 2.33 (s, 3H, CH_3); ^{13}C NMR (101 MHz, DMSO) δ 178.8 ($C_{2'}$), 175.4 ($C_{5''}$), 155.4 ($C_{2''}$), 142.2, 138.0, 136.6, 132.1, 131.2, 130.8, 130.1, 129.8, 129.3, 129.3, 128.7, 128.2, 127.9, 127.2, 113.6, 108.5, 75.7 (C_3), 73.4 (C_2), 63.9 (C_5), 61.3 (C_4), 20.9 (CH_3); Anal. Calcd for $C_{26}H_{21}BrN_4O_3$: C, 60.36; H, 4.09; N, 10.83%. Found: C, 60.39; H, 4.11; N, 10.81%.

(2′R*,4S*,4′S*,5′R*)-5''-chloro-4'-(4-methoxyphenyl)-5'-phenyldispiro[imidazolidine-4,3'-pyrrolidine-2',3''-indoline]-2,2'',5-trione (4c)

White solid; Yield: (424 mg, 87%); mp (°C ± 2) = 211 °C; ^{1}H NMR (400 MHz, DMSO) δ 10.57 (s, 1H, $NH_{3''}$), 10.57 (s, 1H, $NH_{1'}$), 7.62 – 7.57 (m, 2H, $NH_{1''}$), 7.48 (d, J = 2.1 Hz, 1H, Ar), 7.38 (d, J = 7.3 Hz, 2H, Ar), 7.31 – 7.18 (m, 5H, Ar), 6.97 (d, J = 8.7 Hz, 2H, Ar), 6.83 (dd, J = 15.5, 8.5 Hz, 2H, Ar), 5.34 (dd, J = 10.1, 5.0 Hz, 1H, H_5), 4.11 (d, J = 5.0 Hz, 1H, H_1), 3.77 – 3.75 (m, 1H, H_4), 3.72 (s, 3H, OCH_3); ^{13}C NMR (101 MHz, DMSO) δ 179.3 ($C_{2'}$), 175.4 ($C_{5''}$), 159.9 ($C_{2''}$), 156.1, 142.3, 141.9, 131.6, 130.8, 129.6, 128.7, 127.6, 127.6, 126.5, 125.9, 114.8, 114.3, 111.3, 109.3, 75.7 (C_3), 73.4 (C_2), 64.3 (C_5), 60.8 (C_4), 55.7 (OCH_3); Anal. Calcd for $C_{26}H_{21}ClN_4O_4$: C, 63.87; H, 4.33; N, 11.46%. Found: C, 63.86; H, 4.30; N, 11.47%.

(2′R*,4S*,4′S*,5′R*)-4'-(4-methoxyphenyl)-5'-phenyldispiro[imidazolidine-4,3'-pyrrolidine-2',3''-indoline]-2,2'',5-trione (4d)

White solid; Yield: (400 mg, 88%); mp (°C ± 2) = 187 °C; ^{1}H NMR (400 MHz, DMSO) δ 10.43 (s, 2H, NH), 7.60-7.58 (m, 3H, Ar+NH), 7.50 (d, J = 7.5 Hz, 2H, Ar), 7.37 (t, J = 7.0 Hz, 2H, Ar), 7.26 – 7.20 (m, 3H, Ar), 6.98 – 6.96 (m, 3H, Ar), 6.85 (d, J = 8.7 Hz, 1H, Ar), 5.37 (dd, J = 10.0, 4.4 Hz, 1H, H_5), 3.93 (d, J = 4.8 Hz, 1H, H_1), 3.76 (d, J = 10.0 Hz, 1H, H_4), 3.72 (s, 3H, OCH_3); δ ^{13}C NMR (101 MHz, DMSO) δ 178.5 ($C_{2'}$), 174.8 ($C_{5''}$), 155.1 ($C_{2''}$), 142.0, 141.4, 134.3, 132.2, 132.1, 131.3, 130.2, 129.9, 128.2, 127.5, 127.1, 120.7, 113.1, 111.3, 75.1 (C_3), 73.0 (C_2), 63.8 (C_5), 60.3 (C_4), 55.8 (OCH_3); Anal. Calcd for $C_{26}H_{22}N_4O_4$: C, 68.71; H, 4.88; N, 12.33%. Found: C, 68.72; H, 4.86; N, 12.35%.

(2′R*,4S*,4′S*,5′R*)-4'-(4-chlorophenyl)-5'-phenyldispiro[imidazolidine-4,3'-pyrrolidine-2',3''-indoline]-2,2'',5-trione (4e)

White solid; Yield: (366 mg, 80%); mp (°C ± 2) = 235 °C; ^{1}H NMR (300 MHz, DMSO) δ 10.45 (s, 1H, $NH_{3''}$), 10.38 (s, 1H, $NH_{1'}$), 7.52 (d, J = 7.3 Hz, 1H, Ar+NH), 7.45 – 7.34 (m, 7H, Ar), 7.21 – 7.01 (m, 4H, Ar), 6.99 (t, J = 7.3 Hz, 1H, Ar), 6.81 (d, J = 7.5 Hz, 1H, Ar), 5.42 (dd, J = 10.0, 5.7 Hz, 1H, H_5), 4.21 (d, J = 5.7 Hz, 1H, H_1), 3.84 (d, J = 10.0 Hz, 1H, H_4); ^{13}C NMR (75 MHz, DMSO) δ 178.8 ($C_{2'}$), 174.6 ($C_{5''}$), 155.2 ($C_{2''}$), 142.6, 141.7, 134.1, 132.1, 131.8, 129.3, 128.3, 128.2, 127.9, 127.4, 127.1, 121.4, 119.2, 109.3, 75.2 (C_3),

73.1(C_2), 63.9 (C_5), 60.3 (C_4); Anal. Calcd for $C_{25}H_{19}ClN_4O_3$: C, 65.43; H, 4.17; N, 12.21%. Found: C, 65.45; H, 4.15; N, 12.20%.

(2′R*,4S*,4′S*,5′R*)-1''-benzyl-4'-(4-chlorophenyl)-5'-phenyldispiro[imidazolidine-4,3'-pyrrolidine-2',3''-indoline]-2,2'',5-trione (4f)

White solid; Yield: (438 mg, 80%); mp (°C ± 2) = 195 °C; ^{1}H NMR (400 MHz, DMSO) δ 10.56 (bs, 1H, $NH_{3''}$), 7.86 (s, 1H, $NH_{1''}$), 7.59 (d, *J* = 6.0 Hz, 1H, Ar), 7.52– 7.42 (m, 2H, Ar), 7.39 – 7.33 (m, 5H, Ar), 7.30 – 7.19 (m, 8H, Ar), 7.04 (t, *J* = 7.5 Hz, 1H, Ar), 6.74 (d, *J* = 8.1 Hz, 1H, Ar), 5.49 (dd, *J* = 9.8, 4.7 Hz, 1H, H_5), 5.10 (d, *J* = 16.0 Hz, 1H, CH_2Bn), 4.70 (d, *J* = 15.8 Hz, 1H, CH_2Bn), 4.21 (d, *J* = 4.8 Hz, 1H, H_1), 3.83 (d, *J* = 10.8 Hz, 1H, H_4); ^{13}C NMR (101 MHz, DMSO) δ 177.0 ($C_{2'}$), 174.5 ($C_{5''}$), 155.0 ($C_{2''}$), 143.6, 137.9, 136.0, 134.7, 133.5, 133.3, 132.8, 132.4, 131.5, 130.0, 129.6, 129. 2, 128.9, 128.5, 127.3, 127.1, 126.1, 114.2, 112.3, 107.3, 75.3 (C_3), 72.4 (C_2), 63.6 (C_5), 60.2 (C_4), 43.0 (CH_2Bn); Anal. Calcd for $C_{32}H_{25}ClN_4O_4$: C, 70.01; H, 4.59; N, 10.20%. Found: C, 70.03; H, 4.61; N, 10.22%.

(2′R*,4S*,4′S*,5′R*)-1''-benzyl-4'-(4-bromophenyl)-5'-phenyldispiro[imidazolidine-4,3'-pyrrolidine-2',3''-indoline]-2,2'',5-trione (4g)

White solid; Yield: (532 mg, 90%); mp (°C ± 2) = 223 °C; ^{1}H NMR (300 MHz, DMSO) δ 10.48 (s, 1H, $NH_{3''}$), 7.67 – 7.54 (m, 3H, Ar+NH), 7.39 (dd, *J* = 13.0, 6.5 Hz, 7H, Ar), 7.26 (dt, *J* = 16.1, 7.5 Hz, 7H, Ar), 7.04 (t, *J* = 7.5 Hz, 1H, Ar), 6.75 (d, *J* = 7.7 Hz, 1H, Ar), 5.50 (dd, *J* = 9.9, 5.3 Hz, 1H, H_5), 5.09 (d, *J* = 15.9 Hz, 1H, CH_2Bn), 4.73 (d, *J* = 15.9 Hz, 1H, CH_2Bn), 4.09 (d, *J* = 5.1 Hz, 1H, H_1), 3.89 (d, *J* = 10.0 Hz, 1H, H_4); ^{13}C NMR (101 MHz, $CDCl_3$) δ 178.2 ($C_{2'}$), 175.7 ($C_{5''}$), 155.3 ($C_{2''}$), 144.0, 134.9, 134.4, 134.4, 132.7, 131.3, 131.3, 130.1, 129.8, 129.3, 129.1, 128.4, 127.8, 127.0, 125.3, 124.5, 123.7, 122.8, 114.8, 110.2, 75.4 (C_3), 72.7 (C_2), 63.9 (C_5), 60.2 (C_4), 42.8 (CH_2Bn); Anal. Calcd for $C_{32}H_{25}BrN_4O_3$: C, 64.76; H, 4.25; N, 9.44%. Found: C, 64.77; H, 4.23; N, 9.45%.

2′R*,4S*,4′S*,5′R*)-5''-bromo-4'-(4-chlorophenyl)-5'-phenyldispiro[imidazolidine-4,3'-pyrrolidine-2',3''-indoline]-2,2'',5-trione (4h)

White solid; Yield: (445 mg, 83%); mp (°C ± 2) = 198 °C; ^{1}H NMR (400 MHz, DMSO) δ 10.64 (bs, 1H, $NH_{3''}$), 10.60 (s, 1H, $NH_{1'}$), 7.79 (s, 1H, $NH_{1''}$), 7.64 (d, J = 8.8 Hz, 2H, Ar), 7.61 – 7.60 (m, 1H, Ar), 7.46 – 7.40 (m, 1H, Ar), 7.38 – 7.35 (m, 1H, Ar), 7.30 – 7.26 (m, 3H, Ar), 7.30 – 7.26 (m, 2H, Ar), 7.23 (d, J = 7.2 Hz, 1H, Ar), 6.77(d, J = 8.4 Hz, 1H, Ar), 5.40 (dd, J = 9.6, 4.8 Hz, 1H, H_5), 4.21 (d, J = 4.8 Hz, 1H, H_1), 3.75 (d, J = 10.0 Hz, 1H, H_4); ^{13}C NMR (101 MHz, DMSO) δ 179.2 ($C_{2'}$), 175.5 ($C_{5''}$), 156.2 ($C_{2''}$), 142.6, 142.1, 134.4, 133.3, 132.6, 132.5, 132.4, 131.5, 130.7, 130.3, 129.2, 129.0, 128.9, 128.7, 128.0, 127.6, 113.6, 111.8, 75.6 (C_3), 73.3 (C_2), 64.4 (C_5), 60.9 (C_4); Anal. Calcd for $C_{25}H_{18}BrClN_4O_4$: C, 55.83; H, 3.37; N, 10.42%. Found: C, 55.81; H, 3.36; N, 10.44%.

(2′R*,4S*,4′S*,5′R*)-4'-(4-fluorophenyl)-5'-phenyldispiro[imidazolidine-4,3'-pyrrolidine-2',3''-indoline]-2,2'',5-trione (4i)

White solid; Yield: (336 mg, 76%); mp (°C ± 2) = 221 °C; ^{1}H NMR (400 MHz, DMSO) δ 10.50 (s, 1H, $NH_{3''}$), 10.46 (s, 1H, $NH_{1'}$), 7.67 (s, 1H, $NH_{1''}$), 7.51 (d, J = 6.8 Hz, 1H, Ar), 7.43 – 7.33 (m, 4H, Ar), 7.26 (d, J = 7.7 Hz, 2H, Ar), 7.24 – 7.18 (m, 2H, Ar), 7.12 (t, J = 8.8 Hz, 2H, Ar), 6.98 (td, J = 7.7, 0.9 Hz, 1H, Ar), 6.79 (d, J = 7.5 Hz, 1H, Ar), 5.38 (dd, J = 10.0, 5.4 Hz, 1H, H_5), 4.00 (d, J = 5.2 Hz, 1H, H_1), 3.80 (d, J = 10.0 Hz, 1H, H_4); ^{13}C NMR (101 MHz, DMSO) δ 179.4 ($C_{2'}$), 175.3 ($C_{5''}$), 160.7, 155.8 ($C_{2''}$), 143.0, 142.3, 132.5, 132. 5, 131. 7, 130.9, 129.9, 128.7, 128.4, 127.9, 127. 6, 121.9, 115.8, 115.6, 109.8, 75.7 (C_3), 73.4 (C_2), 64.5 (C_5), 60.7 (C_4); Anal. Calcd for $C_{25}H_{19}FN_4O_3$: C, 67.87; H, 4.33; N, 12.66%. Found: C, 67.88; H, 4.30; N, 12.64%.

(2′R*,4S*,4′S*,5′R*)-5''-chloro-4'-(4-fluorophenyl)-5'-phenyldispiro[imidazolidine-4,3'-pyrrolidine-2',3''-indoline]-2,2'',5-trione (4j)

White solid; Yield: (419 mg, 88%); mp (°C ± 2) = 208 °C; ^{1}H NMR (300 MHz, DMSO) δ 10.56 (s, 1H, $NH_{3''}$), 10.53 (s, 1H, $NH_{1'}$), 7.61 (s, 1H, $NH_{1''}$), 7.49 (d, *J* = 2.3 Hz, 1H, Ar), 7.39 – 7.34 (m, 4H, Ar), 7.32 – 7.17 (m, 4H, Ar), 7.10 (t, *J* = 8.9 Hz, 2H, Ar), 6.81 (d, *J* = 8.3 Hz, 1H, Ar), 5.37 (dd, *J* = 10.2, 5.1 Hz, 1H, H_5), 4.10 (d, *J* = 5.1 Hz, 1H, H_1), 3.78 (d, *J* = 10.2 Hz, 1H, H_4); ^{13}C NMR (75 MHz, DMSO) δ 178.7 ($C_{2'}$), 174. 8 ($C_{5''}$), 155.1 ($C_{2''}$), 141. 6, 132.0, 131.9, 130.9, 130.9, 130.1, 129.2, 128.2, 127.4, 127.1, 127.1, 126.2,

125.4, 115.3, 115.0, 110.7, 75.1 (C_3), 72.9 (C_2), 64.0 (C_5), 60.2 (C_4); Anal. Calcd for $C_{25}H_{18}ClFN_4O_3$: C, 62.96; H, 3.80; N, 11.75%. Found: C, 62.95; H, 3.82; N, 11.77%.

(2′R*,4S*,4′S*,5′R*)-5''-bromo-4'-(4-fluorophenyl)-5'-phenyldispiro[imidazolidine-4,3'-pyrrolidine-2',3''-indoline]-2,2'',5-trione (4k)

White solid; Yield: (468 mg, 90%); mp (°C ± 2) = 223 °C; ^{1}H NMR (300 MHz, DMSO) δ 10.53 (s, 2H, $NH_{3''}$), 7.62 (d, *J* = 2.0 Hz, 1H, $NH_{1'}$), 7.57 (s, 1H, $NH_{1''}$), 7.55 – 7.45 (m, 3H, Ar), 7.44 – 7.35 (m, 2H, Ar), 7.34 – 7.17 (m, 5H, Ar), 6.78 (d, *J* = 8.3 Hz, 1H, Ar), 5.40 (dd, *J* = 9.9, 5.1 Hz, 1H, H_5), 4.10 (d, *J* = 5.2 Hz, 1H, H_1), 3.78 (d, *J* = 10.0 Hz, 1H, H_4); ^{13}C NMR (75 MHz, DMSO) δ 178.5 ($C_{2'}$), 174.8 ($C_{5''}$), 155.1 ($C_{2''}$), 142.0, 141.4, 134.3, 132.2, 132.1, 131.3, 130.2, 129.9, 128.2, 127.5, 127.1, 125.3, 126.3,120.7, 113.1, 111.3, 75.1 (C_3), 73.0 (C_2), 63.8 (C_5), 60.3 (C_4); Anal. Calcd for $C_{25}H_{18}BrFN_4O_3$: C, 57.60; H, 3.48; N, 10.75%. Found: C, 57.61; H, 3.47; N, 10.74%.

(2′R*,4S*,4′S*,5′R*)-4'-(2-hydroxyphenyl)-5'-phenyldispiro[imidazolidine-4,3'-pyrrolidine-2',3''-indoline]-2,2'',5-trione (4l)

White solid; Yield: (400 mg, 91%); mp (°C ± 2) = 223 °C; ^{1}H NMR (300 MHz, DMSO) δ 10.31 (s, 2H, NH), 9.29 (s, 1H, $NH_{1''}$), 7.72 (d, *J* = 4.8 Hz, 1H, Ar), 7.52 (dd, *J* = 6.6, 1.7 Hz, 3H, Ar), 7.35 – 7.15 (m, 4H, Ar), 7.02 (ddd, *J* = 23.5, 11.2, 4.4 Hz, 2H, Ar), 6.90 – 6.65 (m, 3H, Ar), 6.14 (s, 1H, Ar), 5.70 (dd, *J* = 9.5, 5.9 Hz, 1H, H_5), 4.30 (d, *J* = 9.5 Hz, 1H, H_4), 3.77 (d, *J* = 5.8 Hz, 1H, H_1); ^{13}C NMR (75 MHz, DMSO) δ 178.9 ($C_{2'}$), 176.1 ($C_{5''}$), 156.2, 155.9 ($C_{2''}$), 143.2, 142.8, 129.4, 128.0, 127.7, 127.3, 127.1, 127.0, 126.8, 121.4, 119.0, 114.5, 109.3, 73.8 (C_3), 73.4 (C_2), 64.4 (C_5), 61.2 (C_4); Anal. Calcd for $C_{25}H_{20}N_4O_4$: C, 68.17; H, 4.58; N, 12.72%. Found: C, 68.15; H, 4.57; N, 12.74%.

(2′R*,4S*,4′S*,5′R*)-5''-chloro-4'-(2-hydroxyphenyl)-5'-phenyldispiro[imidazolidine-4,3'-pyrrolidine-2',3''-indoline]-2,2'',5-trione (4m)

White solid; Yield: (417 mg, 88%); mp (°C ± 2) = 223 °C; ^{1}H NMR (300 MHz, DMSO) δ 10.46 (s, 2H, NH), 9.35 (s, 1H, $NH_{1''}$), 7.70 (d, J = 2.7 Hz, 1H, Ar), 7.51 (t, J = 5.1 Hz, 3H, Ar), 7.32 – 7.18 (m, 4H, Ar), 7.04 (t, J = 7.1 Hz, 1H, Ar), 6.87 (d, J = 6.4 Hz, 1H, Ar), 6.80 (d, J = 8.3 Hz, 1H, Ar), 6.69 (d, J = 10.2 Hz, 1H, Ar), 6.41 (s, 1H, Ar), 5.68 (dd, J = 9.1, 4.9 Hz, 1H, H_5), 4.23 (d, J = 9.6 Hz, 1H, H_4), 4.02 (d, J = 5.1 Hz, 1H, H_1); ^{13}C NMR (75 MHz, DMSO) δ 178.8 ($C_{2'}$), 175. 5 ($C_{5''}$), 156.2, 155.8 ($C_{2''}$), 142.8, 142.2, 129.4, 129.2, 128.0, 127.7, 127.4, 127.2, 126.9, 125.4, 122.0, 119.3, 118.9, 114.4, 110.6, 75.3 (C_3), 73.2 (C_2), 64.1 (C_5), 61.0 (C_4); Anal. Calcd for $C_{25}H_{19}ClN_4O_4$: C, 63.23; H, 4.03; N, 11.80%. Found: C, 63.24; H, 4.05; N, 11.81%.

2. Copies of ^{1}H- and ^{13}C-NMR spectra of compounds 4a-m

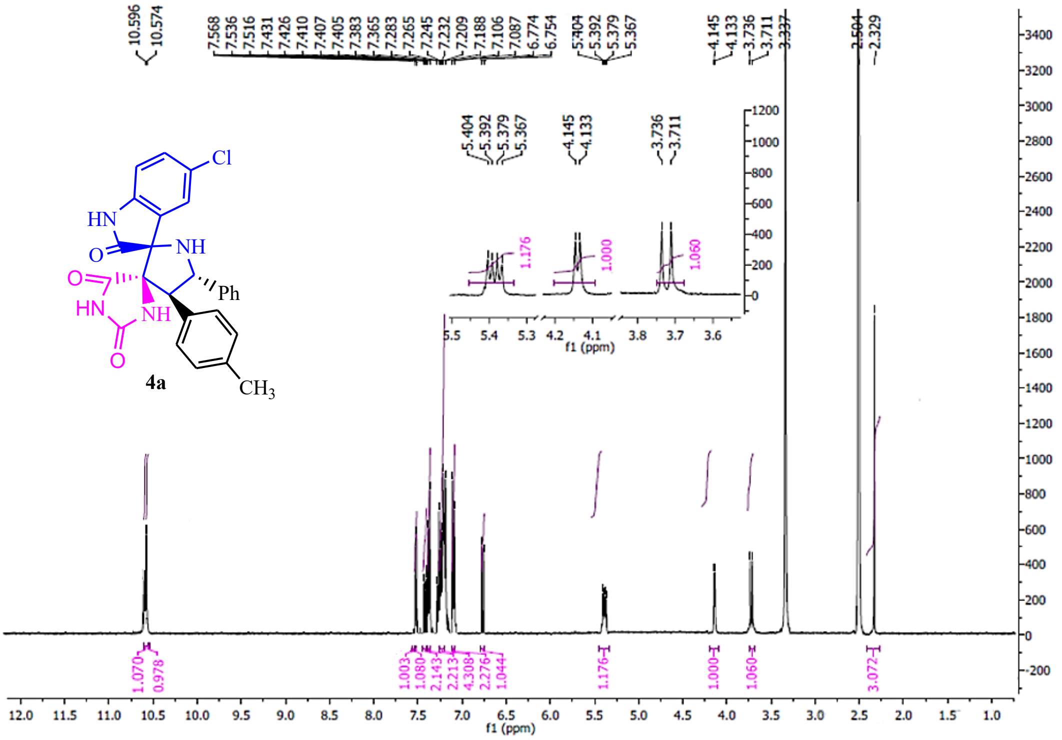

Figure S1. ^{1}H NMR spectrum of compound **4a** (400 MHz, DMSO-d_6).

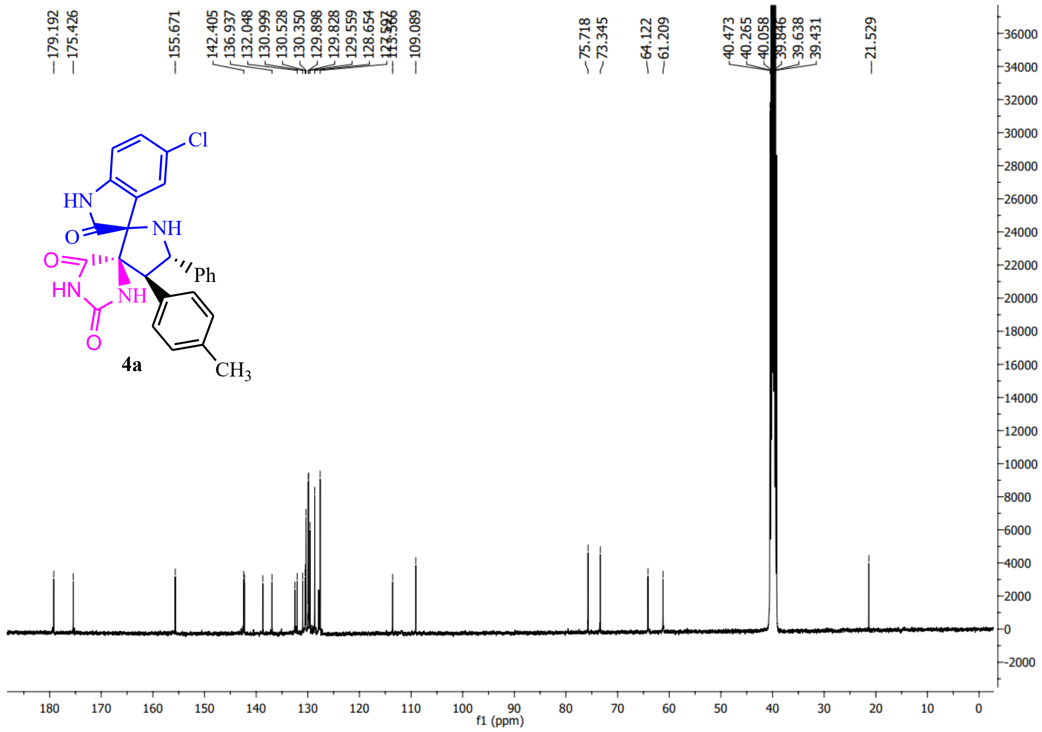

Figure S2. ^{13}C {^{1}H} NMR spectrum of compound **4a** (101 MHz, DMSO-d_6).

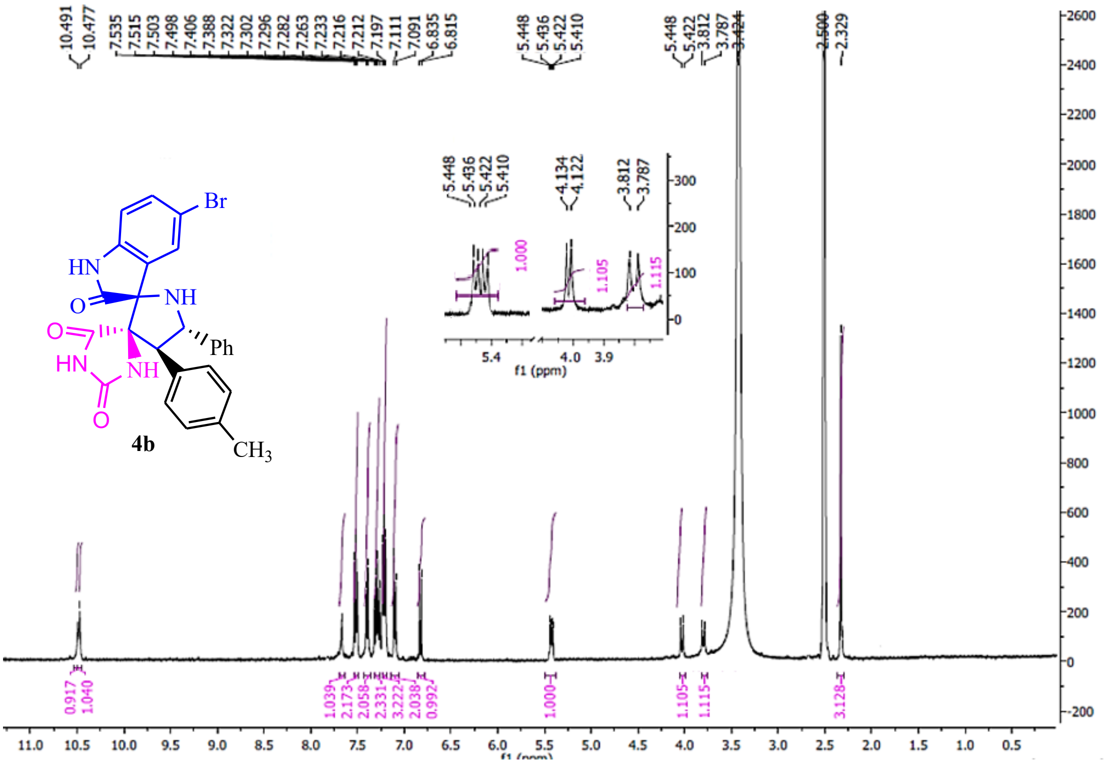

Figure S3. ^{1}H NMR spectrum of compound **4b** (400 MHz, DMSO-*d*6).

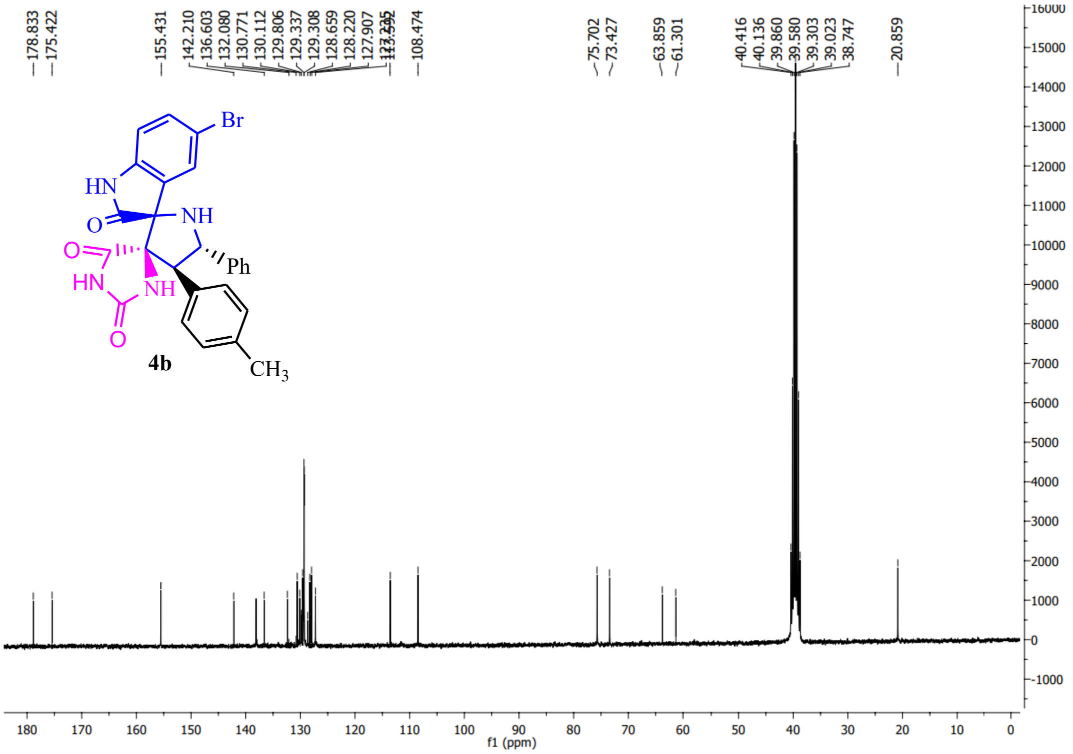

Figure S4. ^{13}C {^{1}H} NMR spectrum of compound **4b** (101 MHz, DMSO-*d*6).

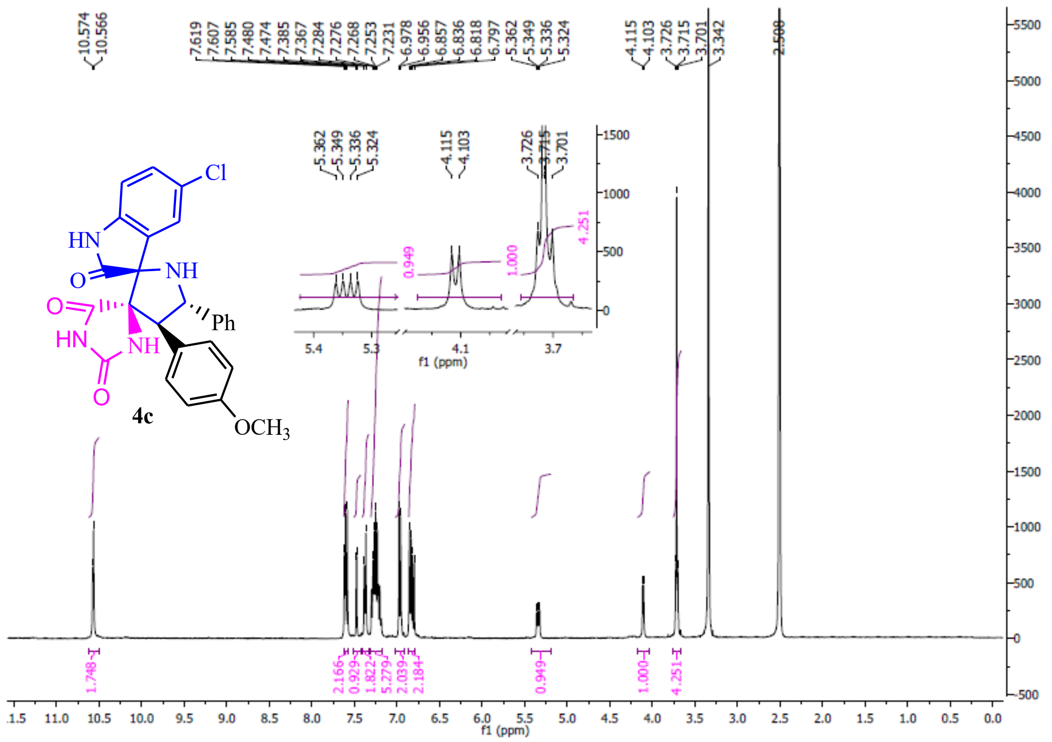

Figure S5. ^{1}H NMR spectrum of compound **4c** (400 MHz, DMSO-d_6).

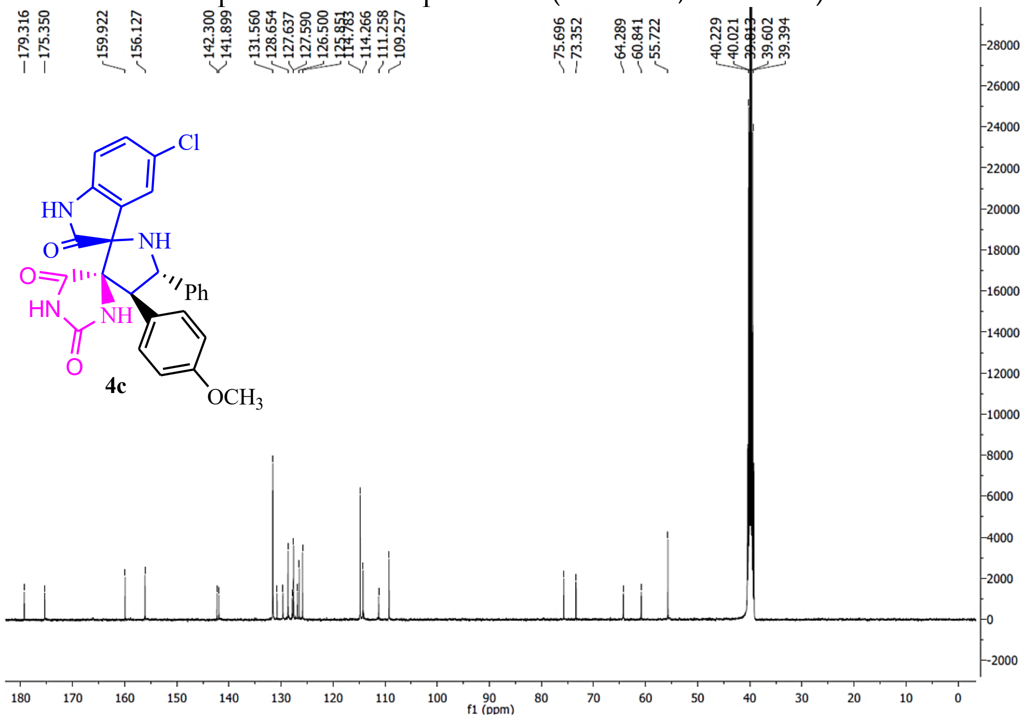

Figure S6. ^{13}C {^{1}H} NMR spectrum of compound **4c** (101 MHz, DMSO-d_6).

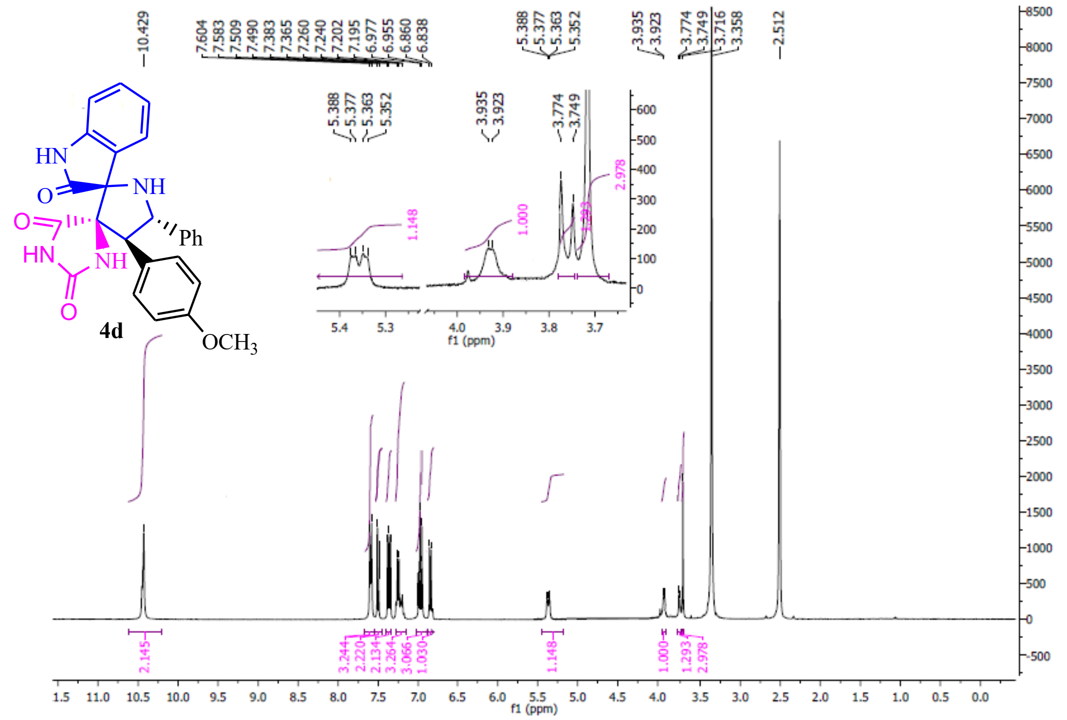

Figure S7. ^{1}H NMR spectrum of compound **4d** (400 MHz, DMSO-*d*6).

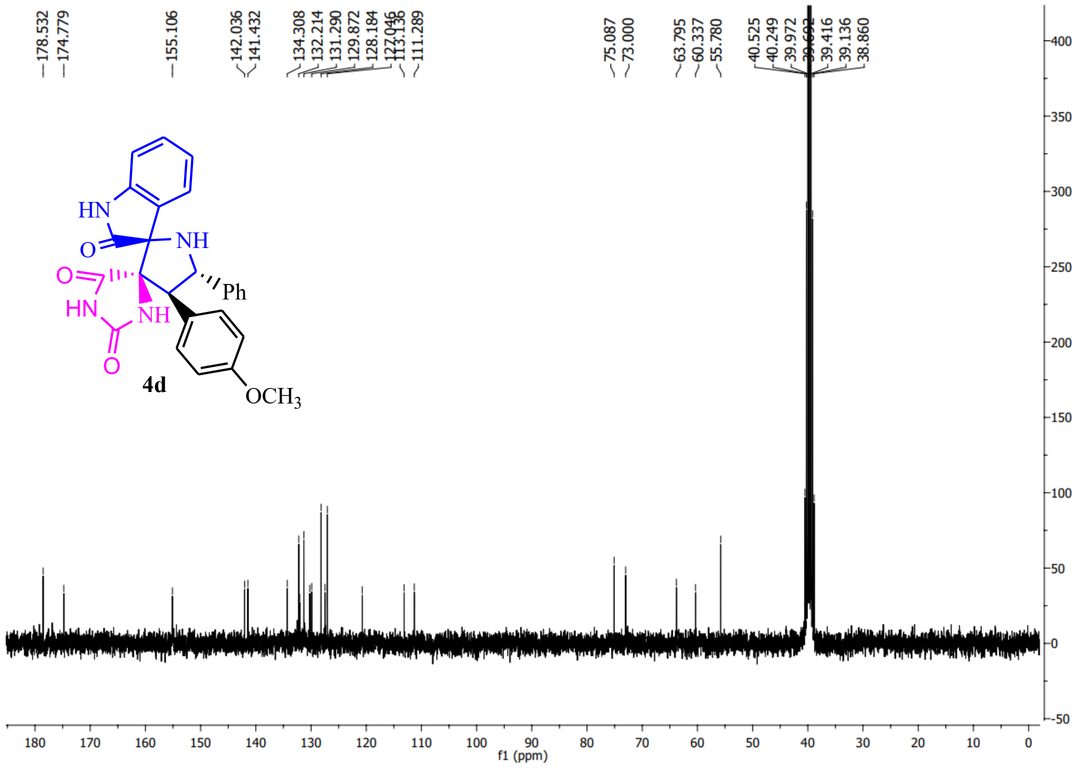

Figure S8. ^{13}C {^{1}H} NMR spectrum of compound **4d** (101 MHz, DMSO-*d*6).

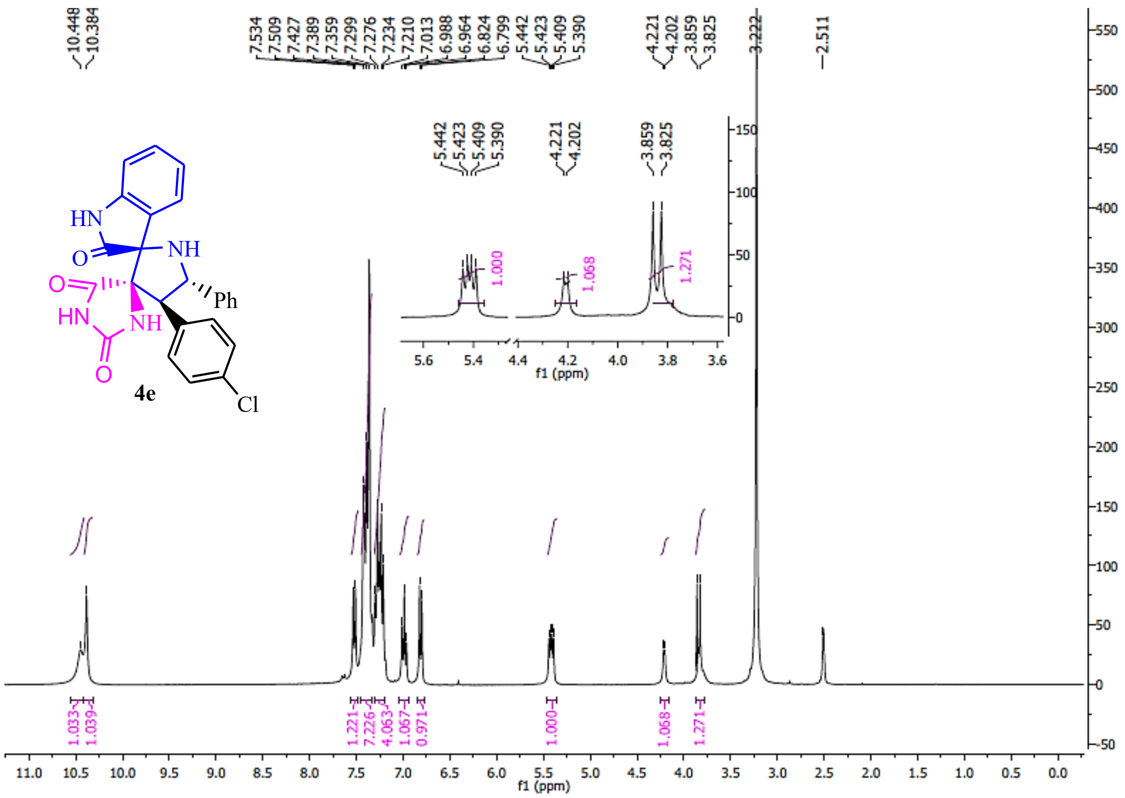

Figure S9. ^{1}H NMR spectrum of compound **4e** (300 MHz, DMSO-d_6).

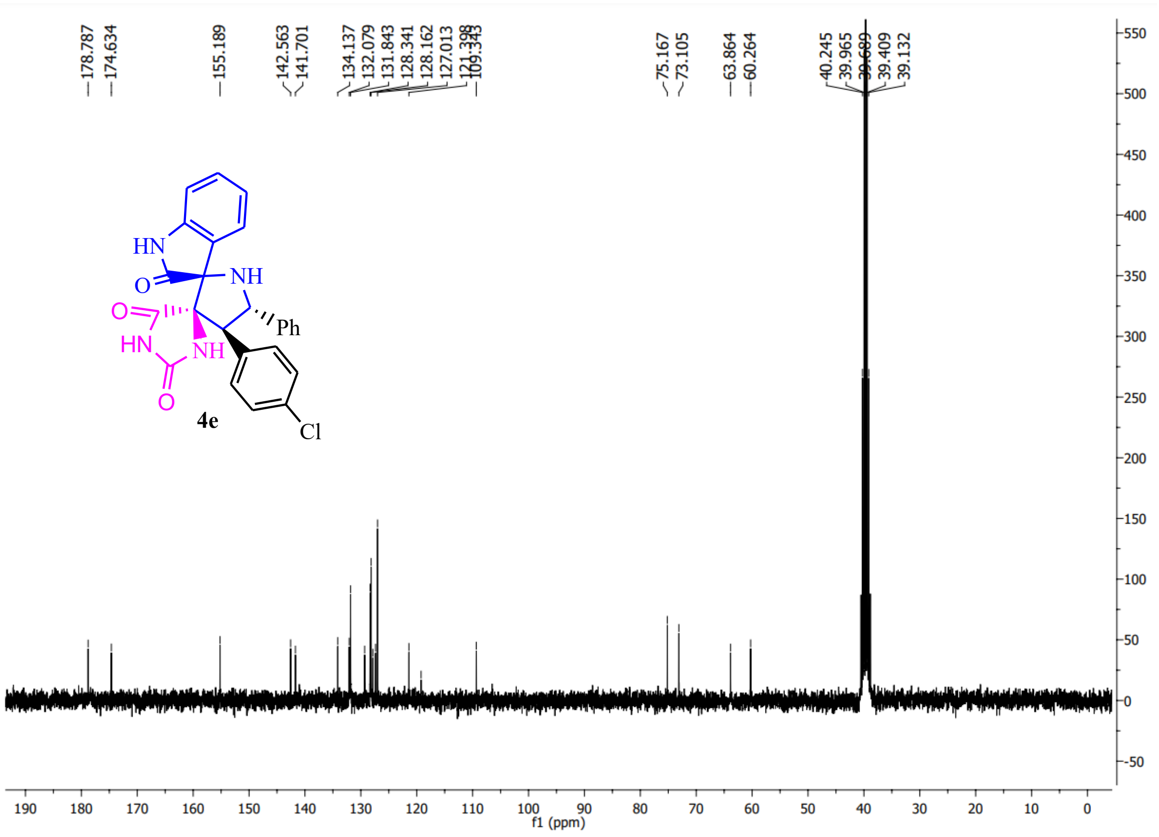

Figure S10. ^{13}C {^{1}H} NMR spectrum of compound **4e** (75 MHz, DMSO-d_6).

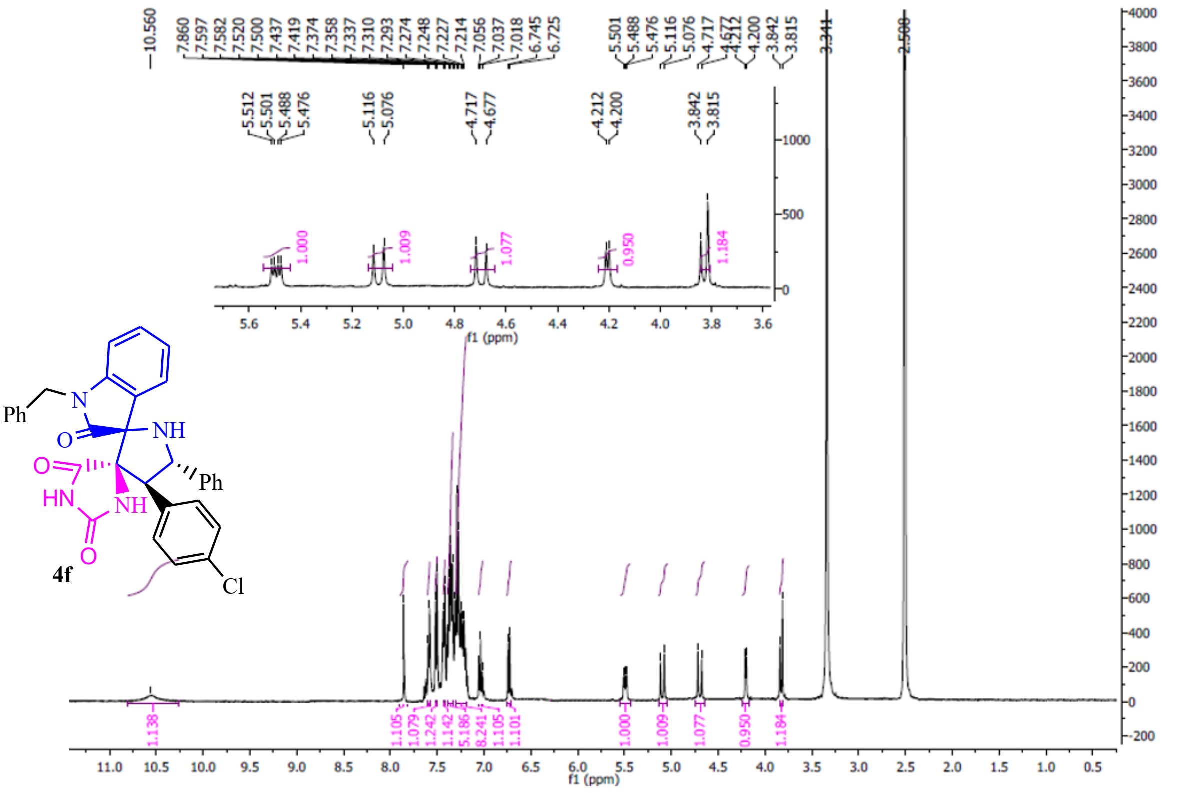

Figure S11. ^{1}H NMR spectrum of compound **4f** (400 MHz, DMSO-d_6).

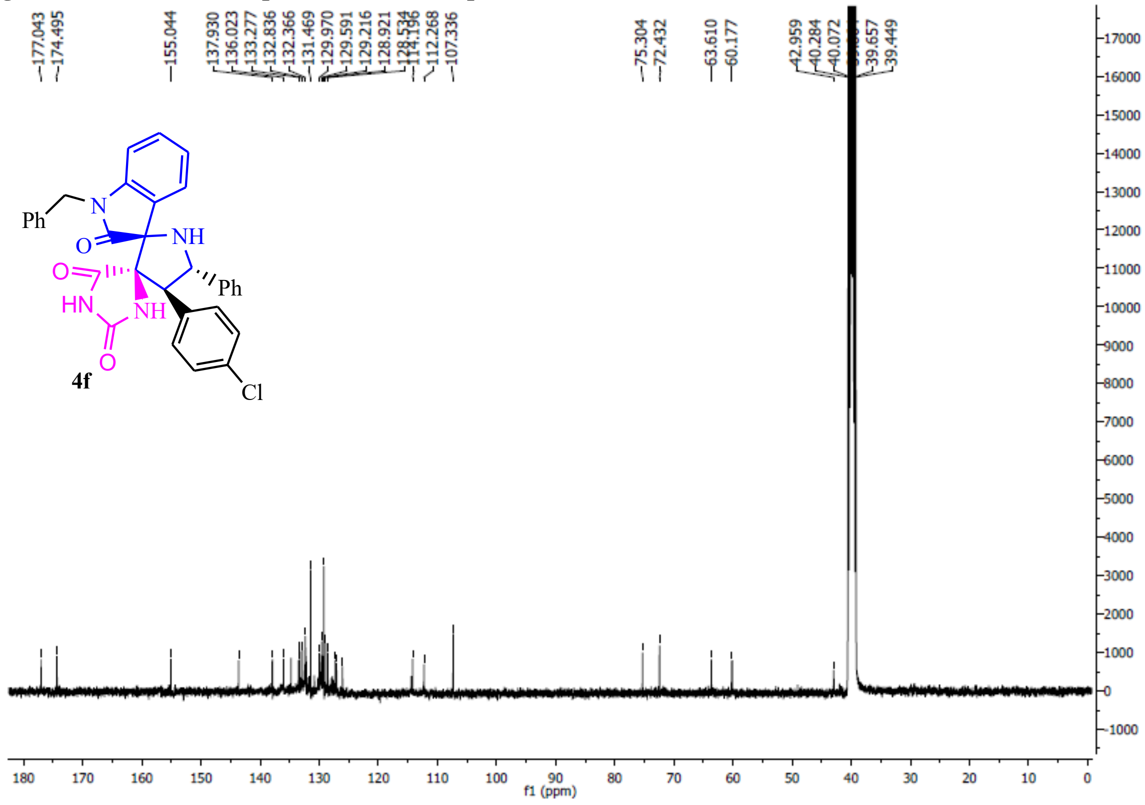

Figure S12. ^{13}C {^{1}H} NMR spectrum of compound **4f** (101 MHz, DMSO-d_6).

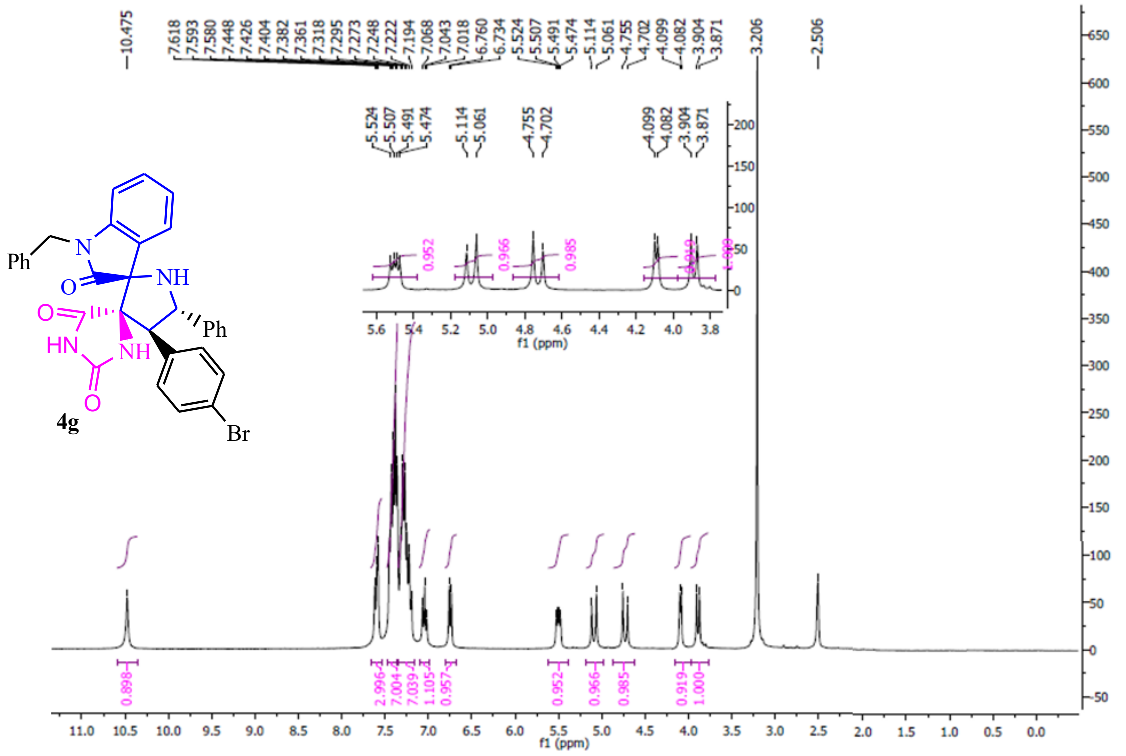

Figure S13. ^{1}H NMR spectrum of compound **4g** (300 MHz, DMSO-*d*6).

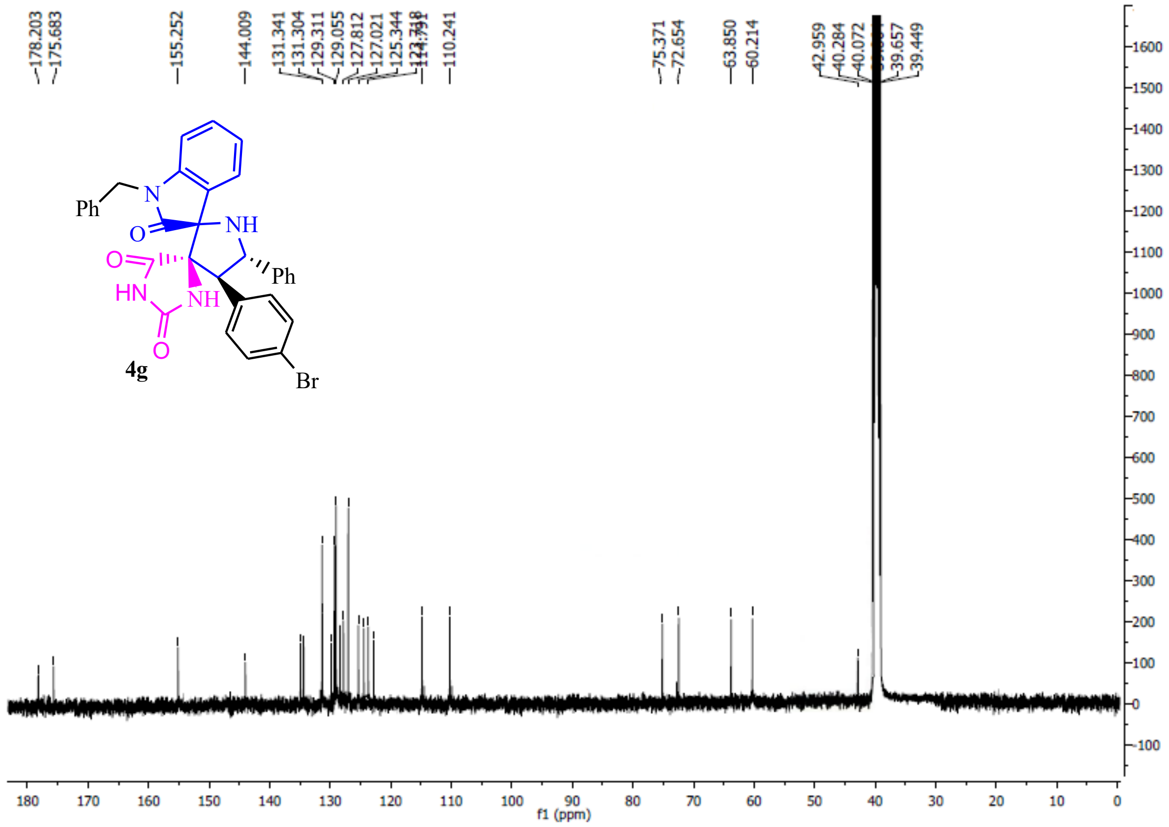

Figure S14. ^{13}C {^{1}H} NMR spectrum of compound **4g** (75 MHz, DMSO-*d*6).

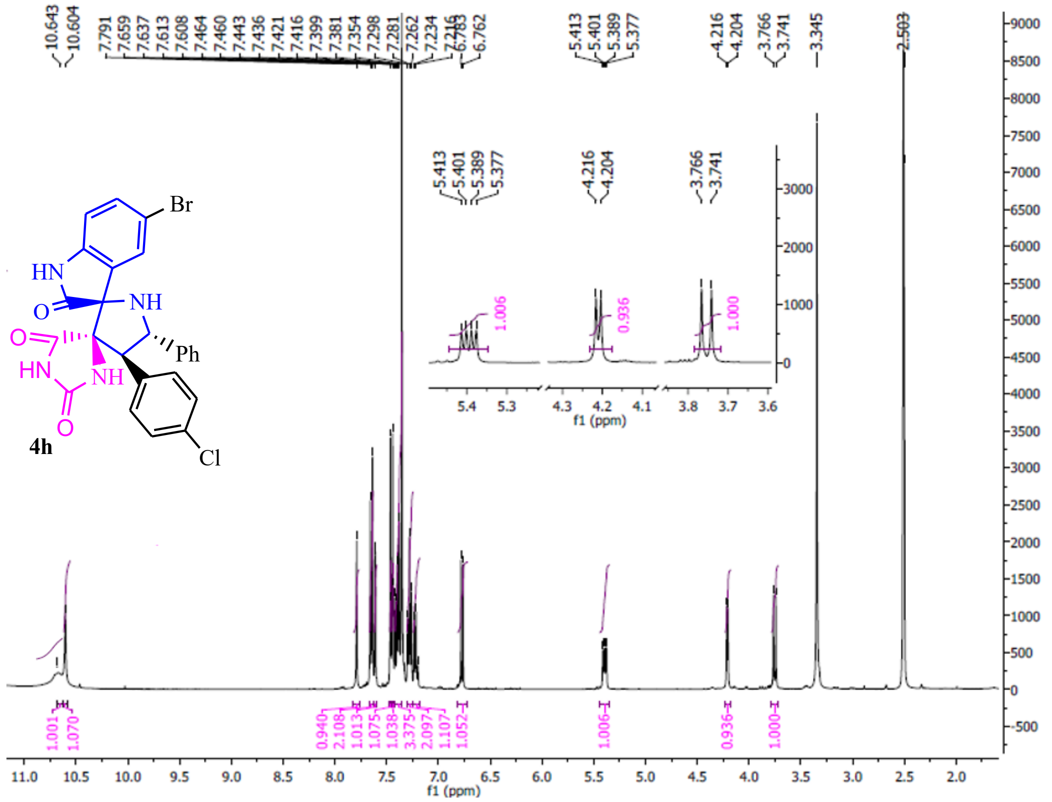

Figure S15. ^{1}H NMR spectrum of compound **4h** (400 MHz, DMSO-d_6).

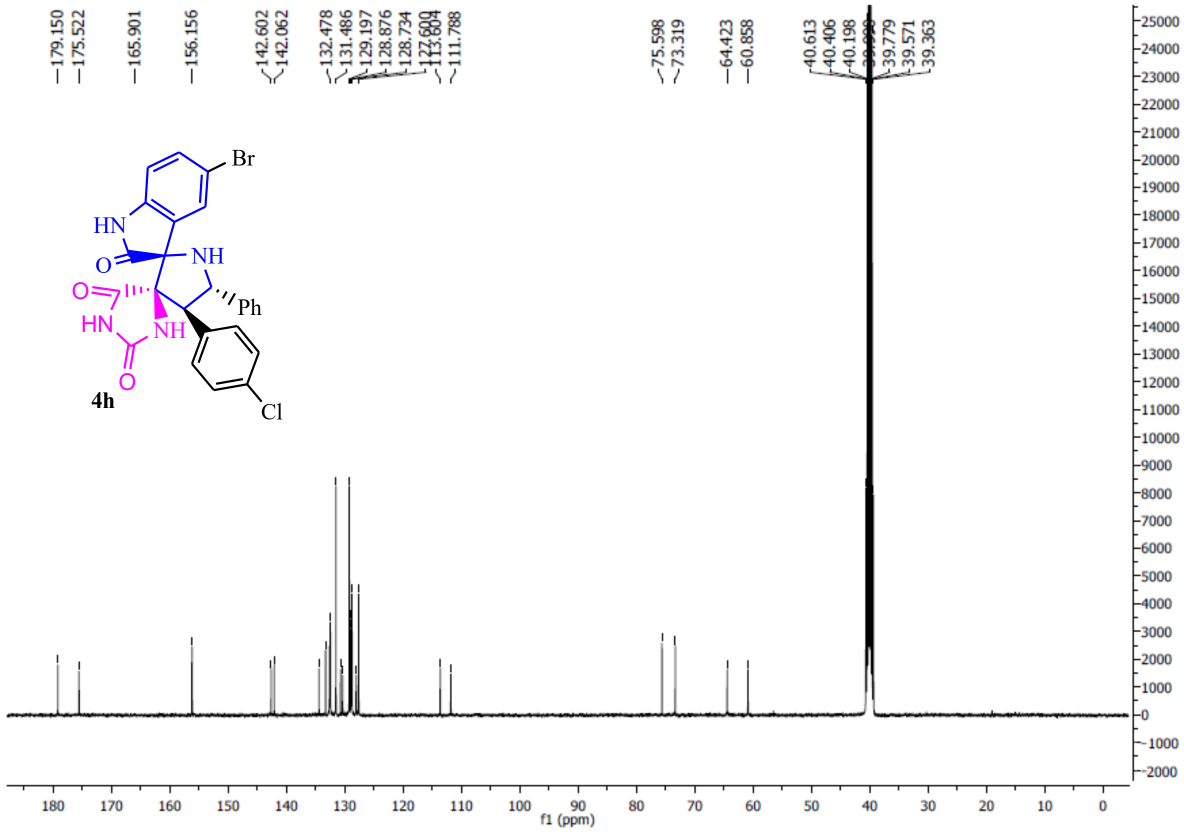

Figure S16. ^{13}C {^{1}H} NMR spectrum of compound **4h** (101 MHz, DMSO-d_6).

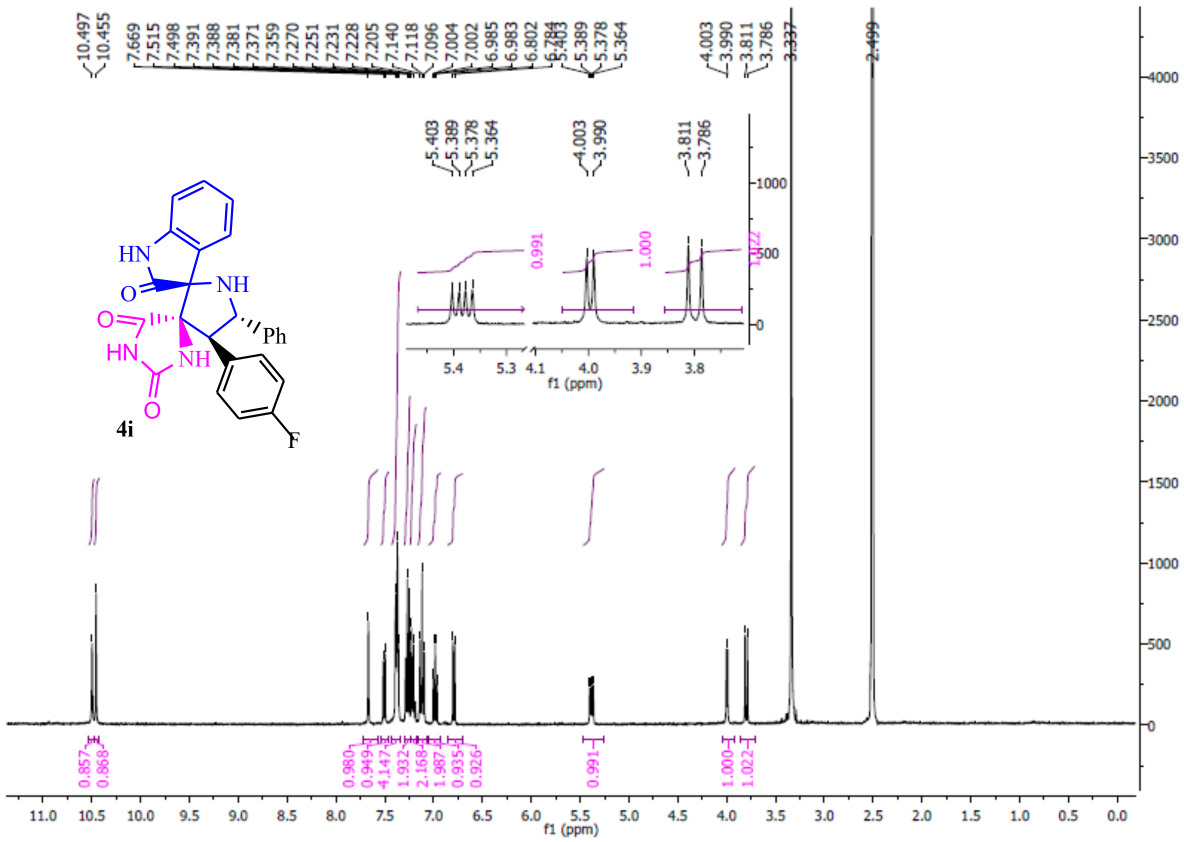

Figure S17. ^{1}H NMR spectrum of compound **4i** (400 MHz, DMSO-d_6).

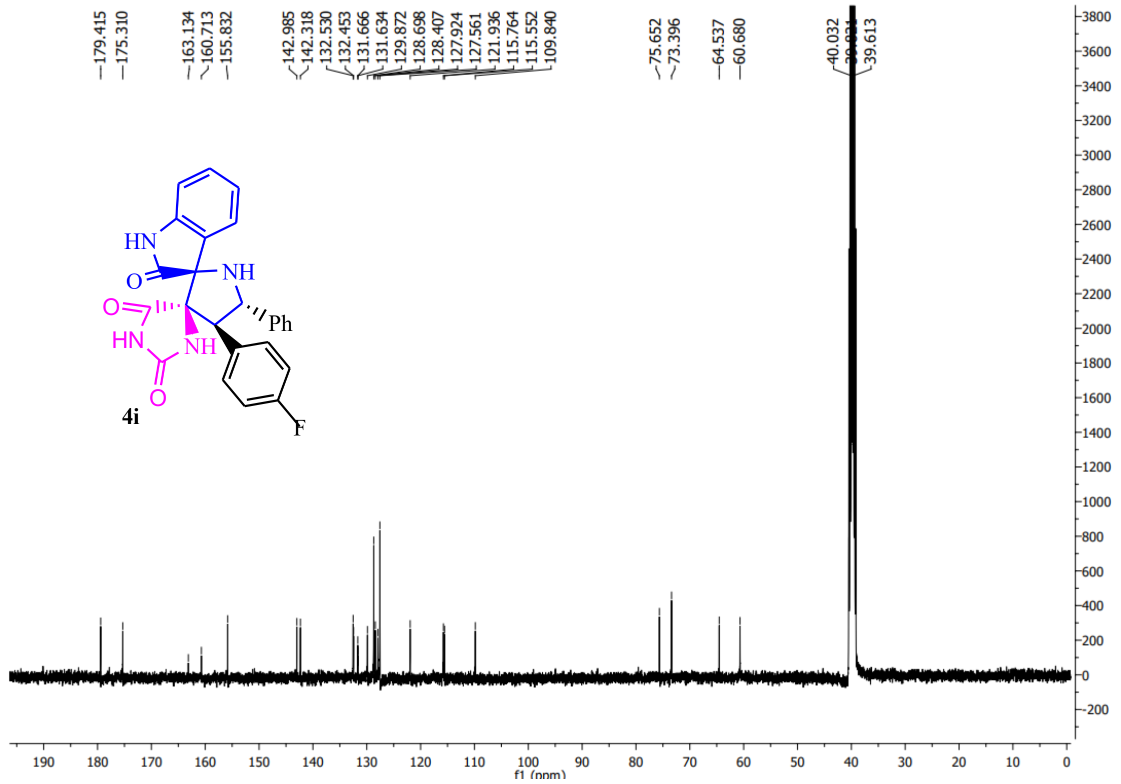

Figure S18. ^{13}C {^{1}H} NMR spectrum of compound **4i** (101 MHz, DMSO-d_6).

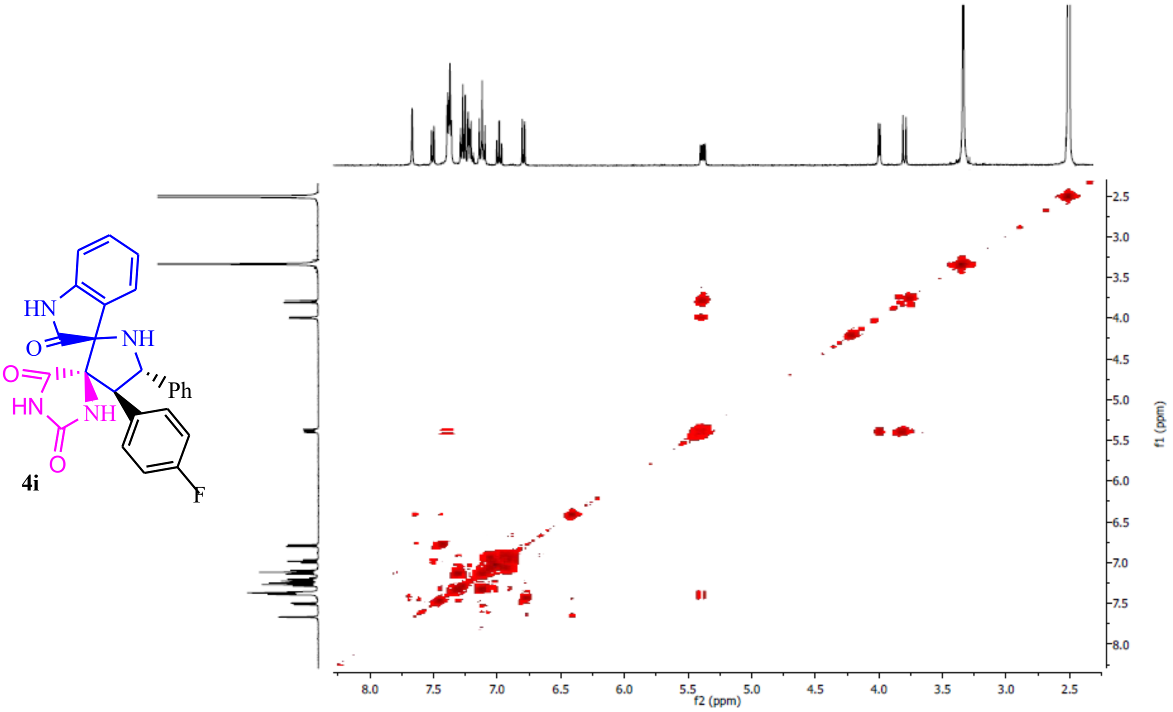

Figure S19. ¹H-¹H COSY spectrum of compound **4i**.

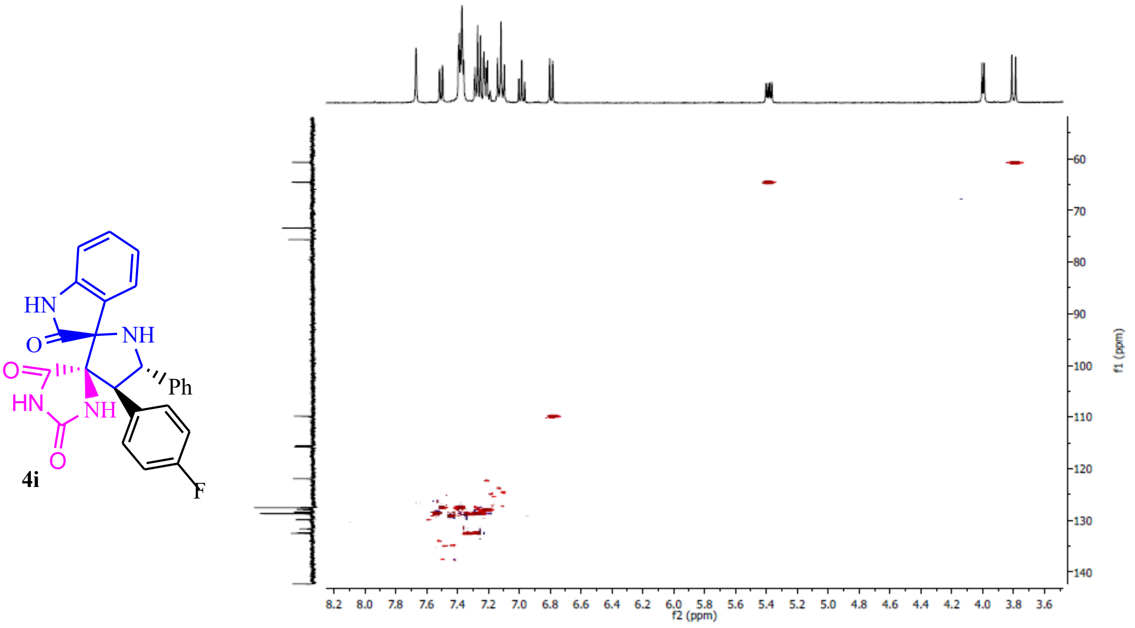

Figure S20. ¹H-¹³C correlations in the HSQC spectrum of compound **4i**.

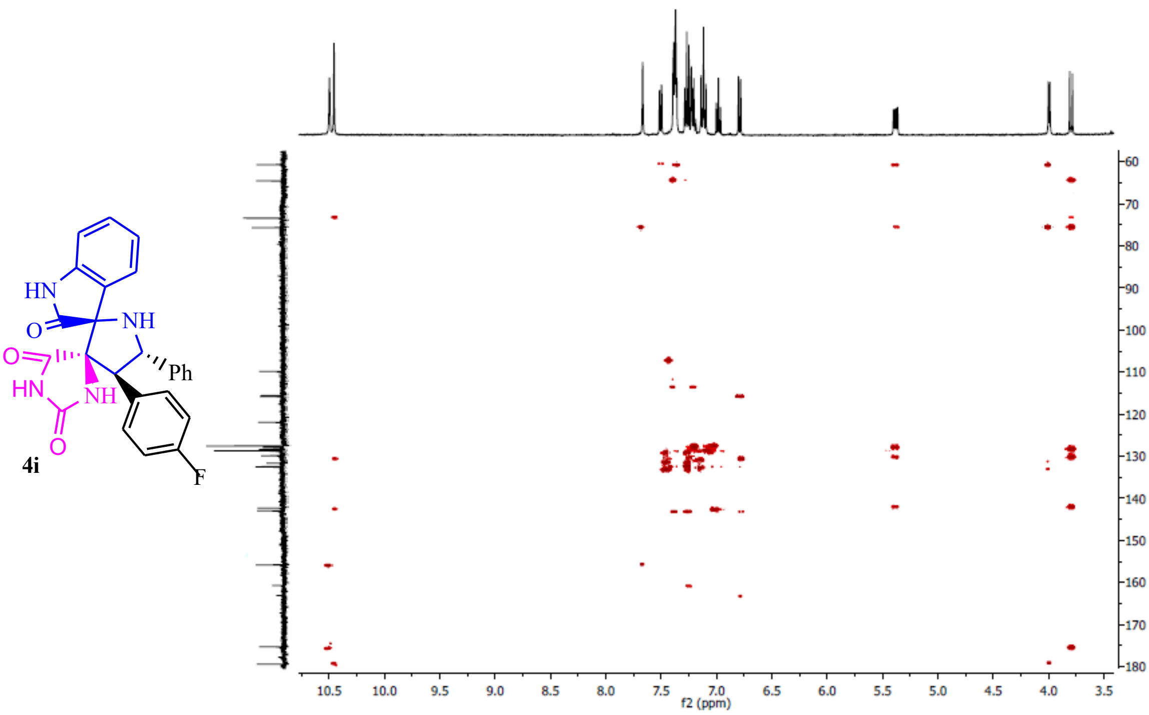

Figure S21. ^{1}H-^{13}C correlations in the HMBC spectrum of compound **4i**.

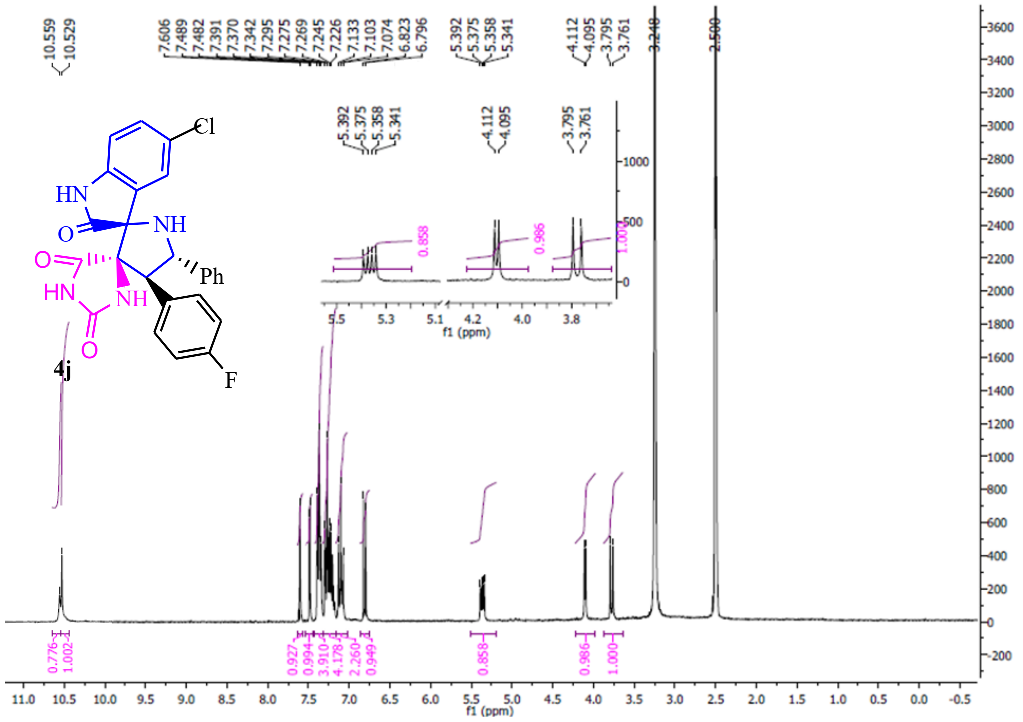

Figure S22. ^{1}H NMR spectrum of compound **4j** (300 MHz, DMSO-d_6).

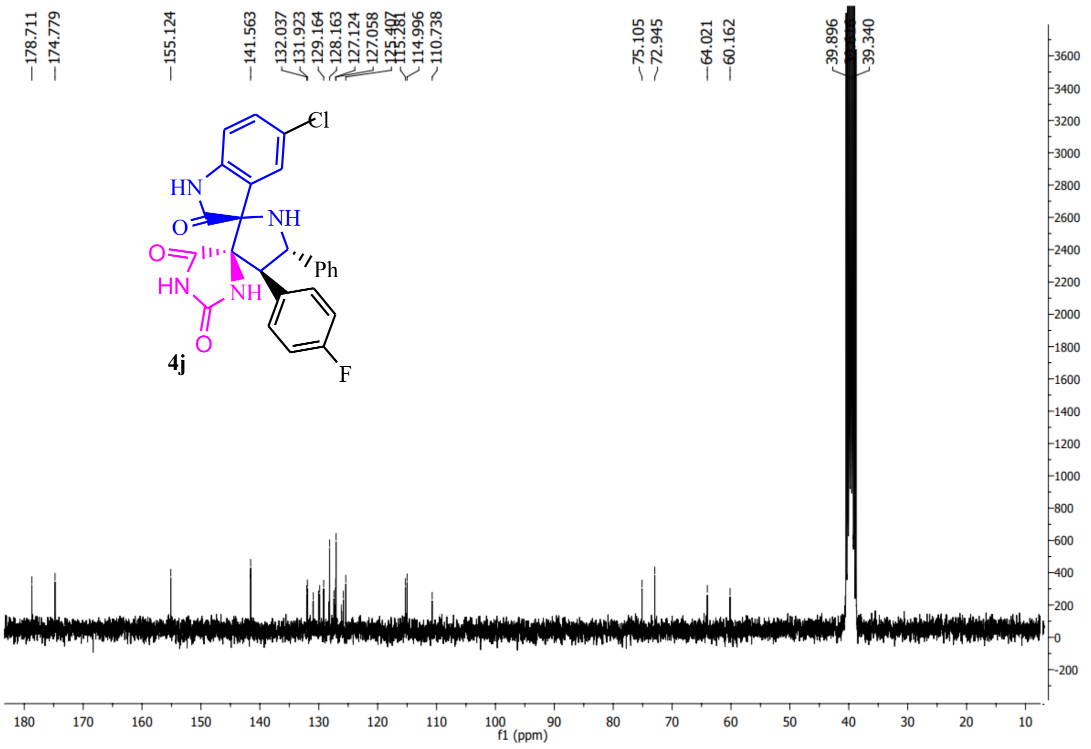

Figure S23. ^{13}C {^{1}H} NMR spectrum of compound **4j** (75 MHz, DMSO-d_6).

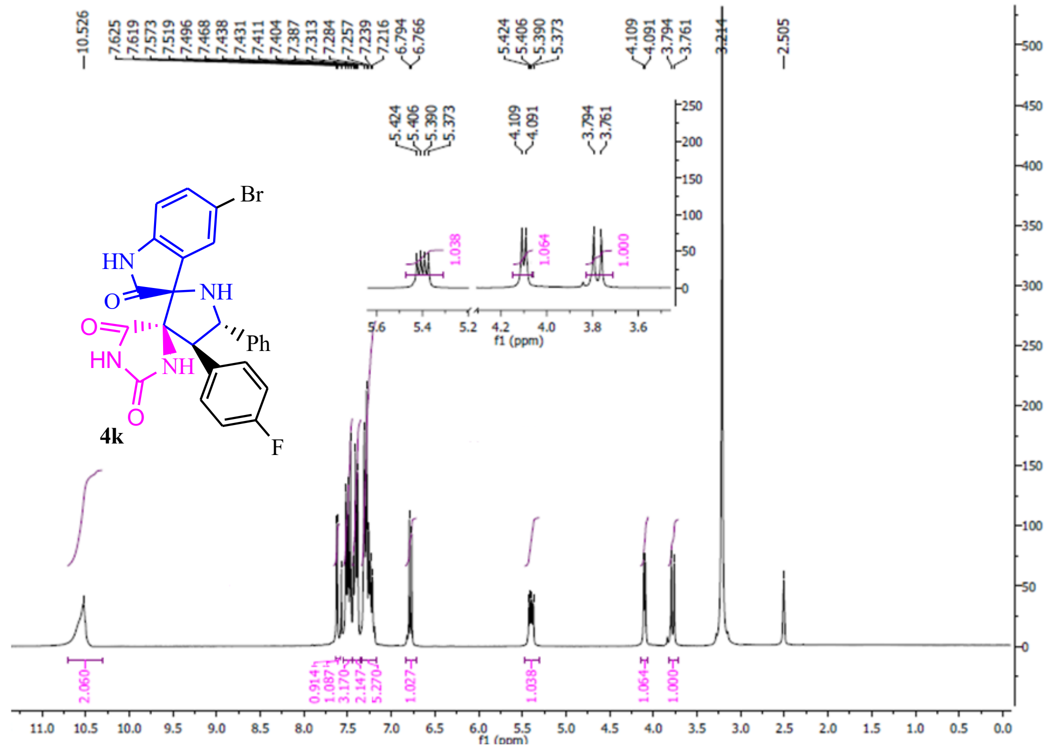

Figure S24. ^{1}H NMR spectrum of compound **4k** (300 MHz, DMSO-d_6).

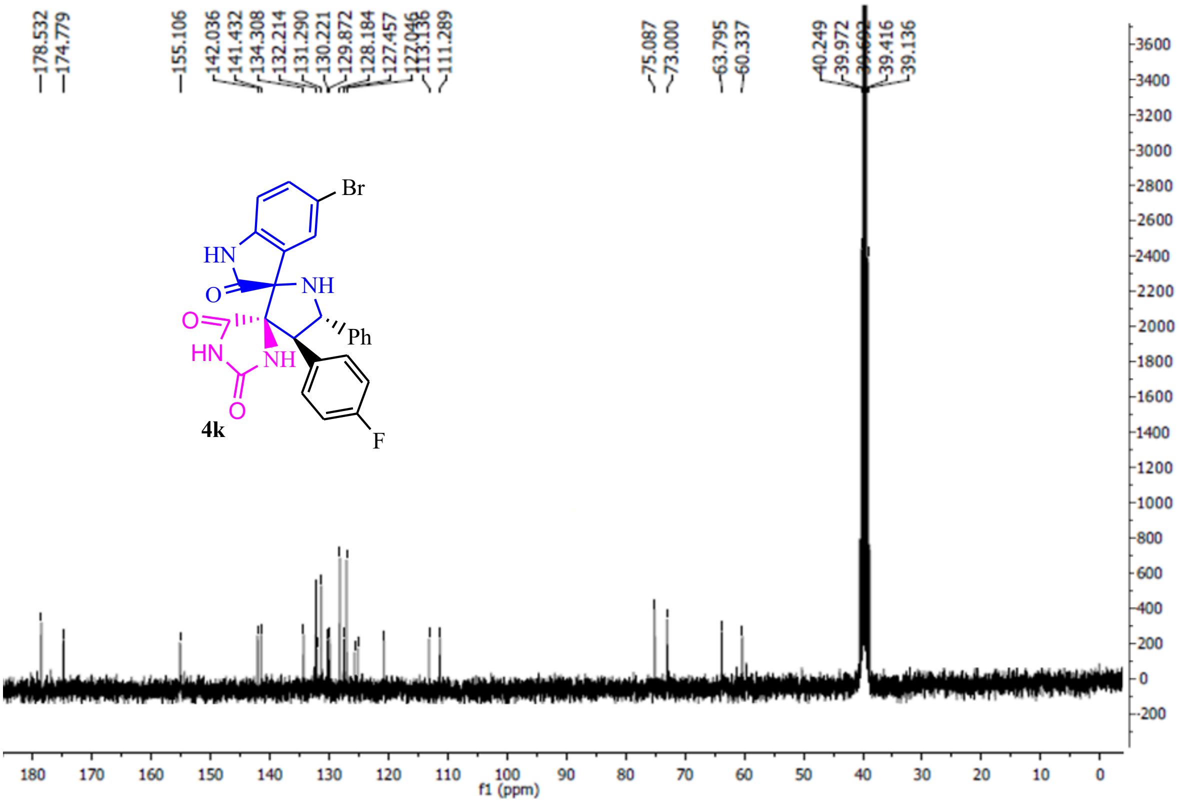

Figure S25. $^{13}C\{^{1}H\}$ NMR spectrum of compound **4k** (75 MHz, DMSO-d_6).

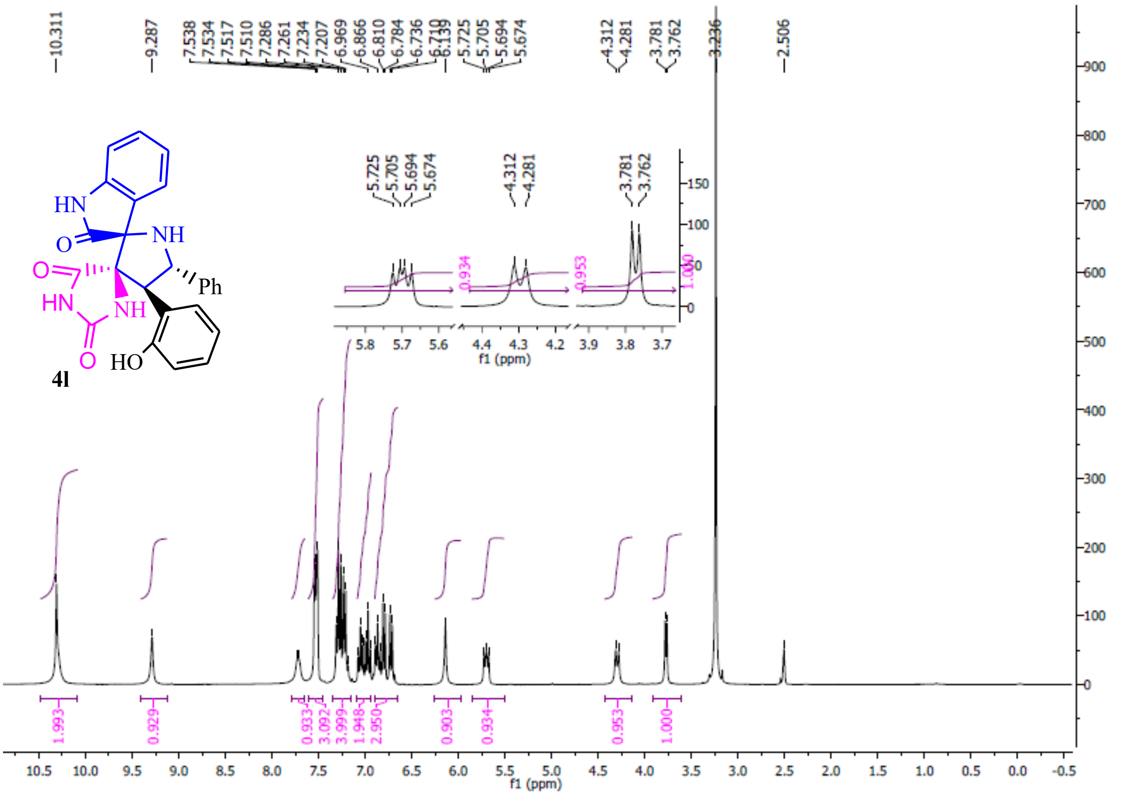

Figure S26. ^{1}H NMR spectrum of compound **4l** (300 MHz, DMSO-d_6).

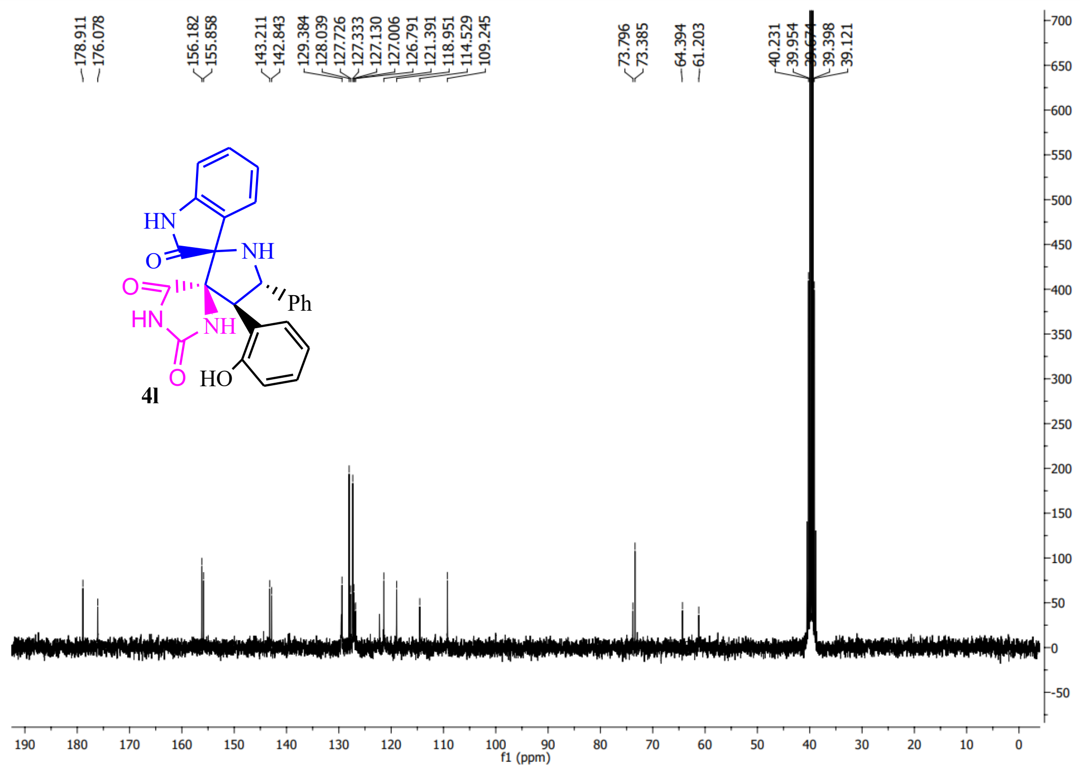

Figure S27. $^{13}C\ \{^{1}H\}$ NMR spectrum of compound **4l** (75 MHz, DMSO-*d*6).

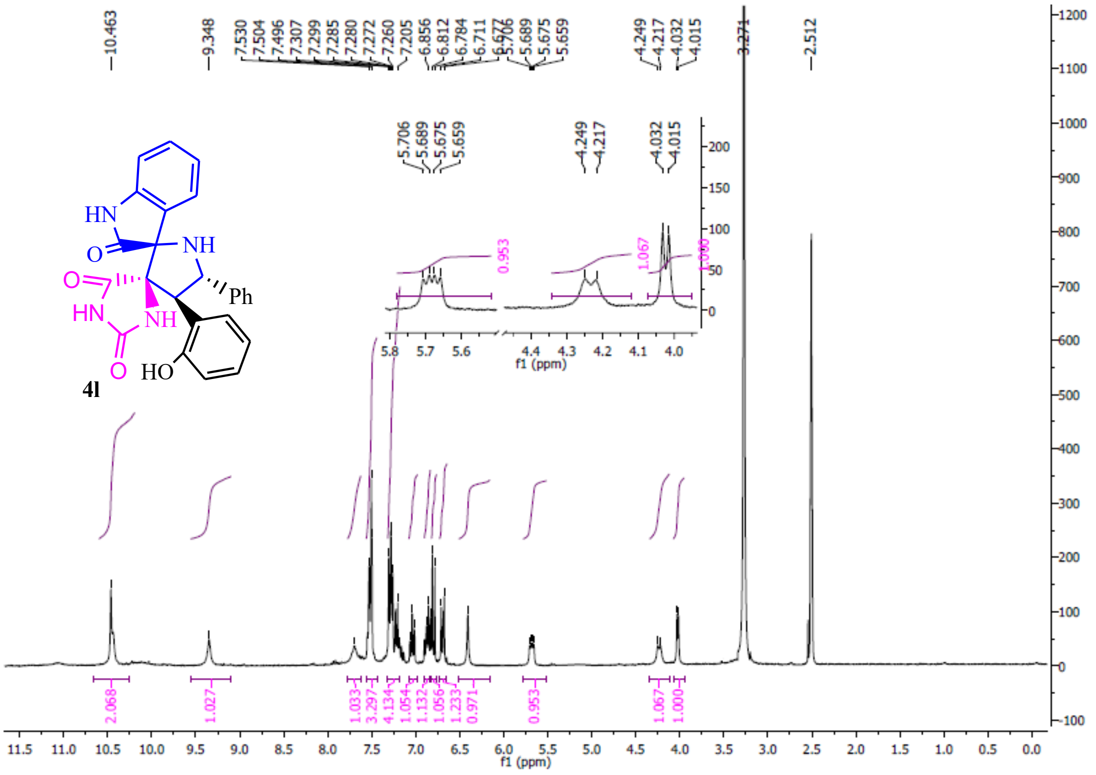

Figure S28. ^{1}H NMR spectrum of compound **4m** (300 MHz, DMSO-*d*6).

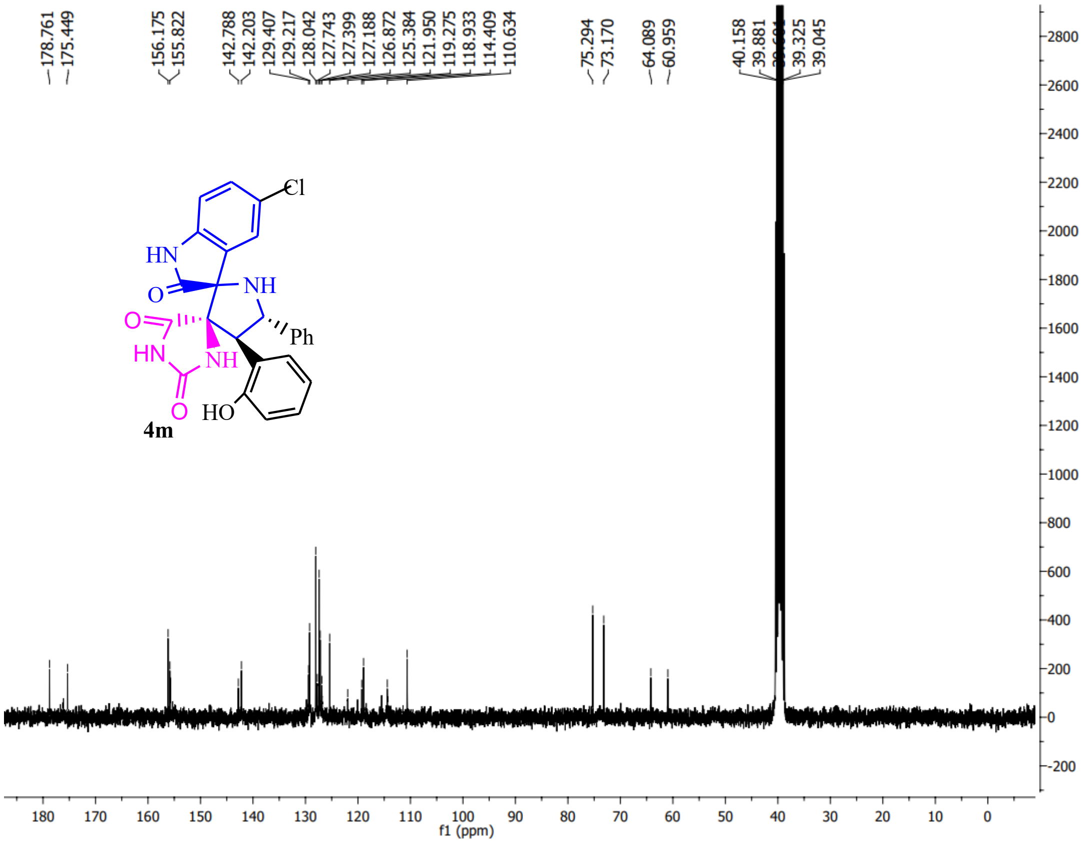

3. **Figure S29.** ^{13}C {^{1}H} NMR spectrum of compound **4m** (75 MHz, DMSO-d_6).

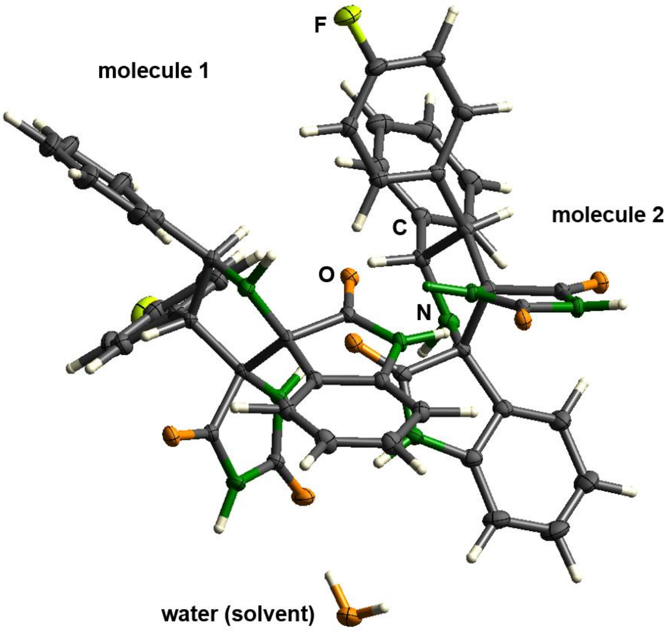

Figure S30. View of the asymmetric unit of **4i** containing two independent molecules and co-crystallized H_2O.

4. Molecular docking study

4.1. **Table S1**. Information related to the studied enzyme in the docking studies

Targets PDB	Methods	Organism	Chain	Sequence length	Resolution (Å)	Native-ligand
**4QGG**	X-ray diffraction	*Staphylococcus aureus*	A,B	205	1.62	32C
**4EWP**	X-ray diffraction	*Micrococcus luteus NCTC 2665*	A,B,C,D,E,F	350	2.20	/
**4DUH**	X-ray diffraction	*Escherichia coli K-12*	A,B	220	1.50	RLI
**3Q70**	X-ray diffraction	*Candida albicans*	A	342	1.40	RIT
**3V99**	X-ray diffraction	*Homo sapiens*	A,B	691	2.20	ACD

The native ligand of 4QGG is 32C: **(2-(3-chlorophenoxy)-3-fluoro-4-{(1R)-3-methyl-1-[(3S)-3-(5-methyl-2,4-dioxo-3,4-dihydropyrimidin-1(2H)-yl)piperidin-1-yl]butyl}benzoic acid** ($C_{28}H_{31}ClFN_3O_5$). The native ligand of 4DUH is RLI : **4-{[4'-methyl-2'-(propanoylamino)-4,5'-bi-1,3-thiazol-2-yl]amino}benzoic acid** ($C_{17}H_{16}N_4O_3S_2$). The native ligand of 3Q70 is: RIT : (**Ritonavir** ($C_{37}H_{48}N_6O_5S_2$). The native ligand of 3V99 is: ACD (**Acdarachidonic Acid** ($C_{20}H_{32}O_2$).

4.2. Molecular docking study of the antibacterial and antifungal targets

The results obtained after the docking calculations and the best pose received for the compounds **4c** and **4e** with the bacterial (***P. aeruginosa*** **(PDB ID: 4JVI))** and fungal **(*C .krusei* (PDB ID: 4O92))** targets have been grouped in the Table S2 and S3 respectively.

Table S2. MolDock Score and interactions between **4c** with antibacterial target.

Antibacterial targets							
P. aeruginosa **(PDB ID: 4JVI)**							
		**Bonds between atoms of compounds and active site residues**					
**Comp.**	**MolDock Score (kcal/mol)**	**Atom of compound**	**Involved receptor atoms**	**Involved receptor residues**	**Category**	**Type**	**Distance (Å)**
**4c**	-119.407	H1	O	LEU207	H-Bond	Conventional H-Bond	2.18
		Cl1	/	LEU207	Hydrophobic	Alkyl	4.11
		6-ring	/	ILE236	Hydrophobic	Pi-Alkyl	3.99
		6-ring	/	VAL211	Hydrophobic	Pi-Alkyl	4.97
		6-ring	/	ILE236	Hydrophobic	Pi-Alkyl	5.23
**Tetracycline**	-82.805	H23	O	ILE149	H-Bond	Conventional H-Bond	2.68
		O3	HD1	PRO238	H-Bond	Carbon H-Bond	3.01
		/	HB2	LEU208	Hydrophobic	Pi-Sigma	2.34
		C3	/	LEU208	Hydrophobic	Alkyl	5.23
		C3	/	ILE236	Hydrophobic	Alkyl	3.38
		6-ring	/	ALA130	Hydrophobic	Pi-Alkyl	4.70
		6-ring	/	LEU197	Hydrophobic	Pi-Alkyl	4.18

a. Orientation and bonding interaction compound 4c in the active site cavity of Pseudomonas aeruginosa quorum sensing regulator PqsR (PDB: 4JVI):

The obtained results revealed that the compound **4c** bind into the PqsR target. The MolDock Score energy and types of possible interactions of our docked compound is represented in Table S2 and Figure 1.

The molecular docking studies revealed that the compound **4c** has a many binding mode with the pocket of PqsR target which shows a lowest MolDock Score: -119.407 kcal/mol compared to Tetracycline with the MolDock Score of -82.805 kcal/mol (Table S2). However the results of the docking thus indicated a slight difference in the binding of the compound **4c** to the PqsR enzyme *vs*. the Tetracycline.

Furthermore, the compound **4c** show a good affinity with the binding site of PqsR and this is confirmed by forming one strong hydrogen bonding interactions with: LEU207 (2.18Å), As shown from the obtained data compound **4c** was able to form four hydrophobic interactions with different amino acids in the PqsR binding pocket which are: LEU207, ILE236, VAL211, and ILE236. (Table S2 and Figure S31).

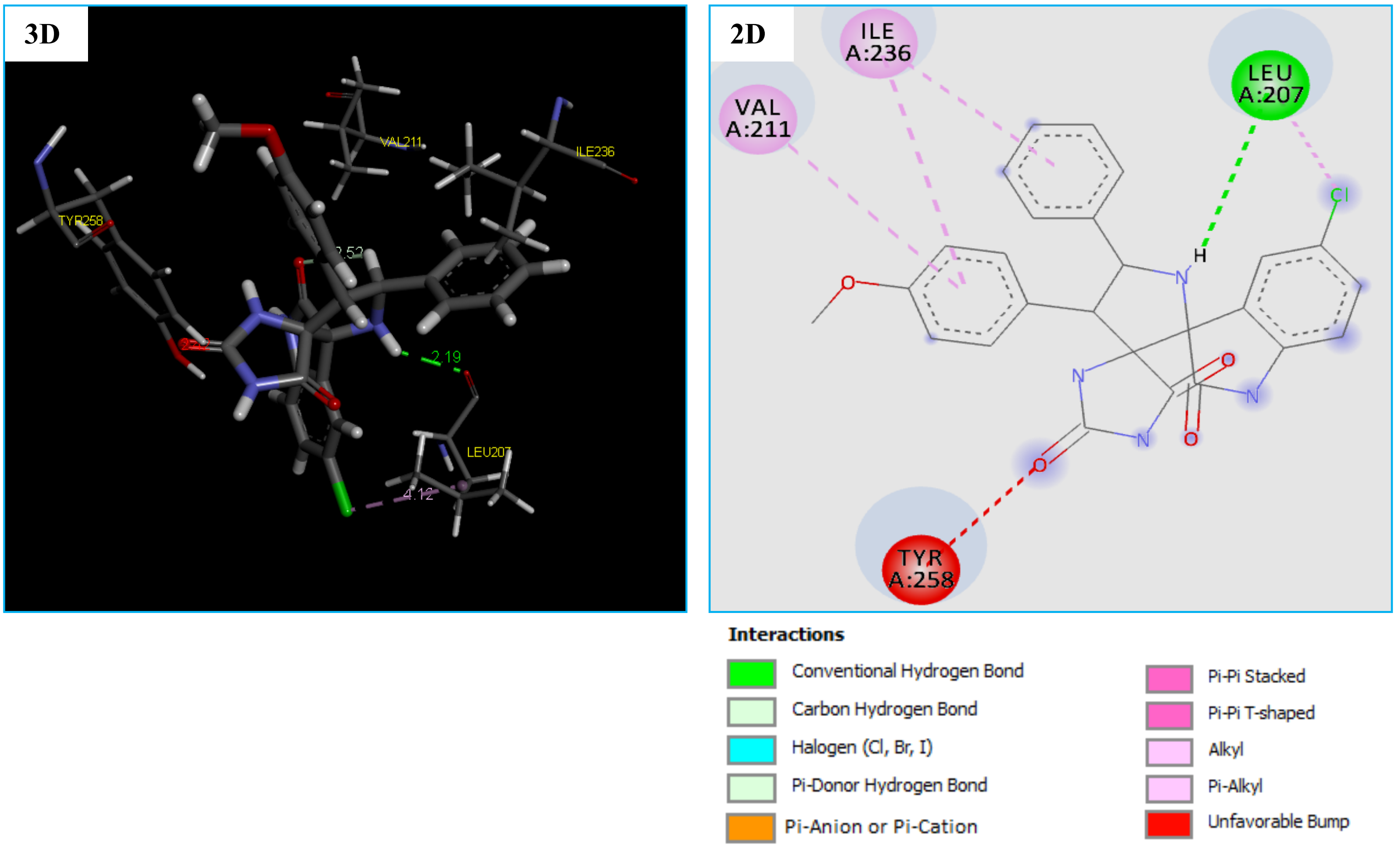

Figure S31. Representation 2D and 3D of interactions of the **4c** in the active site cavity of PqsR (PDB: **4JVI**).

Table S3. MolDock Score and interactions between the **4e** with antifungal target.

				Antifungal targets			
				C .krusei **(PDB ID: 4O92)**			
		O2	HT1	VAL106	H-Bond	Conventional H-Bond	2.04
		H7	O	HIS159	H-Bond	Conventional H-Bond	2.02
		H12	O	LEU101	H-Bond	Conventional H-Bond	2.17
		H17	O	THR156	H-Bond	Conventional H-Bond	2.84
		O2	C	ALA104	H-Bond	Carbon H-Bond	3.29
		/	N	VAL106	Electrostatic	Pi-Cation	4.12
		/	/	TRP109	Hydrophobic	Pi-Pi T-shaped	4.93
		/	/	HIS159	Hydrophobic	Pi-Pi T-shaped	4.57
		Cl1	/	ALA16	Hydrophobic	Alkyl	4.02
**4e**	-73.260	Cl1	/	Val155	Hydrophobic	Alkyl	4.36
		Cl1		TRP17	Hydrophobic	Pi-Alkyl	5.02
		6-ring	/	ARG14	Hydrophobic	Pi-Alkyl	4.38
		6-ring	/	VAL108	Hydrophobic	Pi-Alkyl	3.85
		6-ring	/	LEU124	Hydrophobic	Pi-Alkyl	4.28
		6-ring	/	ILE160	Hydrophobic	Pi-Alkyl	4.69
		6-ring	/	ILE163	Hydrophobic	Pi-Alkyl	4.76
		6-ring	/	PRO13	Hydrophobic	Pi-Alkyl	5.00
		6-ring	/	ARG14	Hydrophobic	Pi-Alkyl	4.46
		6-ring	/	CYS102	Hydrophobic	Pi-Alkyl	4.23
**Amphoterici**	-70.504	O17	HE	ARG14	H-Bond	Conventional H-Bond	2.61

**n B**	O7	HZ3	LYS117	H-Bond	Conventional H-Bond	3.03
	H4	O	TRP109	H-Bond	Conventional H-Bond	2.24
	H5	O	CYS111	H-Bond	Conventional H-Bond	1.47
	H7	O	LEU101	H-Bond	Conventional H-Bond	2.23
	H8	O	CYS102	H-Bond	Conventional H-Bond	2.64
	H9	OD1	ASN98	H-Bond	Conventional H-Bond	1.79
	O13	HA	ASN98	H-Bond	Carbon H-Bond	1.74
	O5	HE2	LYS117	H-Bond	Carbon H-Bond	2.46
	H32	O	ARG14	H-Bond	Carbon H-Bond	3.08
	O13	/	TRP17	Other	Pi-Lone Pair	2.92
	/	/	CYS102	Hydrophobic	Alkyl	4.77
	C21	/	CYS111	Hydrophobic	Alkyl	4.07
	C21	/	LYS117	Hydrophobic	Alkyl	3.99
	C41	C	LEU18	Hydrophobic	Alkyl	4.18
	C41	/	PRO50	Hydrophobic	Alkyl	4.29
	6-ring	/	TRP17	Hydrophobic	Pi-Alkyl	3.88
	C40	/	TRP17	Hydrophobic	Pi-Alkyl	4.19
	6-ring	/	TRP17	Hydrophobic	Pi-Alkyl	4.33
	6-ring	/	ILE163	Hydrophobic	Pi-Alkyl	5.38

b. Orientation and bonding interaction compound 4c in the active site cavity of Glutathione S-transferase (PDB: 4O92):

The docking results showed that the compound **4e** present the most potent antifungal agent confirmed by good docking MolDock Score of -73.260 kcal/mol and many molecular interactions with Glutathione S-transferase target from *C .krusei* vs. of the standard drug Amphotericin B with the score of -70.504 kcal/mol. Also, we note that this compound fit well in the binding pocket of the Glutathione S-transferase (fungal), which showed different types of interactions. In fact, compound **4e** established four strong hydrogen bonds and one weak, thirteen hydrophobic interactions and one electrostatic interaction with active site residues of the target protein (Table S3+Figure S32).

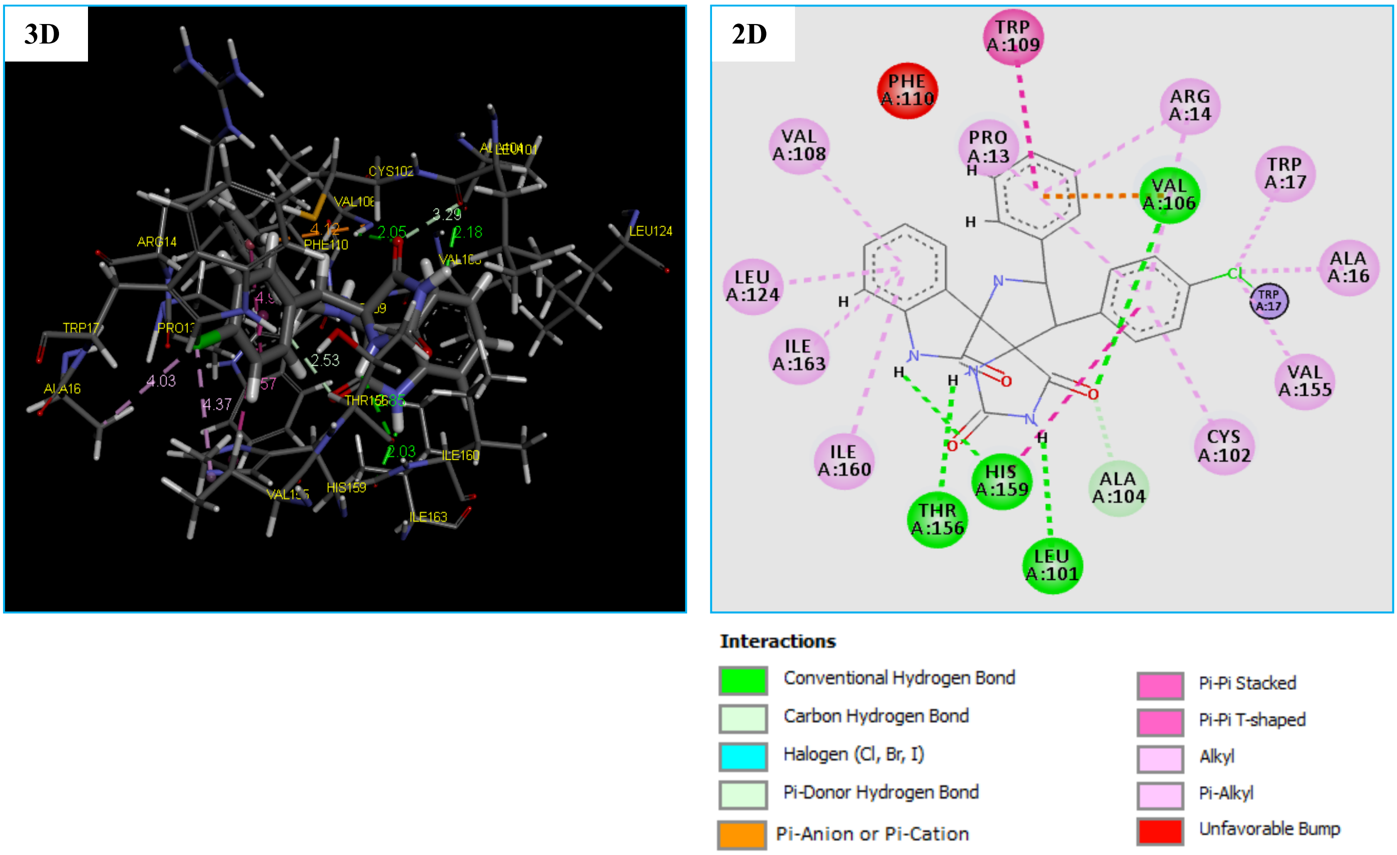

Figure S32. Representation 2D and 3D of interactions of the **4e** in the active site cavity of Glutathione S-transferase **(**PDB: **4O92)**

The strong hydrogen bonds were observed between the compound **4e** and active site residues: VAL106(2.04Å), HIS159(2.02Å), LEU101(2.17Å) and THR156(2.84Å), also the weak is formed with residue ALA104(3.29Å) of the target protein, the secreted aspartic protease (SAPs) (Figure S33). While thirteen hydrophobic interactions with active site residues: TRP109, HIS159, ALA16 Val155, TRP17, ARG14, VAL108, LEU124, ILE160, ILE163, PRO13, ARG14, and CYS102. One electrostatic interaction (Pi-Cation) with VAL106 was observed (Figure S33).

4.4. PDB binding structures of target enzymes

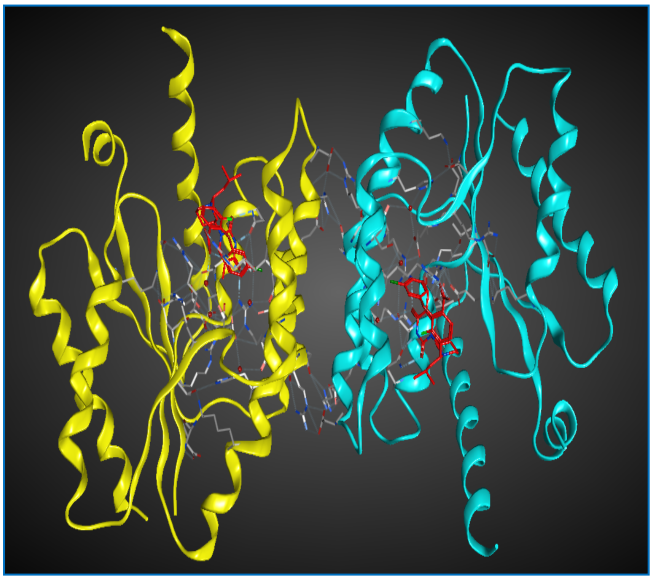

Figure S33: PDB Binding structure of Thymidylate Kinase (TMK) (PDB ID **4QGG**) from *S. aureus (Blue color: Chain A, Yellow color: Chain B, Red color: native ligands)*

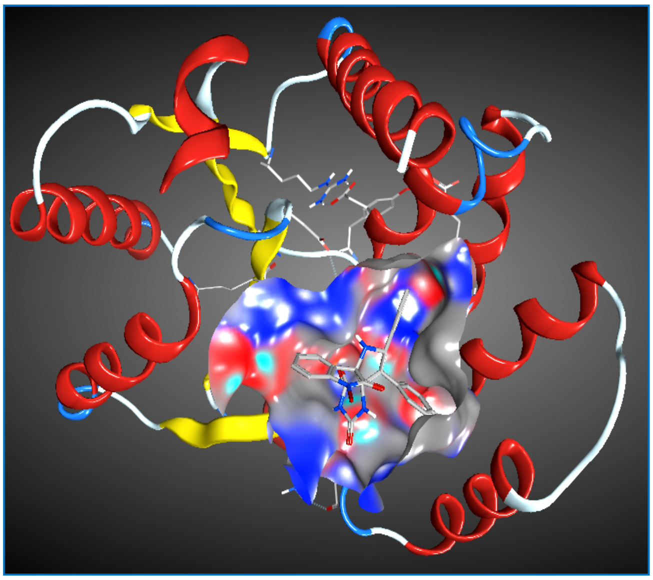

Figure S34: Active site residues of Thymidylate Kinase (TMK) (PDB ID **4QGG**) from *S. aureus after simplified (removed water molecules, ions,….)*

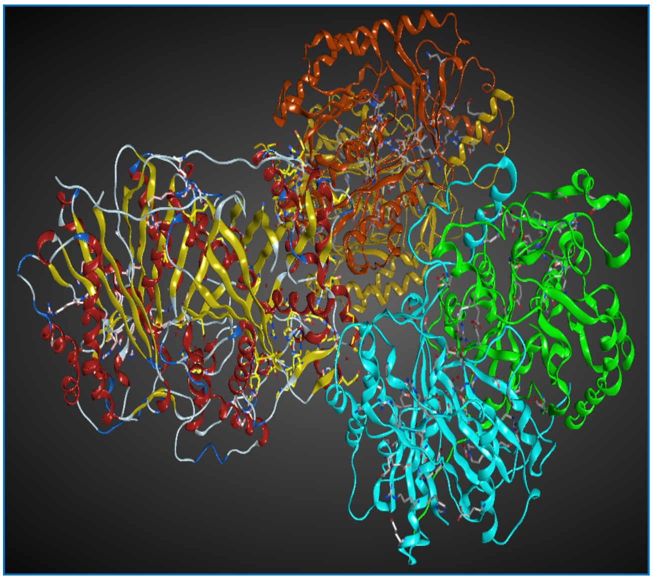

Figure S35: PDB Binding structure of (3-oxoacyl-[acyl-carrier-protein) synthase 3 (PDB ID **4EWP**) from *M. luteus (Blue color: Chain A, Yellow color: Chain B, Red color: Chain C, Green color: chain D, Brown: Chain E)*

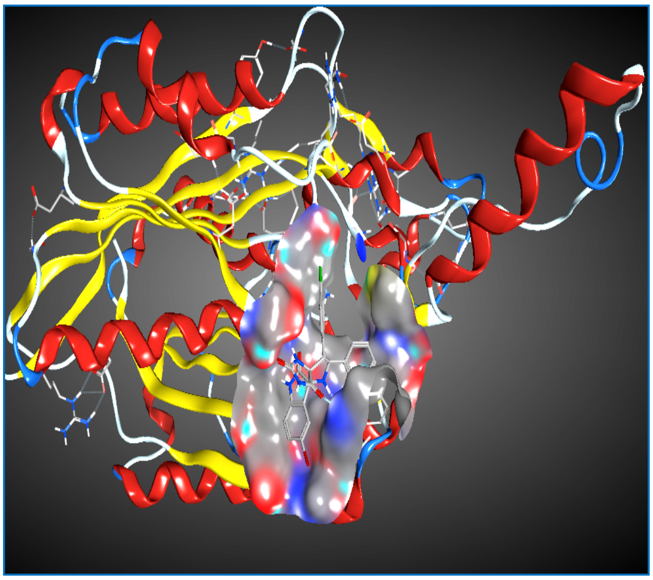

Figure S36: Active site residues of (3-oxoacyl-[acyl-carrier-protein) synthase 3 (PDB ID **4EWP**) from *M. luteus after simplified (removed water molecules, ions,….)*

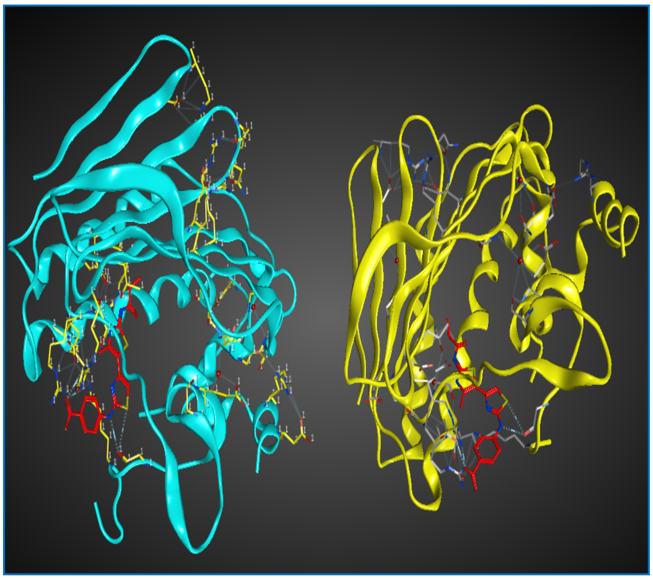

Figure S37: PDB Binding structure of DNA gyrase (PDB ID: **4DUH**) from *E. coli (Blue color: Chain A, Yellow color: Chain B, Red color: native ligands)*

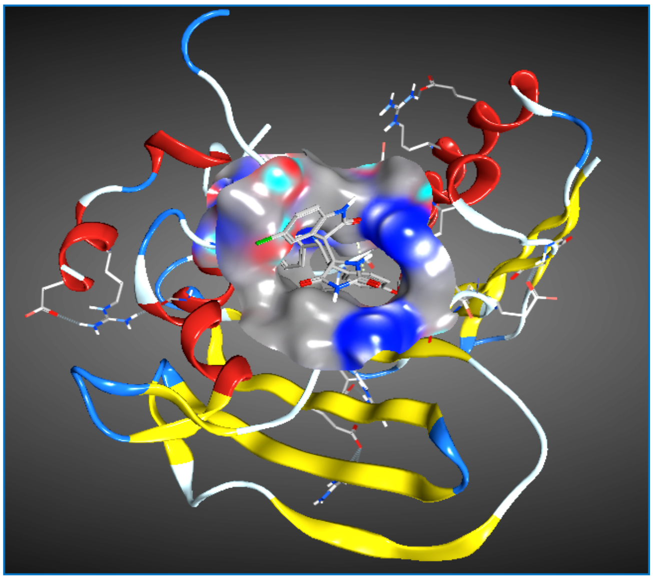

Figure S38: Active site residues of DNA gyrase (PDB: ID **4DUH**) from *E. coli after simplified (removed water molecules, ions,….)*

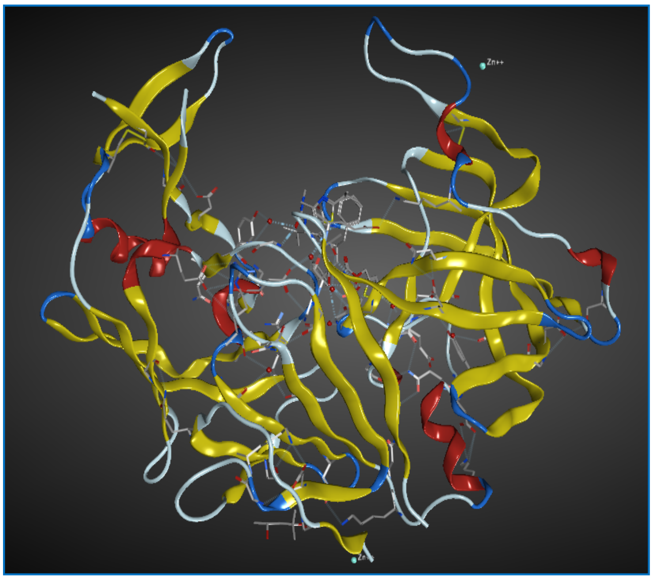

Figure S39: PDB Binding structure of secreted aspartic protease (PDB ID **3Q70**) from *C. albicans (Yellow color: Chain A, Red color: native ligands)*

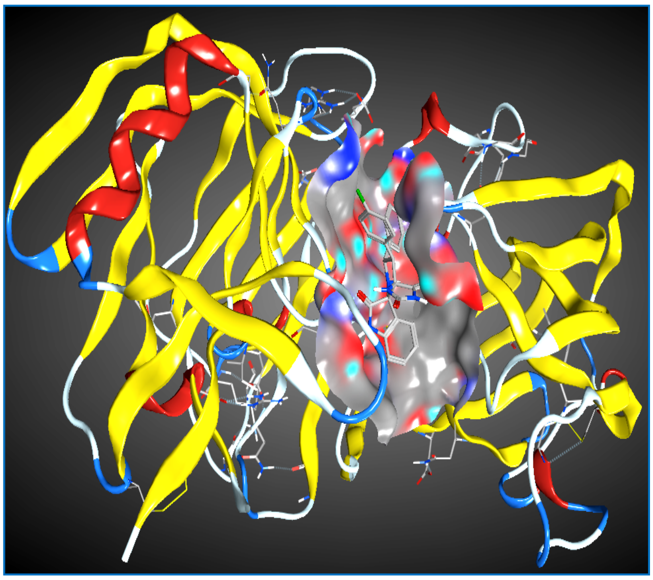

Figure S40: Active site residues of secreted aspartic protease (PDB ID **3Q70**) from *C. albicans after simplified (removed water molecules, ions,….)*

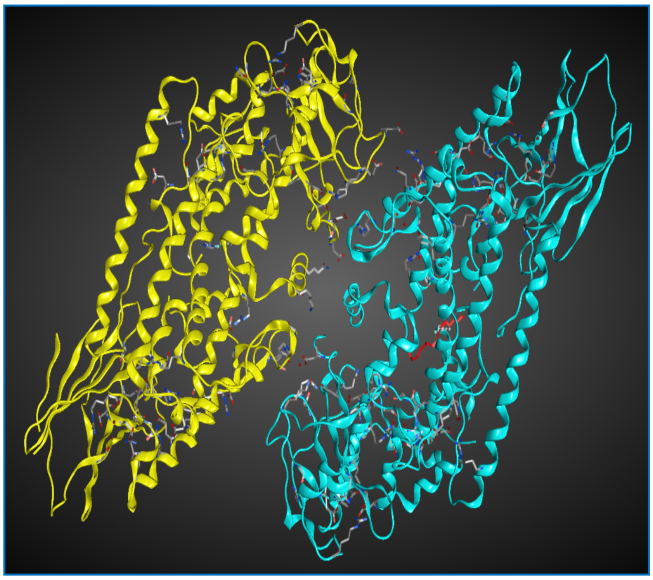

Figure S41: PDB Binding structure of Arachidonate 5-lipoxygenase (PDB ID **3V99**) from 5-LOX *(Blue color: Chain A, Yellow color: Chain B, Red color: native ligands)*

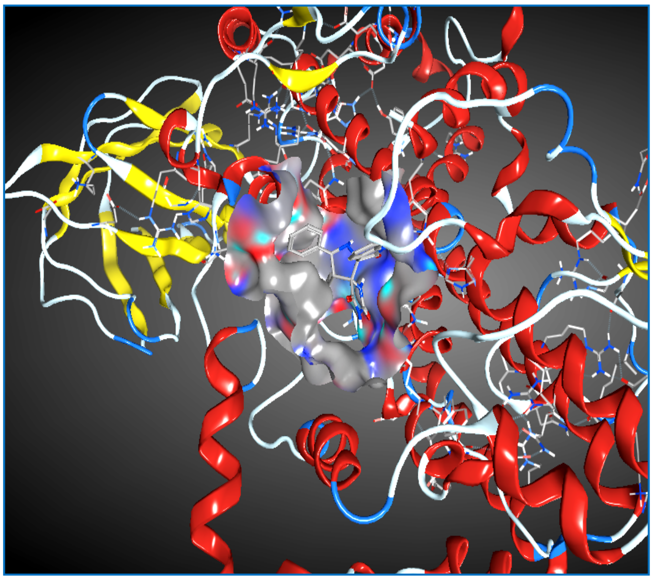

Figure S42: Active site residues of Arachidonate 5-lipoxygenase (PDB ID **3V99**) 5-LOX *after simplified (removed water molecules, ions,….)*
